# The impact of reducing car weight on global emissions: the future fleet in Great Britain

**DOI:** 10.1098/rsta.2016.0364

**Published:** 2017-05-01

**Authors:** André Cabrera Serrenho, Jonathan B. Norman, Julian M. Allwood

**Affiliations:** 1 Department of Engineering, University of Cambridge, Cambridge CB2 1PZ, UK; 2 Department of Mechanical Engineering, University of Bath, Bath BA2 7AY, UK

**Keywords:** weight, car, drivetrain, emissions, Great Britain

## Abstract

Current European policies define targets for future direct emissions of new car sales that foster a fast transition to electric drivetrain technologies. However, these targets do not consider the emissions produced in electricity generation and material production, and therefore fail to incentivise car manufacturers to consider the benefits of vehicle weight reduction. In this paper, we examine the potential benefits of limiting the average weight and altering the material composition of new cars in terms of global greenhouse gas emissions produced during the use phase, electricity generation and material production. We anticipate the emissions savings for the future car fleet in Great Britain until 2050 for various alternative futures, using a dynamic material flow analysis of ferrous metals and aluminium, and considering an evolving demand for car use. The results suggest that fostering vehicle weight reduction could produce greater cumulative emissions savings by 2050 than those obtained by incentivising a fast transition to electric drivetrains, unless there is an extreme decarbonization of the electricity grid. Savings promoted by weight reduction are immediate and do not depend on the pace of decarbonization of the electricity grid. Weight reduction may produce the greatest savings when mild steel in the car body is replaced with high-strength steel.

This article is part of the themed issue ‘Material demand reduction’.

## 1. Introduction

Over the last few centuries, human progress has been linked to increasing fuel combustion and to the consequent emissions of greenhouse gases (GHGs). These emissions account for more than two-thirds of global anthropogenic emissions, which continue to grow at an accelerated rate. The International Energy Agency (IEA) [1] reports that global transportation is responsible for a quarter of combustion emissions, and road transport alone accounts for most of these. In the United Kingdom (UK), transport emissions have increased from 21% in 1990 to 25% of direct combustion emissions in 2013, 94% of which are produced by road transport alone.

The IEA [2] estimates that global transport emissions should be reduced by more than 34% of current levels by 2050, in order to limit the global increase in average temperature to 2°C. In the European Union, current climate targets require a 60% reduction in transport emissions by 2050 [3]. As a result of the scale of this challenge, the IEA [4] estimates that most of the investments aimed at energy efficiency are expected to occur in the transport sector. Therefore, understanding the opportunities for transport emissions savings and quantifying the impact of interventions is essential for prioritising investments and to inform policy-makers. These efforts are particularly pertinent for road transport, which alone is responsible for around 24% of combustion emissions, and where thus there is potential to deliver substantial emissions savings. Pursuing these objectives could be facilitated by enhanced knowledge on the evolution of existing car fleets (hereafter defined as fleet dynamics), examining the impact of changing: material composition, weight and drivetrains of new cars, the energy system and consumer behaviour.

Current transport policies in the European Union (EU) define targets for average emissions of new cars. These should not be greater than 95 g CO_2_ km^−1^ by 2021. However, this average is weighted, giving additional credits to cars with emissions smaller than 50 g CO_2_ km^−1^—mainly electric cars [5]. This policy has been subject to substantial criticism. Smokers *et al*. [6] recall that current targets are set in terms of direct emissions produced by cars, which result in underestimating the impacts caused by electric and hybrid cars, because electricity generation and material production emissions are not considered. Gibson *et al*. [7] recommend that future targets should consider real-world emissions, rather than the emissions quantified during test cycles, as is the current practice. Moreover, the skewed view of average fleet emissions defined by current EU regulations dilutes the main goal of overall emissions savings and fails to incentivize car manufacturers to consider the potential benefits of weight reduction over electrification. In the UK, this is reflected in an average annual increase of 1% in the weight of new cars sold since 2000 [8].

The world's most fuel-efficient car to date has an extreme lightweight body of only 29 kg [9], so would policy targets limiting the average weight of cars be able to deliver the same emissions savings as current policies? This paper aims to provide answers to this question, by testing the impact on various limits to the average weight of cars, penetration of alternative drivetrains and behaviour change in the global emissions produced by the car fleet in Great Britain until 2050. This is tested using dynamic material flow analysis to assess the material stock demographics of the fleet and to estimate the required material flows and the global emissions associated with new cars, and fuel consumption required during the use phase of the fleet (§3). Estimated global emissions are anticipated for various alternative futures (§4), and their implications are discussed in §5.

## 2. Modelling the impacts of passenger cars

Many life cycle assessments (LCAs) have compared the impacts of alternative drivetrains with conventional internal combustion vehicles (ICVs). An example is the work done by Hawkins *et al*. [10], who have estimated that manufacturing and using an electric vehicle could result in less GHG emissions than an ICV. Other LCAs have compared various material compositions of cars: Kim *et al*. [11] have compared the effects of substituting mild steel with high-strength steel (HSS) or aluminium, and have anticipated a resulting 6–23% reduction in weight. These authors estimate similar savings from HSS and aluminium, although the electricity mix and the recycling rate have a substantial impact on the scale of savings achieved by aluminium substitution. A similar analysis has been conducted by Geyer [12], who anticipates savings of around 6 kg CO_2_ eq. kg^−1^ of mild steel replaced, and by Kelly *et al*. [13], who assessed the potential benefits of replacing steel with magnesium alloys and carbon-fibre-reinforced polymers (CFRPs). Lewis *et al*. [14] have tested the joint impacts of material substitution and the use of alternative drivetrains and have suggested that plug-in hybrid-electric vehicles (PHEVs) with mild steel replaced with HSS or aluminium would deliver the greatest emissions savings. However, Kim *et al*. [15], who reviewed 43 LCAs on material composition of vehicles or vehicle components, have identified that these analyses consider vehicle lifetimes ranging from 92 000 to 290 000 km, which results in a wide range of emissions savings.

LCA studies have compared individual vehicles of equivalent size and utility and thus they capture neither the diversity of the entire fleet nor their dynamics. This has been acknowledged by Field *et al*. [16], who showed that fleet-based approaches can lead to different results, and by Cheah [17], who has developed a fleet-based analysis of impacts of changes in material composition in the US fleet, capturing the effects of technological change in terms of fuel consumption and lightweighting of the fleet. Another fleet-based approach was introduced by Kagawa *et al*. [18], who have developed a dynamic age-cohort model to examine the impact of vehicle lifetime on total GHG emissions in Japan from 1990 to 2000. Later, Pauliuk *et al*. [19] used a dynamic material flow analysis to estimate future energy use and GHG emissions of the Chinese car fleet until 2050. These analyses consider the fleet dynamics, and so the delay of introducing a new technology on the entire fleet, but they do not capture the impacts of material substitution in future recycling options, and the consequences in terms of global GHG emissions. This aspect was examined by Modaresi *et al*. [20], who used a dynamic material flow analysis to explore various material substitution options for the global fleet and have estimated the impact of the resulting weight reduction on global emissions savings. This analysis was able to capture the use phase, material production and electricity generation emissions, and the material recycling potentials.

The above-mentioned analyses provide compelling insights into impacts produced by the use of alternative material compositions and weight reduction in the future car fleet, but do not compare the performance of these strategies to the current policies that foster a fast transition to electric drivetrain technologies. Moreover, some dynamic material flow analyses consider the influence of mileages and vehicle lifetimes, although the estimation of the composition of future stocks and their impacts is often based on postulated rates of vehicle ownership and distance travelled. Therefore, existing approaches consider neither the impact of behaviour changes affecting car occupancy and intensity of use, nor the influence of a transition to faster modes of transport (such as high-speed rail and aviation) in future demand for car use. Assessing the impact of these variables may enable the formulation of additional policy interventions aimed at minimizing the emissions and material demand required for the future demand for transportation services. This assessment is particularly pertinent for developed economies that will have to manage their existing fleets. In this paper, we propose to model the composition and dynamics of the material stock of the car fleet, considering future trends in the demand for car use, GHG emissions from material production, electricity generation and fuel combustion of car use. This model enables a comparison of global GHG emissions savings produced by weight reduction, by using alternative drivetrain technologies, and by fostering behaviour change.

## 3. Delivering future transportation

**Figure 1.**
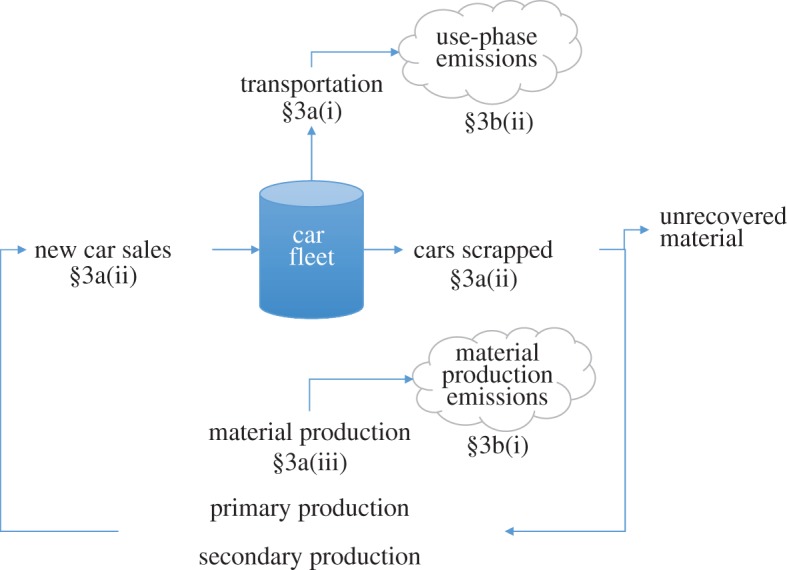
Simplified representation of the model architecture. Further details are provided in the electronic supplementary material. (Online version in colour.)

Testing the impact of vehicle weight reduction, alternative drivetrains and behaviour change on global emissions requires a dynamic assessment of the future composition and impacts of the British car fleet. Existing literature described in §2 suggests that the most reliable method to assess the car fleet is by developing a dynamic material flow analysis that models material stocks over time. In this analysis, this is accomplished by designing such a model, considering variable demand for car use and a set of assumptions on the future material composition of cars, drivetrain technologies, patterns of car use, electricity generation and material production and recycling technologies. Figure 1 shows a simplified representation of the modelling approach used in this paper. This approach comprises a sequential description of the methods and assumptions used to estimate future demand for car use (§3a(i)), the number of cars that will be required to supply that demand (§3a(ii)), how much material will be required to manufacture those cars (§3a(iii)) and how much energy and emissions will be used and produced by those cars (§3b). This tool can then be used to test the sensitivity of global emissions to various targets on future car weight, penetration of alternative drivetrains and behaviour change between 2015 and 2050 (§4).

### (a) The demographics of the car fleet

The composition of the future car fleet depends both on pre-existing stocks and on future mileage demand. While the current and historical composition of material stocks in the car fleet can be determined with reasonable accuracy based on available literature and official statistics, the future demand for car use needs to be estimated. The following sections discuss an option for estimating the future demand for mileage (§3a(i)). This then allows an estimation of the required number of cars (§3a(ii)) and material flows (§3a(iii)) between 2015 and 2050.

#### (i) Future demand for transport

The demand for transportation services has been examined extensively by national statistics in many countries. This enables an assessment of the main empirical patterns of transport demand. Fortunately, these patterns seem to be strong enough to sustain a few assumptions on the future mileage demand by mode. Schäfer *et al*. [21] present two budgets that seem to have defined transportation in the past, which have been verified both for contemporaneous countries at different development stages and for single countries over time:

— The time travel budget: on average, each person on the planet uses 1.1 h per day for travel. This result has also been verified by Metz [22] for the UK alone.
— The money travel budget: people spend on average a constant share of their income for transportation services (measured in passenger km *per capita* for all transportation modes).

**Figure 2.**
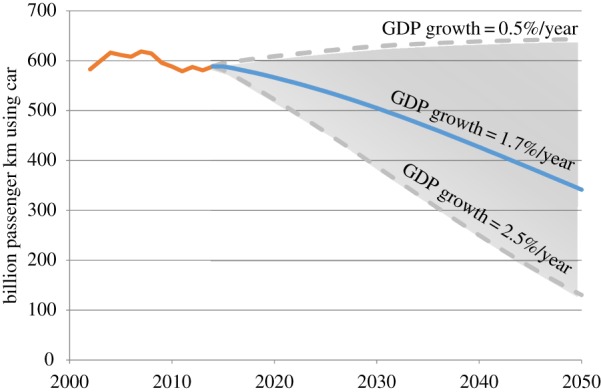
Demand for car use in Great Britain. Historical values are shown in orange-red and have been obtained from the Department for Transport [25]. Projected values are represented in blue (moderate GDP growth) and grey-dashed lines (low and high GDP growth). (Online version in colour.)

The empirical evidence of the existence of travel budgets limits the range of possible futures. Thus, future growth in GDP *per capita* and a limited time travel budget result in increasing demand for faster transportation modes, as demonstrated by Schäfer *et al*. [21]. A limit seems to exist when all passengers use the fastest transportation mode during their entire time travel budget. Schäfer *et al*. [21] suggest that such a trend would result in a modal shift from the slower transportation modes to the fastest. This reasoning is explored by Schäfer *et al*. [23], who have estimated the future demand for transport by mode across 11 world regions, based on forecasts of future population and GDP *per capita*. This model enables the estimation of future car-use demand for Great Britain until 2050, as a function of future GDP *per capita* and population. Population projections for Great Britain can be obtained from the Office for National Statistics [24]; however, future GDP is difficult to estimate and has a substantial influence on the estimates of car-use demand. Thus, three alternative GDP time series are used in this work, based on three alternative annual growth rates: 0.5%, 1.7% (as estimated by Schäfer *et al*. [23] for Western Europe) and 2.5%. Figure 2 shows the projected demand for car use that results from using Schäfer's model [23] and the above GDP growth rates. The Department for Transport [25] reports the historical demand for car travel, which is also shown in figure 2.

#### (ii) Car fleet requirements

The demand for car use is expected to decrease according to figure 2, as the demand for high-speed transport modes increases, but what would be the impact of this trend on the future British car fleet?

The demand for car use (measured in passenger km) is supplied by a stock of cars capable of travelling a required distance over a certain time period and carrying the required number of passengers. Therefore, car-use intensity (defined as the amount of time in a week a car is travelling) and car occupancy are key metrics of the stock's intensity of use. Passenger km (*PK*) can be decomposed as a function of stock size (*S*, in number of cars) and these intensity metrics as follows:
3.1
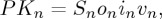

where *o* represents the average car occupancy (in passengers per car), *i* is the car-use intensity and *v* is the average speed of each trip in the year *n*.

Each year, new car sales (*P*_in,*n*_) should be as much as required to keep the total number of cars in service (*S_n_* obtained from equation (3.1)) able to provide the required levels of service, taking into account the number of cars scrapped (*P*_out_):
3.2
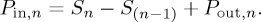


The number of cars scrapped (*P*_out_) is determined using a Weibull distribution whose parameters have been estimated from historical records of licensed stock [26]. This distribution is often used in dynamic material flow analysis [20] and is found to be well suited to model car lifetimes [27]. Further details on the modelling approach and parameters are provided in the electronic supplementary material.

The energy input required per km travelled depends on the drivetrain technologies of cars. This requires a disaggregation of the car fleet into various drivetrains in order to capture the total energy use and emissions produced by the fleet. In this model the following drivetrains have been considered: ICVs, hybrid-electric vehicles (HEVs), PHEVs, battery electric vehicles (EVs) and hydrogen fuel cell vehicles (FCVs), with further disaggregation into petrol and diesel variable where applicable. Currently, the fleet is comprised almost exclusively ICVs, but the impacts of shifting to alternative drivetrains is assessed by testing different levels of penetration, which are detailed in §4 of the electronic supplementary material.

#### (iii) Material requirements

The previous sections described how the future car sales are estimated (*P*_in,*n*_). This allows the estimation of the annual requirements of each material *k* for new cars. The required mass (*M_k_*_,*n*_) is estimated according to equation (3.3), given the share of each drivetrain technology *d* in car sales (*θ_d_*_,*n*_), the average mass of material (*M_k_*_,*d*,*n*_) used by drivetrain *d* and the manufacturing yield losses (*γ_k_*_,*n*_) of that material in the year *n*:
3.3
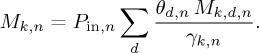

Reducing the average weight of cars can be achieved either by reducing the size of cars (downsizing) or by changing the material composition of cars, resulting in lighter cars with the same size. These strategies can be applied independently and this analysis explores the impacts of both.

The scale of weight reduction achieved by changing the material composition of cars is limited by the extent of material substitution and by material choice. Although composite materials, such as CFRPs, are able to offer excellent mechanical properties for lightweighting, their production is substantially more energy- and emissions-intensive than for conventional metals, and there are limited options for their recycling. Therefore, this analysis considers only alternative compositions using mild steel, HSS, cast iron, wrought aluminium and cast aluminium. Section 3 of the electronic supplementary material file provides an extensive discussion on the alternative future compositions considered and historical estimates.

Downsizing is defined by a scale factor (*ε*) applied to the mass of all materials in a car. The weight of a downsized car (*M*′) is then defined according to equation (3.4) as a function of mass of each material (*M_k_*):
3.4
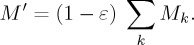


### (b) The impacts of transportation

The use and manufacture of future cars estimated above generate global GHG emissions. These occur during the production of materials used in car manufacturing, and are produced in the combustion of fuels during car use, and in generating the electricity used in material production, electric drivetrains or for hydrogen production. In this analysis, we estimate only GHG emissions associated with the production of mild steel, HSS, cast iron, cast aluminium and wrought aluminium, which currently account for more than 60% of all material emissions in cars. These and the use-phase emissions are detailed in the following sections.

#### (i) Material production

GHG emissions due to materials used in manufactured cars depend on the emissions intensity of each material production process. For a year *n*, the material production emissions (*E*_mat,*n*_) are estimated according to equation (3.5),
3.5
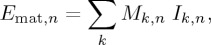

where the emissions intensity of each material (*I_k_*_,*n*_) depends on the share of primary production (*δ_k_*_,*n*_) and the emissions intensity of primary (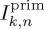) and secondary (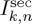) production processes (equation (3.6)),
3.6
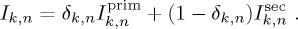


Serrenho *et al*. [28] concluded that only around 5% of steel used in vehicles purchased in the UK had been produced by the UK steel industry. This seems to be the case for most materials used in cars, and thus global averages of energy and emissions intensity of material production are more appropriate to reflect the impacts associated with new cars sales in Britain. Modaresi *et al*. [20] have estimated global emissions intensities of primary and secondary material production until 2050, covering all processes up to cold rolling for steel, up to extrusion for wrought aluminium and up to the cast part for cast iron and aluminium. These values are assumed in this analysis, considering the shift to steel recycling estimated by Pauliuk *et al*. [29] to 2050, but no variation in future aluminium production shares, because Kim *et al*. [11] consider unlikely the transition to a closed-loop recycling for aluminium over the next few decades. The impacts of future material production used in cars are estimated in this work considering the yield losses occurring throughout the materials' supply chains. We assumed the same current yield losses and that the losses in non-cast materials could be halved through material efficiency strategies by 2050 [30].

#### (ii) Use phase

Use-phase emissions comprise both GHGs produced by the combustion of petrol and diesel in ICVs, HEVs and PHEVs, and the emissions produced in electricity generation that is directly used by EVs, HEVs and PHEVs. FCVs use hydrogen that is assumed to be produced by natural gas reforming [31]. Car mileage and fuel or electricity consumption per mile enable the estimation of use-phase emissions. However, in any given year the use of drivetrains, average weight and efficiency depend on car age, and therefore use-phase emissions (*E*_use,*n*_) should consider each age cohort of the fleet, according to equation (3.7):
3.7
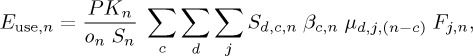

where *PK_n_*/*o_n_S_n_* is the average annual mileage of each car, *S_d_*_,*c*,*n*_ is the number of cars using drivetrain *d*, *µ_d_*_,*j*,*t*_ is the fuel or electricity consumption per mile for a car with drivetrain *d* and manufactured in the year *t*, *F_j_*_,*n*_ is emissions produced per unit of fuel/electricity *j*, and *β_c_*_,*n*_ is a mileage weighting factor for cars with age *c*. The energy use per mile for each car (*µ_d_*_,*j*,*t*_) depends also on the weight. This relationship between car weight and fuel economy is well known in the existing literature. The electronic supplementary material file provides details on this relationship and on future grid emissions.

## 4. Global emissions savings obtained by alternative policies

In this analysis, the effect of three variables on global GHG emissions is assessed: the average weight of cars, the use of alternative drivetrains and consumer behaviour. Several options for these three variables have been considered (§3) along with estimates of the future demand for car use. However, the effect of car weight and the use of alternative drivetrains are not independent from one another, and so these two variables deserve a more careful analysis. For this reason, this section describes a sensitivity analysis of global GHG emissions to the varying average weight of cars and share of use of alternative drivetrains (§4a), and a scenario analysis to test the influence of all the remaining variables (§4b).

### (a) The influence of car weight and electric drivetrains in global emissions

**Figure 3.**
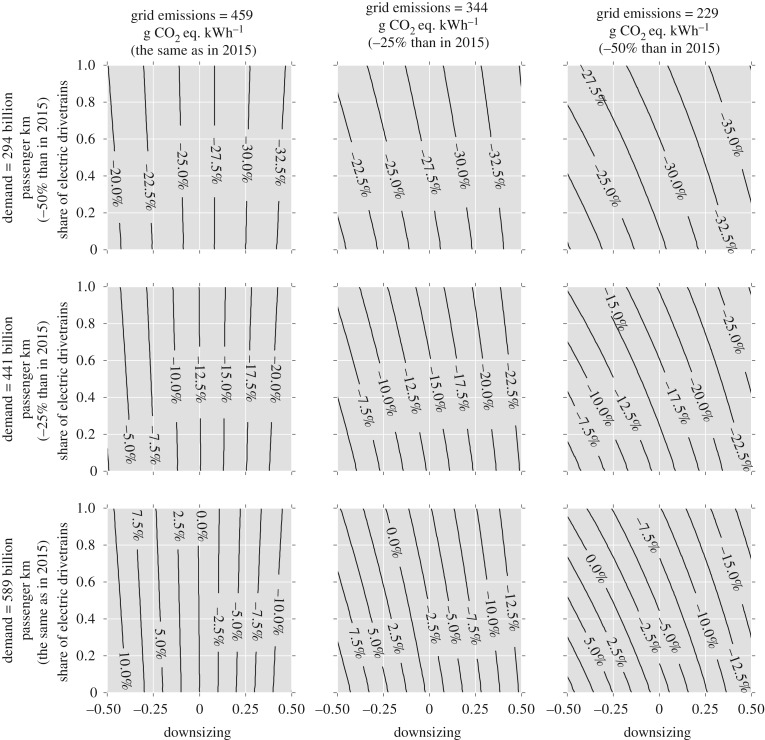
Sensitivity analysis: cumulative GHG emissions (2015–2050) of the British fleet for different shares of electric drivetrains and downsizing of car sales, for various levels of car-use demand and decarbonization of the electricity grid in 2050. Downsizing is the scaling factor *ε* defined in equation (3.4). Contours represent the percentage change in cumulative GHG emissions compared with the cumulative emissions in 2015–2050 that would have been produced if there were no changes in demand, electricity grid, share of drivetrains or average weight of cars.

**Figure 4.**
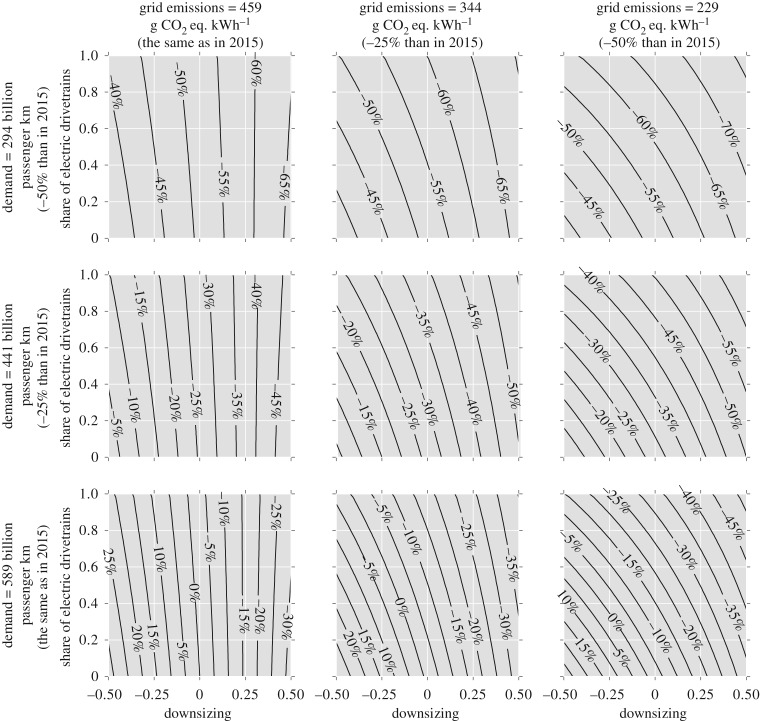
Sensitivity analysis: GHG emissions in 2050 of the British fleet for different shares of electric drivetrains and downsizing of car sales, for various levels of car-use demand and decarbonization of the electricity grid in 2050. Downsizing is the scaling factor *ε* defined in equation (3.4). Contours represent the percentage change in GHG emissions compared to the global emissions produced by the British fleet in 2015.

The interdependence of car weight and the use of alternative drivetrains is particularly notable in the case of electric drivetrains, because these imply heavier cars for the same size and the potential benefits of using electricity may offset the additional energy required to move extra weight and to produce extra materials. In addition, the use of electric drivetrains shifts the production of emissions from the car to the electricity grid. To explore this relationship the model described in §3 is used to test the sensitivity of global GHG emissions to varying the share of electric drivetrains and the average weight of cars. The effect of varying car weight is explored by varying downsizing, and the effect of electric drivetrains is explored by varying the share of cars using electricity from the grid (EVs and PHEVs). This is shown in figures 3 and 4 for three different levels of car-use demand and three different levels of decarbonization of the electricity grid.

Figures 3 and 4 show nearly vertical contours when grid emissions are the same as in 2015 and only more marked curvatures when grid emissions have been decarbonized to 50% of 2015 levels by 2050. This shows the limited contribution of EVs and PHEVs to reduce global GHG emissions. The greatest emissions savings seem to be achieved only with significant weight reduction. Figure 3 also suggests that there is a limited scope to reduce significantly cumulative emissions from 2015 to 2050, even for the more ambitious scenarios of weight reduction, use of electric drivetrains and grid decarbonization. Only a substantial reduction in the demand for car use seems to lead to the highest cumulative emissions savings. Although current policies are focused on annual GHG emissions, this analysis in terms of cumulative emissions offers an indication of the long-term impact of different options on the climate. However, considering annual emissions in 2050 (figure 4), the greatest emissions savings seem to be produced when there is both weight reduction and electrification of the fleet, although the potential of EVs and PHEVs to reduce annual emissions is only enhanced at high levels of grid decarbonization.

### (b) Assessing future scenarios for the British car fleet

Figures 3 and 4 reveal the sensitivity of global GHG emissions to variations in the share of electric drivetrain and downsizing. However, the average weight of cars can also be reduced by changing the material composition. This effect is examined by estimating the global GHG emissions produced by the use of various material composition strategies, different levels of penetration of alternative drivetrains, and also the impact of changes in occupancy and intensity of car use on global GHG emissions. To test the isolated and combined effects of these variables, a set of 13 alternative scenarios is defined and global GHG emissions are estimated for each one (table 1). A reference scenario assumes the trend in demand shown in figure 2 for an annual GDP growth rate of 1.7%, but no changes in weight, material composition and share of drivetrain technologies between 2015 and 2050. All scenarios assume the same material production technologies and electricity generation emissions, according to the description in §§3a and 3b and in §§3, 4 and 6 of the electronic supplementary material.

**Table 1.** Definition of scenarios of alternative material composition, use of drivetrain technologies and behaviour patterns. All scenarios assume a demand for car use of 342 billion passenger km (projected demand for the central option shown in figure 2) and UK electricity grid emissions of 292 g CO_2_ eq. kWh^−1^ in 2050.

scenarios		material composition^a^	share of drivetrain technologies^b^	occupancy	intensity of use	weight variation in 2050^c^
reference		same as in 2015	same as in 2015	same trend as in 2002–2015	same trend as in 2002–2015	0%
material composition	steel-intensive	steel-intensive	same as in 2015	same trend as in 2002–2015	same trend as in 2002–2015	−20%
	aluminium-intensive	aluminium-intensive				−23%
	aluminium-extreme	aluminium-extreme				−33%
alternative drivetrains	with EVs and PHEVs only	same as in 2015	electric dominant	same trend as in 2002–2015	same trend as in 2002–2015	+13%
	with a balanced penetration		balanced			+10%
constant occupancy and intensity of use (constant OIU)		same as in 2015	same as in 2015	same as in 2015	same as in 2015	0%
+20% OIU				increase in 20% by 2050	increase in 20% by 2050	0%
alternative drivetrains with steel-intensive		steel-intensive	electric dominant	same trend as in 2002–2015	same trend as in 2002–2015	0%
alternative drivetrains with aluminium-intensive		aluminium-intensive				−5%
alternative drivetrains with aluminium-extreme		aluminium-extreme				−14%
alternative drivetrains with steel-intensive and constant OIU		steel-intensive	electric dominant	same as in 2015	same as in 2015	0%
alternative drivetrains with steel-intensive and +20% OIU				increase in 20% by 2050	increase in 20% by 2050	0%

^a^The alternative compositions are defined in §3.3 of the electronic supplementary material for each drivetrain. Besides changes in material composition, a 10% downsizing in 2050 car sales is considered.

^b^The shares of alternative drivetrain for electric dominant and balanced scenarios are defined in §4 of the electronic supplementary material.

^c^Resulting change in average weight of new car sales in 2050 compared with 2015.

The impact of replacing mild steel with either HSS or aluminium in new cars is examined in three scenarios, keeping constant the share of drivetrain technologies and assuming the current trends in car occupancy and intensity of use. The impact of changing drivetrain technologies in new car sales is examined in two additional scenarios, using different levels of penetration of alternative drivetrains as defined in §4 of the electronic supplementary material. The impact of alternative trends in car occupancy and intensity of use are also tested. Two additional scenarios compare the effects of stopping the current trend on decreasing occupancy and intensity of car use, and a 20% increase which would correspond to increasing occupancy to the current levels observed in Hungary, and return car-use intensity to the same levels verified in 2000 in Great Britain. The combined effects of material composition, use of drivetrain technologies and behaviour patterns are also examined.

**Figure 5.**
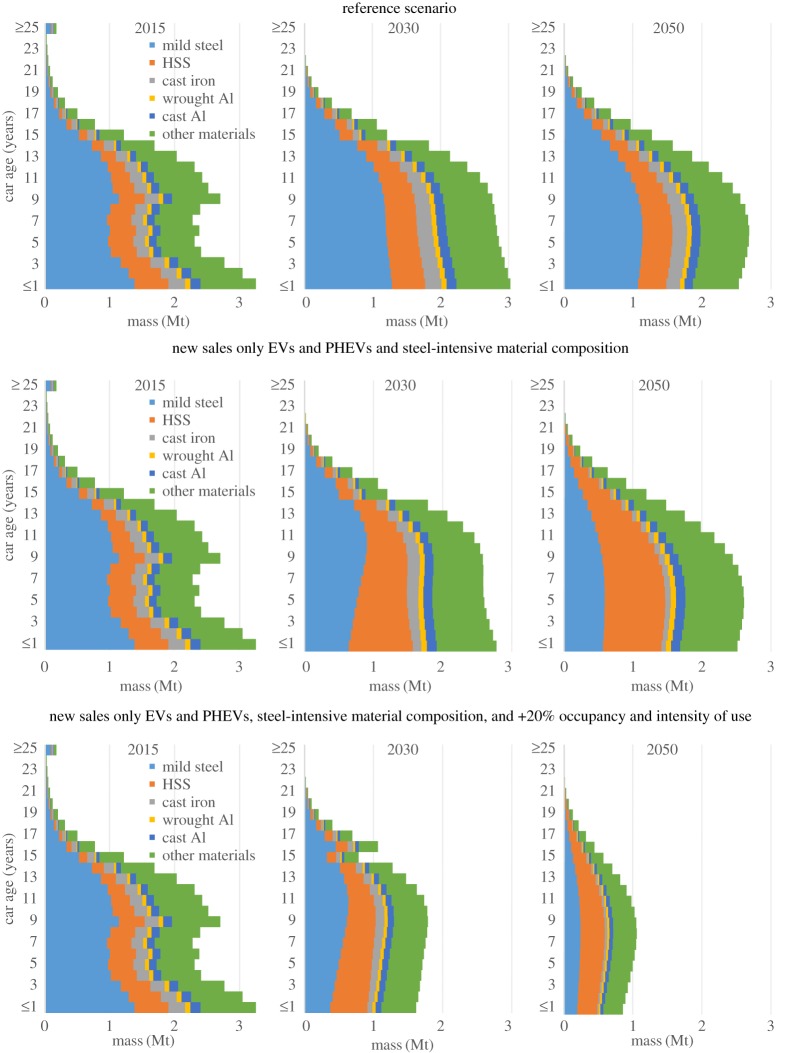
Material stock demographics in 2015, 2030 and 2050 for three alternative scenarios.

The scenarios described in table 1 lead to different demographics of the car fleet. Using the representation suggested by Serrenho *et al*. [8], figure 5 shows the material stock demographics of the current fleet and also the anticipated demographics in 2030 and 2050 for the reference scenario, for new car sales comprising only EVs and PHEVs with steel-intensive material composition, and also for a combination of this with an increase in 20% of occupancy and intensity of use. For the reference scenario (keeping current weight and material composition of cars), a nearly constant material demand is anticipated until the 2030s, decreasing afterwards as a result of a decreasing demand for car use. Fostering new car sales comprising EVs and PHEVs with steel-intensive material composition results in a similar pace of total material demand. Despite the significant substitution of mild steel by HSS visible in 2030 and 2050, the penetration of heavier vehicles using alternative drivetrains seems to offset part of the weight reduction gains of this material substitution. When these strategies are combined with an increase in car occupancy and intensity of use, a substantial reduction is notable in the material accumulation from the early 2020s owing to the decrease in the number of cars required. This scenario would result in a much smaller and lighter stock, although with an older fleet.

**Figure 6.**
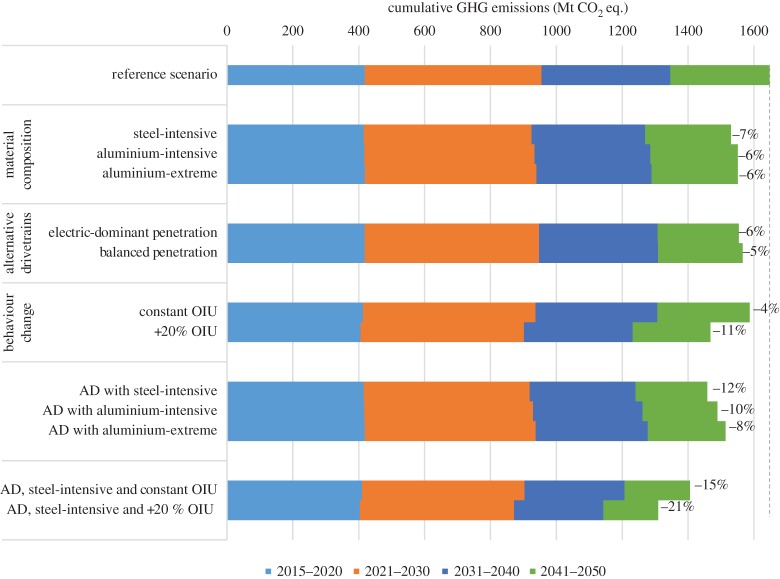
Cumulative GHG emissions between 2015 and 2050 for each pathway, split by time. Percentage emissions reduction compared with the reference scenario. OIU, occupancy and intensity of use; AD, alternative drivetrains.

Figure 6 shows the total cumulative GHG emissions from material production (aluminium and ferrous metals only) and the use phase of cars in Britain between 2015 and 2050 for the scenarios defined in table 1. Figure 6 anticipates potential emissions savings for each alternative scenario, testing different options for material composition, use of alternative drivetrains, behaviour change and combinations of these, relative to the reference scenario. The greatest potential savings are estimated to be achieved only when alternative material composition, drivetrains and higher occupancy and intensity of car use are combined together, although their magnitude is limited as they result in savings no greater than 21% of the reference scenario. This is a consequence of the inertia of the current stock and the time lag required to produce emissions savings from gradual changes in new vehicle sales. This results in a dominant effect of emissions produced from 2015 to 2030, which account for around two-thirds of all emissions until 2050.

**Figure 7.**
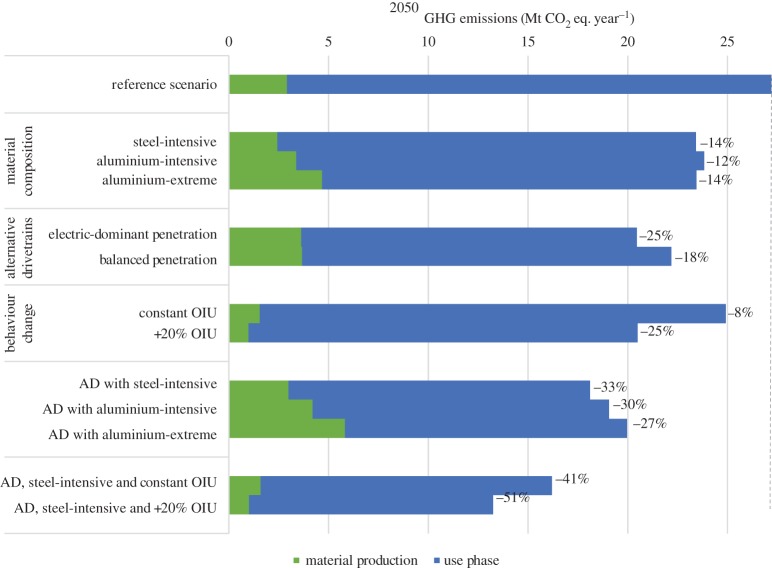
GHG emissions in 2050 by pathway and percentage emissions reduction compared with the reference scenario. OIU, occupancy and intensity of use; AD, alternative drivetrains.

Figure 7 shows the anticipated annual GHG emissions for 2050 from material production (ferrous metals and aluminium only) and use phase for each scenario defined in table 1. In 2050, it is expected that most of the fleet would comprise cars with an alternative material composition and drivetrain. This results in higher relative emissions savings for the annual emissions in 2050 relative to the reference scenario.

## 5. Discussion

The results shown in §4 suggest that fostering vehicle weight reduction could produce greater cumulative emissions savings by 2050 than those obtained by current policies that incentivise the use of electric drivetrains, unless there is an extreme decarbonization of the electricity grid (e.g. more than 50%). Reducing the average weight of new cars produces immediate net emissions savings, because lighter cars lead to less energy requirements and emissions during the use phase. If the UK road transport emissions are to be reduced to 60% of 1990 levels (or around 70% of 2015 levels) by 2050, as is the target of current policies for the entire European transport sector [3], that would require a substantial reduction in car weight and demand for car use (figure 4). Such a strategy would also have the effect of reducing material emissions during car production, which are not covered by the above target. This can be combined with a higher penetration of electric cars if the grid is substantially decarbonized.

Current policies foster the fast penetration of electric drivetrain technologies in car sales [32]. Our results estimate that such policies could produce negligible global emissions savings, unless the UK electricity grid is decarbonized by approximately 50% or greater by 2050. This is a consequence of the inertia of the current fleet, which comprises almost exclusively ICVs. Moreover, the use of electric drivetrains leads to heavier cars, and thus this strategy, even when combined with alternative material composition, seems to offset the emissions abatement of any material savings. However, only when most of the fleet comprises EVs and PHEVs and the electricity grid has been substantially decarbonized could greater emissions savings be achieved, as suggested by figures 4 and 7. It is worth noting that such a decarbonized grid must also be an expanded one, because a switch to electric drivetrains expands the total demand for electricity generation, thus making grid decarbonization even more challenging.

Policies that foster vehicle weight reduction are able to deliver immediate GHG emissions savings (figures 3 and 4). Our results reveal the inertia of the current stock, which limits the potential cumulative emissions savings. Even if the UK electricity grid follows the pace of decarbonization of the New Policies scenario of the IEA [4], reaching –50% of CO_2_ eq. kWh^−1^ in 2050, and if the demand for car use evolves according to the central trajectory of figure 2, the potential for cumulative emissions savings seems to be only 11%, in the case of both EVs and PHEVs with steel-intensive composition. This highlights the pertinence of interventions capable of delivering fast emissions savings that do not depend on the pace of decarbonization of the electricity grid.

Alternative policies that set weight reduction targets for cars could be difficult to implement. Weight reduction may raise safety concerns, and thus large cars may be preferred by consumers. However, the emissions saving potential of such policies would be secured if downsizing of all classes of cars is promoted. For example, targets could be set in terms of the average sales of each manufacturer, in line with current targets on direct emissions.

In this work, the effects of weight reduction are tested using various material compositions. A more intensive use of aluminium in cars seems to result in slightly higher global emissions than replacing most of mild steel by HSS. Although the pursuit of aluminium substitution would result in lighter vehicles and thus in less use-phase emissions, the impacts of aluminium production seem to offset these gains. This is because of the higher emissions intensity and limited recyclability of aluminium compared with steel.

Small changes in the patterns of car use may result in substantial emissions savings. Over the last decade, cars in Great Britain have tended to carry fewer passengers and are idle for more time. The results shown in §4 suggest that stopping these trends alone could result in almost the same cumulative GHG savings that are obtained by electrifying the fleet by 2050. Policies that incentivise shared ownership or the deployment of autonomous driving could result in an increase in car occupancy and intensity of use. If such policies lead to an increase in 20% of these variables by 2050 (equivalent to increasing occupancy to the current levels observed in Hungary, and return car-use intensity to same levels verified in 2000 in Great Britain), that alone could lead to cumulative emissions savings of around 11% by 2050, almost twice as much as is estimated as a result of alternative material compositions and drivetrains alone. However, the anticipated material demographics of the fleet suggests that these policies would heighten the ageing that is expected to occur in the fleet, mainly motived by a projected decrease in car use. This could lead to higher costs for maintenance and repair in the future.

This analysis provides insights into the consequences of alternative futures for the British car fleet, but it has some limitations. Our estimates of aluminium production and electricity use in PHEVs and EVs depend on the assumptions on the future electricity grid both in Britain and globally, although these were based on official reports. Therefore, more decarbonized electricity grids could produce more favourable results for high use of aluminium in cars and for higher shares of electric drivetrains. However, the results shown in figures 3–7 suggest that aluminium production and electric vehicles could only provide substantial savings compared with weight reduction if the electricity grid is severely decarbonized by 2050.

The results presented in figures 5–7 result from a projected decline in the demand for car use. Although this trajectory is acknowledged in the literature, it does not consider the effect of increasing the number of activities that passengers may be able to do while travelling owing to better conditions and comfort or even to the use of driverless cars. This change in the services provided by cars may alter the time travel budget and thus the projected demand for services. In addition, the projected reduction in the number of cars on the roads could decrease the time spent travelling, incentivising car use. Car ownership can also be motivated by other factors such as status, and not necessarily by service demand, which could result in global emissions being greater than those estimated in this work. This analysis considers only the emissions during the use phase and the production of ferrous metals and aluminium, but it excludes other materials, manufacturing and end-of-life treatments and car maintenance, although the magnitude of the emissions considered accounts for the majority of impacts of car use and manufacturing.

## Supplementary Material

Model, scenarios, and supporting data
