# Supplementary material for: The impact of reducing car weight on global emissions: the future fleet in Great Britain
Source: Philos Trans A Math Phys Eng Sci. 2017 May 1;375(2095):20160364. doi: 10.1098/rsta.2016.0364 (PMC5415645; doi:10.1098/rsta.2016.0364)
Supplement: Model, scenarios, and supporting data [file rsta20160364supp1.pdf]

SUPPLEMENTARY INFORMATION

THE IMPACT OF REDUCING CAR WEIGHT ON GLOBAL EMISSIONS: THE FUTURE FLEET IN GREAT BRITAIN

André Cabrera Serrenho [a], Jonathan B. Norman [b], Julian M. Allwood [a]

[a] Department of Engineering, University of Cambridge, United Kingdom.

[b] Department of Mechanical Engineering, University of Bath, United Kingdom.

TABLE OF CONTENTS

1. Stocks' model 2
1.1. Modelling approach 2
1.2. Initial stock 2
1.3. Lifetime of stock 3
2. Demand for transportation service 4
3. Material composition of cars 5
3.1. Mass and material composition of the fleet in 2015 5
3.2. Historic mass and material composition trends 7
3.3. Material composition scenarios 7
4. Drivetrain technologies 8
5. Fuel economy and car weight 10
6. Emissions factors for fuels and electricity 11
7. Distance travelled by car age 12
8. Annual composition of the car fleet 14
8.1. Reference scenario 14
8.2. New sales only comprised of EVs and PHEVs with steel-intensive material composition 23
8.3. New sales only comprised of EVs and PHEVs with steel-intensive material composition +20% of occupancy and intensity of use 32
References 41

1. STOCKS' MODEL

In this section the car fleet model for Great Britain is described (section 1.1) and the initial stock composition is characterised (section 1.2).

1.1. Modelling approach

The approach taken here is a cohort-lifetime-based, stock-driven dynamic material flow analysis. The model uses a number of distinct layers to represent the various stocks and flows, these are the product layer, the material layer and the energy and emissions layer. Figure 1 shows a simplified representation of the model architecture with flows of product (black arrows) and materials (grey arrows) shown. Energy and emissions are considered for the stage of "Stocks in use" and "Material Production" as indicated by solid outlines in Figure 1. This basic structure of the model can be applied to other products and materials. Scrap and primary resource use are not explicitly tracked in the current contribution.

Different age cohorts, materials and product variants are explicitly modelled and tracked over the time period of the study. The base year of the model is 2015 and results are reported to 2050.

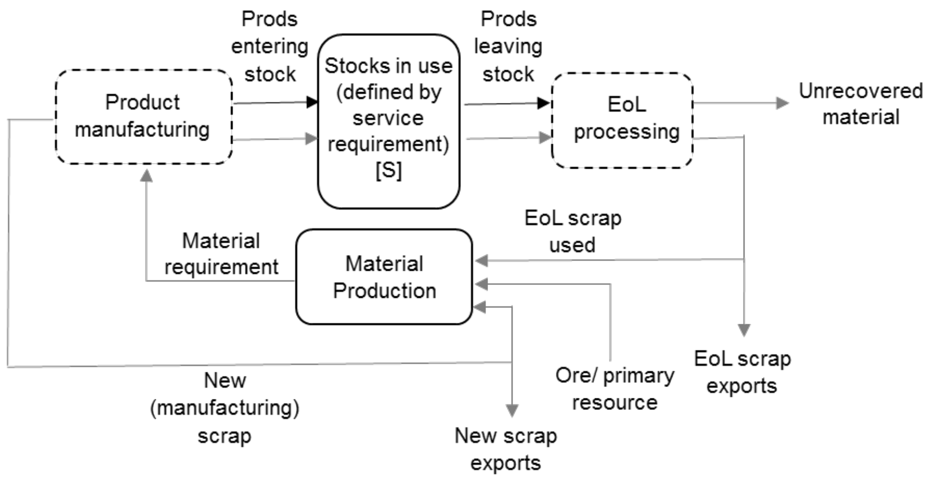

Figure 1. Generic model architecture, black arrows show product flows and grey arrows material flows. Dotted lines indicate stages where the energy and emissions are not explicitly represented.

1.2. Initial stock

Data on the current stock of British passenger cars was extracted from statistics available from the Department for Transport (2016b). This allowed the size of stock in each age cohort and for each drive technology to be estimated for the base year, 2015. Only the stock that contributes to service provision was included. Therefore, vehicles with a statutory off road notification (SORN) were not included. Vehicles registered before-1970 and those of an unknown registration year (which includes imported vehicles and pre-1970 vehicles subsequently re-registered) were discounted as without a year of registration they couldn't be included in the model. These comprise around 2% of the total stock. It is also important to note when interpreting the statistics that not all vehicles counted as registered enter the stock (Lewis, 2016) and relying solely on registration figures will overestimate historic stock additions. Here historical stock age data was used to determine the vehicles entering stock in each year.

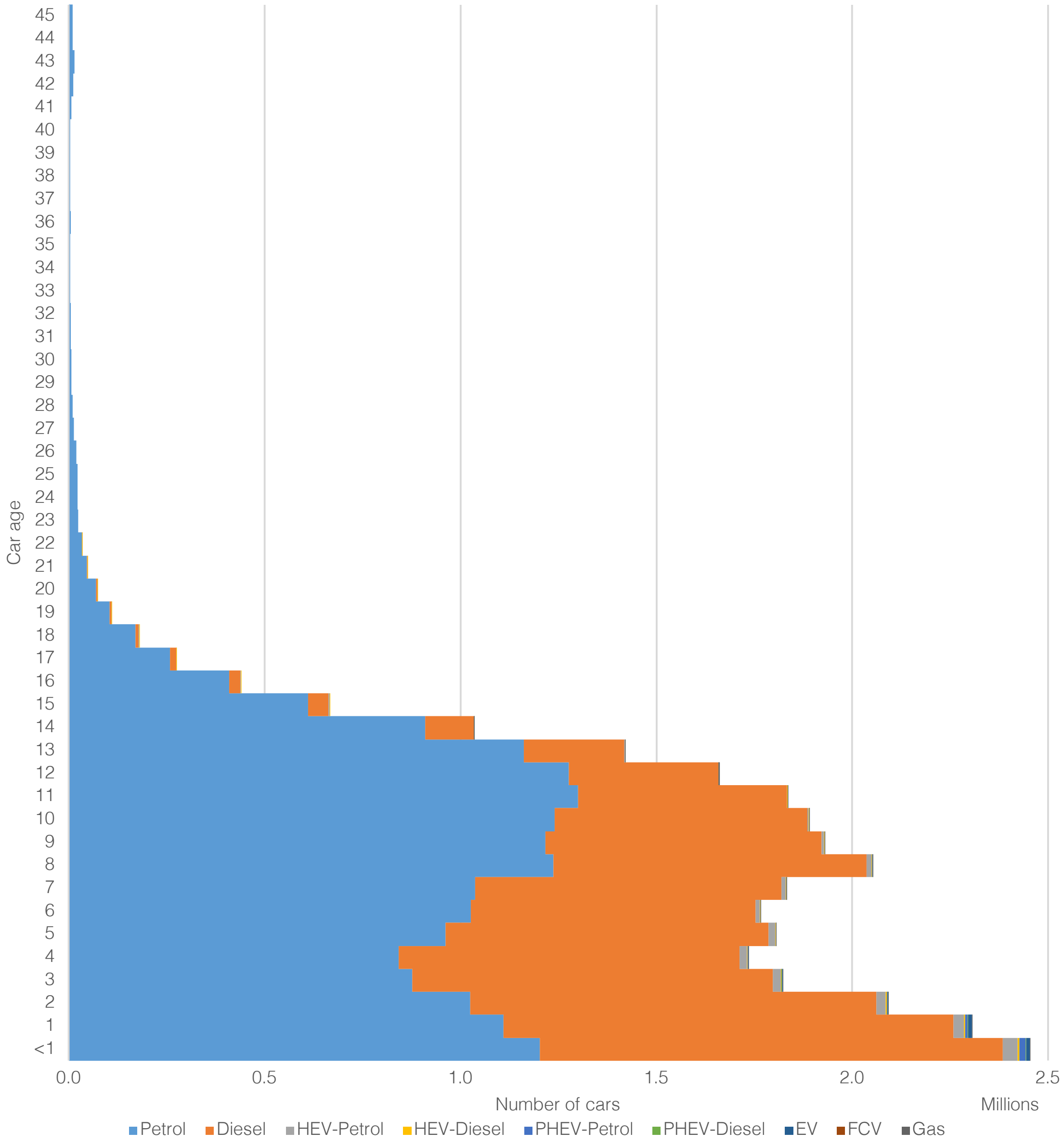

Figure 2. Composition of the car fleet in Great Britain in 2015.

1.3. Lifetime of stock

A Weibull distribution was used to estimate stock removals in each age cohort. Such a distribution is found to be well suited to car lifetimes (Oguchi *et al.*, 2015). The survival function of the stock in vehicles is shown in Figure 3, comparing historic data (Department for Transport, 2016b) and the modelling approach used here. For the Weibull distribution the shape and scale parameters were estimated as 4.4 and 14.6, respectively. This results in an average lifetime of 13.3 years.

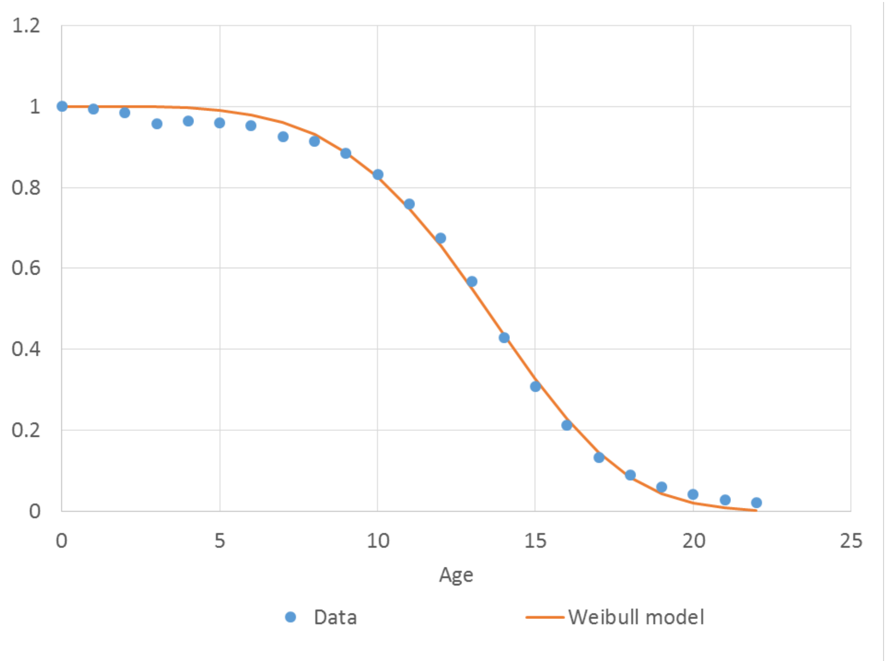

Figure 3. Survival function of passenger cars, as calculated from data and modelled using the Weibull distribution.

2. DEMAND FOR TRANSPORTATION SERVICE

In this analysis, an estimate of future demand for car-use is required in order to estimate the required number of cars in stock every year. This demand estimate was based on Schäfer's model (Schäfer *et al.*, 2000) that anticipates future demand by transport mode as a function of the GDP per capita, so that future demand is constrained by the time and money travel budgets (these are detailed in Schäfer *et al.* (2009) and in section 3.1 of the article main text).

Three alternative scenarios for future GDP in the UK have been considered, all of them by applying constant annual growth rates of 0.5%, 1.7% (the average growth rate considered by Schäfer *et al.* (2000) for Western Europe), and 2.5% to current values. These three growth rates determined future GDP and consequently three alternative scenarios of car-use demand shown in Table 1 and in Figure 2 of the article main text.

Table 1. Estimated demand for car-use in the UK.

Year	Demand for car-use [passenger km per capita]		
	GDP growth rate = 0.5% per year	GDP growth rate = 1.7% per year	GDP growth rate = 2.5% per year
2015	9408	9303	9189
2016	9386	9175	8948
2017	9364	9046	8706
2018	9342	8915	8464
2019	9320	8783	8220
2020	9298	8651	7977
2021	9275	8517	7734
2022	9252	8382	7491
2023	9229	8246	7249
2024	9206	8110	7008
2025	9182	7972	6768
2026	9159	7835	6529
2027	9135	7696	6292
2028	9110	7557	6056
2029	9086	7418	5823
2030	9062	7279	5592
2031	9037	7139	5364

Year	Demand for car-use [passenger km per capita]		
	GDP growth rate = 0.5% per year	GDP growth rate = 1.7% per year	GDP growth rate = 2.5% per year
2032	9012	6999	5138
2033	8987	6859	4915
2034	8961	6719	4695
2035	8936	6579	4478
2036	8910	6439	4265
2037	8884	6299	4055
2038	8858	6160	3849
2039	8832	6021	3648
2040	8805	5882	3450
2041	8779	5744	3257
2042	8752	5606	3068
2043	8725	5469	2883
2044	8698	5333	2704
2045	8670	5197	2529
2046	8643	5062	2359
2047	8615	4927	2195
2048	8587	4794	2035
2049	8559	4662	1881
2050	8531	4530	1733

3. MATERIAL COMPOSITION OF CARS

In this section the weight and material composition of the past, current, and future fleet is characterised. The historical composition and average weight is characterised in sections 3.1 and 3.2. The future material composition and weight of new car sales is addressed in section 3.3, based on the scenarios defined by Modaresi *et al.* (2014).

3.1. Mass and material composition of the fleet in 2015

Mass and material composition in 2015 was estimated for each drivetrain technology with no split between petrol and diesel variants. Therefore, values were calculated for internal combustion vehicles (ICV), hybrid-electric vehicles (HEV), plug-in hybrid-electric vehicles (PHEV), electric vehicles (EV) and fuel cell vehicles (FCV). The mass was split between various materials: mild and other steels, high strength steel (HSS) and advanced high strength steel, cast iron, wrought aluminium, cast aluminium and other. Mass and material composition was also defined for different vehicle subsystems: body and closures; chassis and suspension; powertrain; and interior and misc. The baseline values represent the average characteristics of a new car sold in Great Britain in 2015 for each drivetrain.

ICV mass was estimated based on historical data for the mass of new passenger cars sold in Great Britain (GB) (ICCT, 2016). The split of this mass between materials and vehicle subsystems was based on that reported by Modaresi *et al.* (2014). Data for other drivetrains has less availablity., thus the GREET database (ANL, 2015) was used to estimate the mass and material composition of non-ICVs (including a split by vehicle subsystem). The GREET database reports detailed data on mass and material use for the drivetrains of interest. The database is specific to US vehicles, which on average have a higher mass than those in GB. The mass of non-ICVs is therefore scaled to a GB equivalent, based on their relative mass compared to an ICV as reported by the GREET database and the baseline mass for an ICV. The split of the mass of non-ICVs between materials and vehicle subsystems was estimated by combining information from the GREET database with the baseline specification of an ICV. The specification of baseline vehicles is shown in Table 2 to Table 6.

Table 2. Baseline mass of ICVs (values in kg).

	Mild and other steels	HSS + AHSS	Cast iron	Wrought Al	Cast Al	Other	Total
Body and closures	213	174	0	7	0	43	437
Chassis and suspension	195	40	17	9	22	35	318
Powertrain	94	0	90	4	39	103	331
Interior and misc.	58	0	0	12	2	166	238
Total	560	213	107	32	64	347	1323

Table 3. Baseline mass of HEVs (values in kg).

	Mild and other steels	HSS + AHSS	Cast iron	Wrought Al	Cast Al	Other	Total
Body and closures	239	195	0	8	0	48	491
Chassis and suspension	214	43	18	10	24	38	348
Powertrain	157	0	45	16	53	63	334
Interior and misc.	58	0	0	12	2	184	257
Total	668	239	63	46	80	334	1430

Table 4. Baseline mass of PHEVs (values in kg).

	Mild and other steels	HSS + AHSS	Cast iron	Wrought Al	Cast Al	Other	Total
Body and closures	219	179	0	7	0	44	450
Chassis and suspension	202	41	17	10	23	36	330
Powertrain	143	0	37	15	46	57	298
Interior and misc.	58	0	0	12	2	319	392
Total	623	220	54	44	72	457	1470

Table 5. Baseline mass of EVs (values in kg).

	Mild and other steels	HSS + AHSS	Cast iron	Wrought Al	Cast Al	Other	Total
Body and closures	356	291	0	12	0	72	731
Chassis and suspension	308	63	26	15	35	56	502
Powertrain	75	0	0	9	70	84	238
Interior and misc.	58	0	0	12	2	352	425
Total	797	354	26	48	108	563	1896

Table 6. Baseline mass of FCVs (values in kg).

	Mild and other steels	HSS + AHSS	Cast iron	Wrought Al	Cast Al	Other	Total
Body and closures	282	230	0	10	0	57	579
Chassis and suspension	229	46	19	11	26	41	373
Powertrain	114	0	0	59	38	194	404
Interior and misc.	58	0	0	12	2	184	256
Total	682	277	19	91	66	476	1612

3.2. Historic mass and material composition trends

99% of the current fleet in GB are ICVs. The average weight of new cars sold in 2001–2014 was adapted from ICCT (2016). Longer term historic trends were based on extrapolating the trends seen over this period back to 1990. The average weight of new sales in 1975 was estimated at 882 kg and a constant growth rate assumed from 1975 to 1990. Previous to 1975 the average weight was assumed constant. These longer-term trends were based on those seen in the EU over this time period (Zachariadis, 2006). The split of different materials in comprising the mass of ICVs was based on data from Modaresi ***et al.*** (2014) for the period 1980–2010. From 2010 to 2015 a linear interpolation to the baseline values was assumed. Previous to 1980 the material split was assumed constant. A split between wrought aluminium and cast aluminium was not available historically (a value for total aluminium was available from Modaresi ***et al.*** (2014)), therefore the split between different grades of aluminium was assumed the same as in the baseline case for all years. Historic average weight and material composition of new sales of ICVs are shown in Figure 4. Previous to the base year (2015) it was assumed that the total mass of non-ICVs followed the same trends as seen in ICVs, although these cars accounted for less than 1% of the fleet. The material composition of non-ICVs was assumed constant at baseline values.

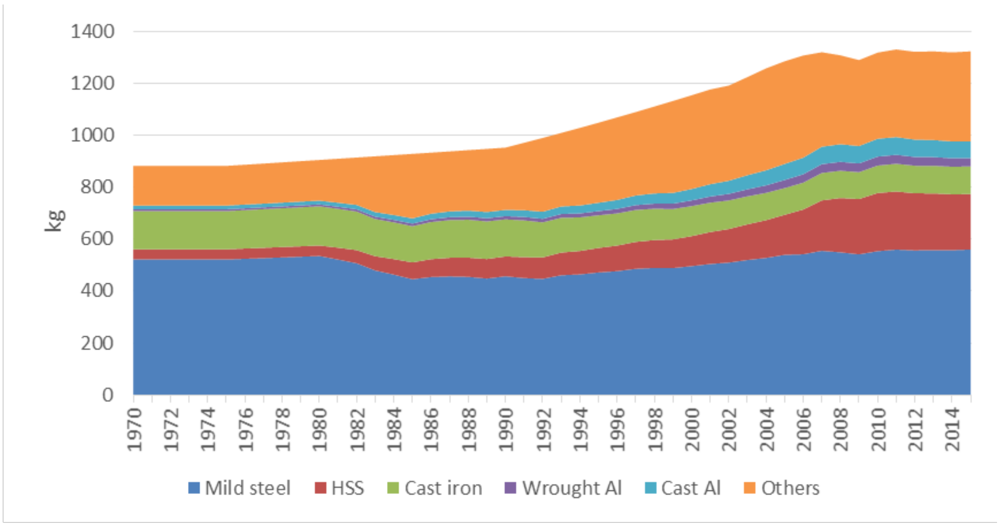

Figure 4. Average mass and material composition of new car sold in Great Britain, 1970–2015.

3.3. Material composition scenarios

Modaresi ***et al.*** (2014) have examined the effect of material substitution in reducing the average weight of the global car fleet, testing the following alternative material compositions:

- **Steel-intensive:** 100% of mild steel in the body is replaced with high strength steel (HSS), 25% of mild steel in the chassis is replaced with HSS.

- **Aluminium-intensive:** 100% of mild steel in the body is replaced with wrought aluminium, 25% of mild steel in the chassis is replaced with wrought aluminium.
- **Aluminium-extreme:** 100% of mild steel in the body is replaced with wrought aluminium, 25% of mild steel and 100% of cast iron in the chassis is replaced with wrought aluminium and cast aluminium respectively. In the powertrain 50% of cast iron is replaced with cast aluminium. In the interior 100% of mild steel is replaced with wrought aluminium.

The options above consider only material substitution, but additional weight reduction can be achieved by reducing the average size of cars. Cheah (2008, 2010) estimates that it is possible to shift future car sales to smaller size categories than today and that would result in a 10% mass reduction. Figure 5 shows the current and three alternative future material compositions of new car sales by drivetrain technology, considering all the options above applied to the British fleet. These average compositions of new car were assumed to be realised by 2030, plateauing thereafter (in line with assumptions by Modaresi *et al.* (2014)).

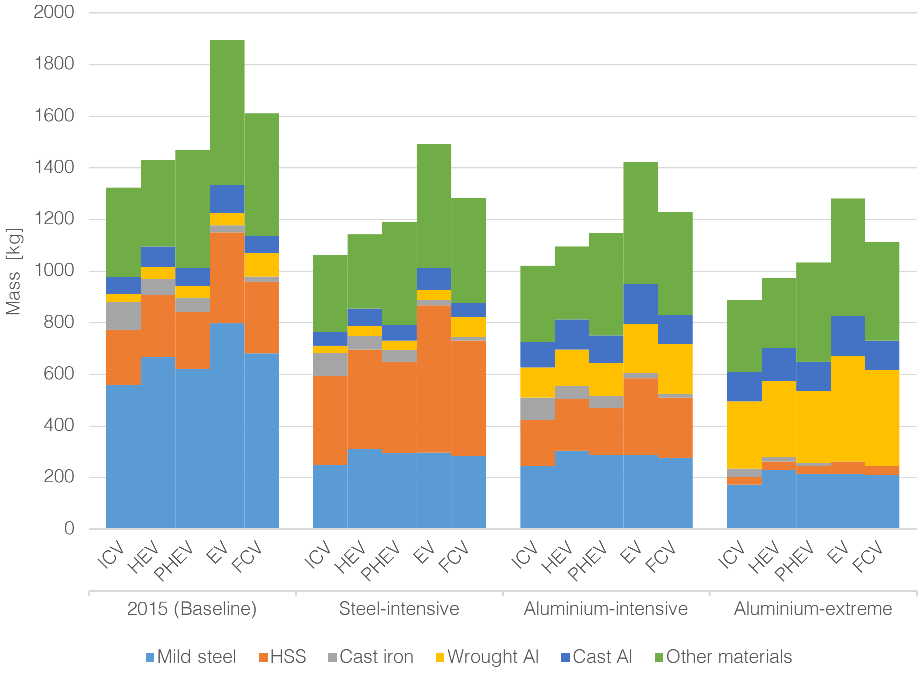

Figure 5. Material composition split by drivetrain technology and lightweighting scenario.

4. DRIVETRAIN TECHNOLOGIES

Three scenarios of future vehicle sales were included:

- a **plateau** at 2015 values,
- an **electric dominant**,
- and a **balanced** scenario.

Until 2030 the electric dominant and balanced scenarios are assumed to follow the same trajectory. Future sales in 2020 and 2030 were based on the Central Scenario of the Fifth Carbon Budget of the Committee on Climate Change (CCC, 2015). This estimated the proportion of new sales in 2030 comprising EV and PHEV at 25% and 35% respectively. No uptake of FCV was expected in this time period. It was assumed that of the remainder of sales (ICV plus HEV) HEV increased to 5% of combined ICV and HEV sales by 2020 and 10% of these from 2030 onwards (it was around 1.7% in 2015). The rest of new vehicles sales comprise ICV. This approach estimates the proportion of sales as shown in Figure 6 and Table 7.

In 2050 under the electric dominant scenario all new cars are either EV or PHEV, with the split between these technologies based on the CCC scenario for 2030. In 2050 this means new sales are 42% EV and 58% PHEV. In the balanced scenario sales in 2050 are split across the range of technologies. FCV sales are assumed to grow to 20% of total sales by 2050, whilst EV comprise 35%, PHEV 20% and the remainder of sales are split between ICV and HEV (22.5% and 2.5% respectively). This scenario builds on the trends seen to 2030 in the CCC Central Scenario (CCC, 2015), whilst introducing FCV and retaining a level of ICV. The split of sales for both scenarios in 2050 can be seen in Figure 6 and Table 7.

In between the periods shown in Table 7 relative sales of PHEV, EV, FCV and HEV are assumed to grow at a constant annual rate, with the remainder of sales comprising ICV. ICV, HEV and PHEV are further split into petrol and diesel variants of the drivetrain. These are assumed to stay at approximately baseline levels throughout the period of analysis: in ICV petrol and diesel variants are split equally, in HEV and PHEV petrol variants comprise 90% of sales.

When exploring the effect of different levels of alternative drivetrain uptake (see Figure 4 and Figure 5 in the main paper) the electric dominant scenario is scaled. An implementation level of 1.0 implies the scenario is as discussed above and shown in Table 7, whilst an implementation level of 0.0 implies that the BAU scenario is used. When the implementation level is between 0.0 and 1.0 the values for EV and PHEV sales in 2020, 2030 and 2050 shown in Table 7 are scaled according to this factor and ICV and HEV sales calculated as above. For example, with an implementation level of 0.5 EV/ PHEV uptake in 2020, 2030 and 2050 are: 1.9%/2.7%, 12.5%/17.5% and 21.0%, 29.0% respectively (half the values shown in Table 7). The remainder of sales are ICV and HEV, with HEV comprising 5% of these in 2020 and 10% in 2030 and 2050. Other years sales are calculated based on constant growth rates between these periods.

It should be noted that the scenarios discussed here are not predications of the future of vehicle sales, but tools to explore the implications of different possibilities and explore general trends in sales rather than year-to-year changes.

Table 7. Future passenger car sales split for electric dominant and balanced scenario, 2020, 2030 and 2050. 2015 sales shown for comparison.

	ICV	HEV	PHEV	EV	FCV
2015	97.2%	1.7%	0.7%	0.4%	0.0%
2020	86.5%	4.6%	5.3%	3.8%	0.0%
2030	36.0%	4.0%	35.0%	25.0%	0.0%
2050 Electric dominant	0.0%	0.0%	58.0%	42.0%	0.0%
2050 Balanced	22.5%	2.5%	20.0%	35.0%	20.0%

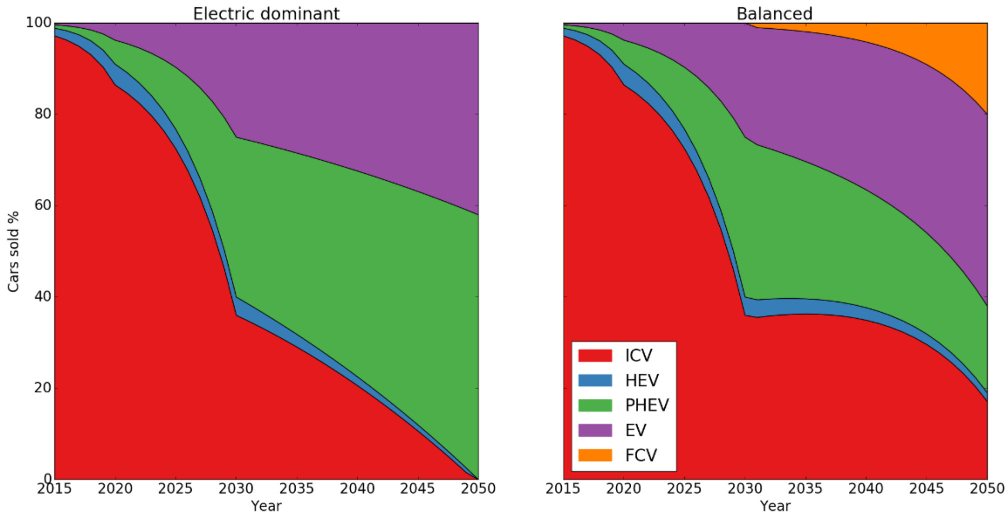

Figure 6. Share of drivetrain technologies in car sales for the electric dominant and balanced scenarios.

5. FUEL ECONOMY AND CAR WEIGHT

The average fuel consumption of petrol and diesel cars sold in the UK is reported annually for the baseline and historic years (DECC, 2015; Department for Transport, 2015). This reported consumption is based on the New European Drive Cycle (NEDC). Real world fuel consumption differs from this reported figure with the difference between NEDC and real-world figures seemingly growing over the last decade (Mock *et al.*, 2012; Mock *et al.*, 2014). Such a gap between reported and real world efficiency is a long recognised issue (Schipper *et al.*, 1993). A realistic fuel consumption value was adopted here. The correction factor between reported and real life fuel consumption was estimated based on information from the International Council for Clean Transportation (Mock *et al.*, 2014). The ratio between real life and NEDC emissions is shown in Figure 7. Real world efficiency (ε') is estimated from NEDC reported efficiency (ε) as $\varepsilon' = \varepsilon(1 + f)$, where f is the correction factor according to Figure 7.

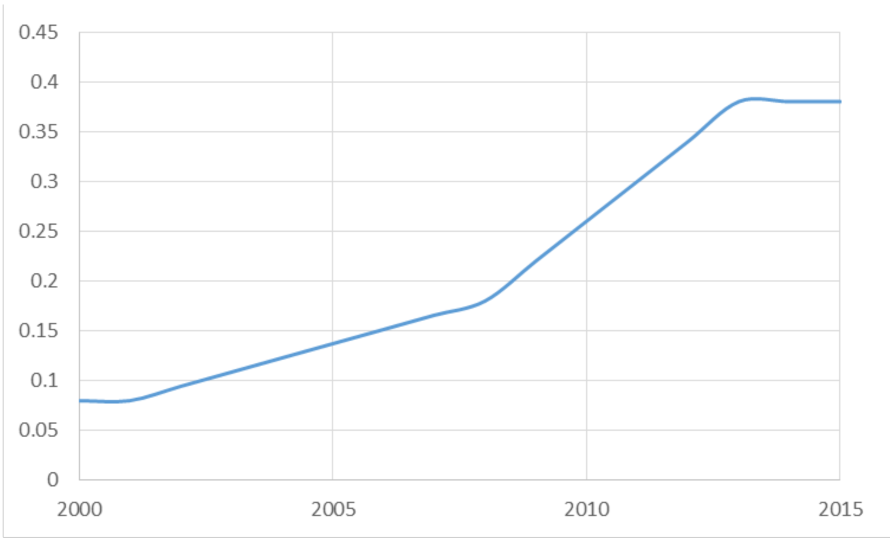

Figure 7. Correction factor applied to NEDC efficiencies. Plateaus pre-2000 and post-2015.

The future efficiency of ICVs is estimated based on a linear progression from the baseline to EU targets in 2021 (European Commission, 2015) (corrected for real world emissions). Further improvements are estimated based on a linear improvement to the IEA Blue scenario in 2035 (IEA, 2010) (as reported by Modaresi ***et al.*** (2014)) then following this scenario thereafter. The efficiency of non-ICVs (both past and future) was assumed to follow the IEA-BLUE scenario. The IEA scenarios appear to follow real life efficiencies more closely than historic NEDC values and so no correction is applied.

The fuel consumption scenario described above assumes a plateau in car mass. Reductions in car mass decrease the fuel requirement of vehicles. Information used here is taken from Wohlecker ***et al.*** (2007) as reported in Modaresi ***et al.*** (2014). The level of savings depends on the fuel and whether the vehicle's drivetrain is resized in-line with reductions in vehicle mass, in the current paper it is assumed that the drivetrain is resized. The data used for estimating the variation of vehicle efficiency with mass are shown in Table 8.

Table 8. Variation of vehicle efficiency with mass. Results shown include resizing of drivetrain.

Drivetrain	Petrol	Diesel	HEV-Petrol	HEV-Diesel	PHEV-Petrol	PHEV-Diesel	EV	FCV
% reduction in energy demand per 10% reduction in mass	6.8%	7.1%	5.7%	4.9%	5.7%	4.9%	3.1%	4.9%

6. EMISSIONS FACTORS FOR FUELS AND ELECTRICITY

Current and future emissions factors for fuels and electricity have been estimated from existing literature. For fuel combustion, emissions factors were estimated for the UK, based on UK literature. These factors can be found in Table 9. For electricity it is important to distinguish the UK grid used by electric vehicles in the UK and the average global grid used to manufacture materials worldwide. Both estimates of the UK and global electricity emissions factors have been based on the International Energy Agency New Policies Scenario (IEA, 2015b). This scenario was used directly for the global average grid, and the UK grid emissions were estimated by applying the same rate of improvement of the IEA New Policies Scenario to the UK electricity emissions factor in 2015. These values are shown in Table 10.

Table 9. Current emissions factors for fuel combustion.

**Fuel**	**Direct emissions [kg CO_{2eq}/GJ]**	**Indirect emissions [kg CO_{2eq}/GJ]**	**Total emissions [kg CO_{2eq}/GJ]**	**Comments**
Diesel	72.21	16.11	88.32	Direct and WTT factors for 2013, considering average biofuel blend. Source: HM Government (2015).
Petrol	68.12	13.75	81.87	Direct and WTT factors for 2013, considering average biofuel blend. Source: HM Government (2015).
Electricity (UK)	N/A	127.46	127.46	Factor for 2013. Includes all fossil fuel combustion emissions for the total electricity generated. Source: IEA (2015a).
Electricity (global average)	N/A	146.78	146.78	Factor for 2013. Includes all fossil fuel combustion emissions for the total electricity generated. Source: IEA (2015a).
Hydrogen	N/A	0.113	0.113	[kg CO2e/km] Considers 11888gCO2e/kg H2 produced from natural gas steam reforming. This is currently the most common production method but others may be considered. An average consumption of 0.9512 kgH2/100km was considered from Hyundai (2016).This can be highly variable. Source: Spath et al. (2001).

Table 10. Emissions factors for electricity generation.

Year	Emissions factor for the UK electricity grid [kg CO_{2eq}/GJ]	Emissions factor for an average global electricity grid [kg CO_{2eq}/GJ]
2015	125.5	144.5
2016	124.5	143.4
2017	123.6	142.3
2018	122.6	141.2
2019	121.6	140.1
2020	120.7	139.0
2021	118.7	136.7
2022	116.8	134.5
2023	114.8	132.2
2024	112.9	130.0
2025	110.9	127.7
2026	109.3	125.9
2027	107.7	124.0
2028	106.1	122.2
2029	104.5	120.4
2030	102.9	118.5
2031	101.7	117.1
2032	100.5	115.8
2033	99.3	114.4
2034	98.1	113.0
2035	96.9	111.6
2036	95.9	110.5
2037	95.0	109.4
2038	94.0	108.3
2039	93.1	107.2
2040	92.1	106.1
2041	90.7	104.5
2042	89.7	103.3
2043	88.6	102.0
2044	87.5	100.8
2045	86.4	99.5
2046	85.3	98.3
2047	84.2	97.0
2048	83.2	95.8
2049	82.1	94.5
2050	81.0	93.3

7. DISTANCE TRAVELLED BY CAR AGE

The number of cars in the fleet obtained as described in section 1 is not enough to define its capacity to deliver service. This could be either a uniform distribution, assuming that all cars in service would travel on average the same distances each year, or a different distribution that takes car vintage into account. We have opted by this second option, since Serrenho ***et al.*** (2016) verified that older cars tend to travel less than young cars, so the influence of the composition of the fleet in their ability to provide transportation service should be considered.

The National Travel Survey (Department for Transport, 2016a) provides historical data for car use, and enabled the estimation a linear regression model to characterise the average distance travelled by a car of age i (D_i in thousand km)as a function of the overall distance travelled for the entire fleet ($\bar{D}$ in thousand km). This model is defined as

$$D = k\beta_i\,\bar{D}.$$

Applying linear regression to the historical values of car use of the National Travel Survey between 2002 and 2014, the coefficients β_i have been estimated for each car age cohort. Table 11 shows the values obtained. k is a constant required to scale this relationship for all ages of cars in future time periods and will vary due to the overall service demand and the age profile of the stock in a given year.

Table 11. Regression coefficients estimated for the linear regression model of car age and distance travelled.

Car age cohort	β_i
< 1 a	1.301
1 a	1.301
2 a	1.203
3 a	1.142
4 a	1.059
5 a	1.033
6 a	0.966
7 a	0.931
8 - 9 a	0.884
10 - 12 a	0.804
13 - 17 a	0.690
18+ a	0.396

This historical relation between car age and distance travelled was used for the period of this analysis (2015—2050).

8. ANNUAL COMPOSITION OF THE CAR FLEET

This section shows the annual composition of the car fleet for the following scenarios: reference, new sales only comprised of EVs and PHEVs with steel-intensive material composition, and new sales only comprised of EVs and PHEVs, steel-intensive material composition and +20% of occupancy and intensity of use.

8.1. Reference scenario

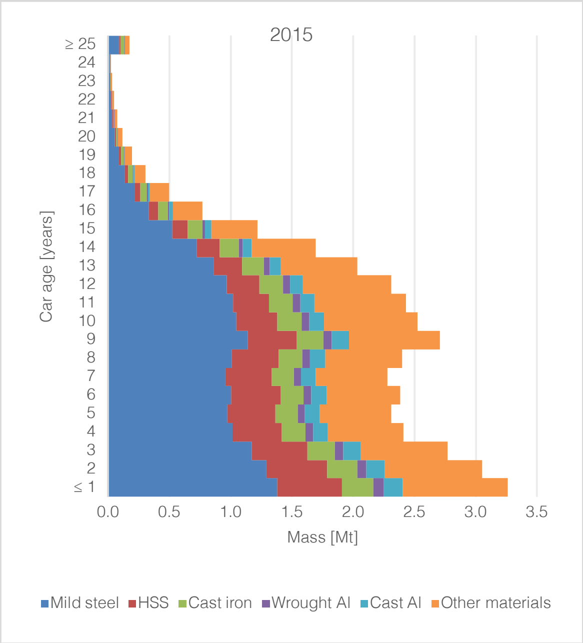

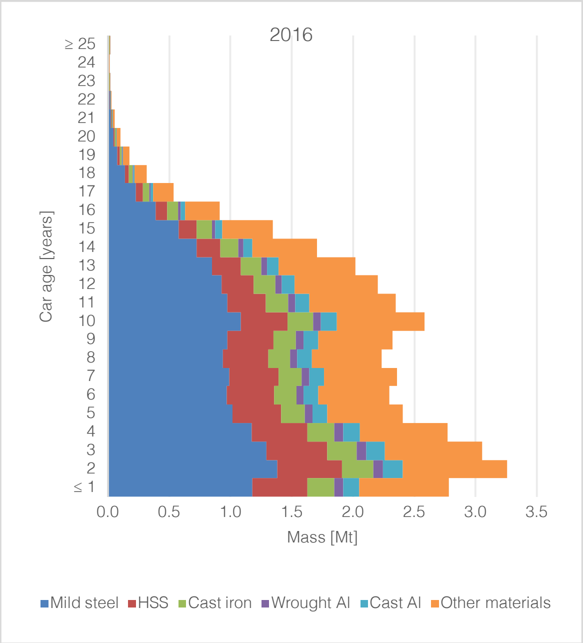

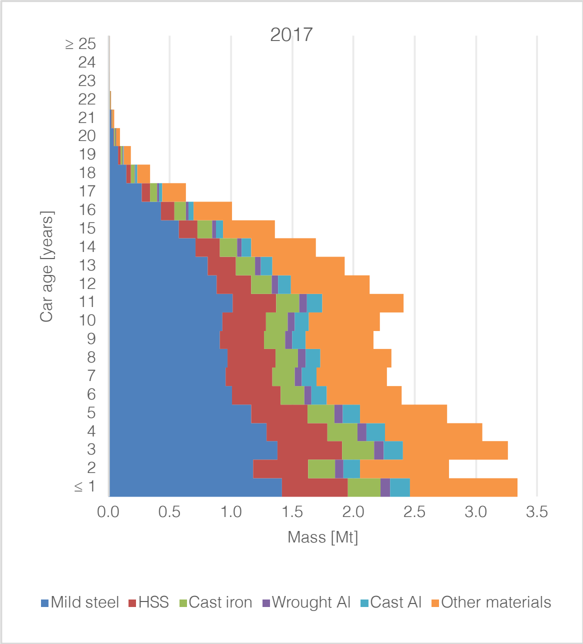

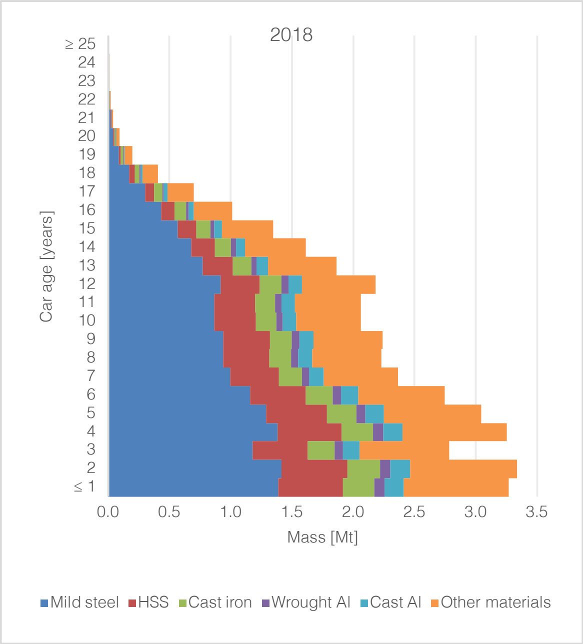

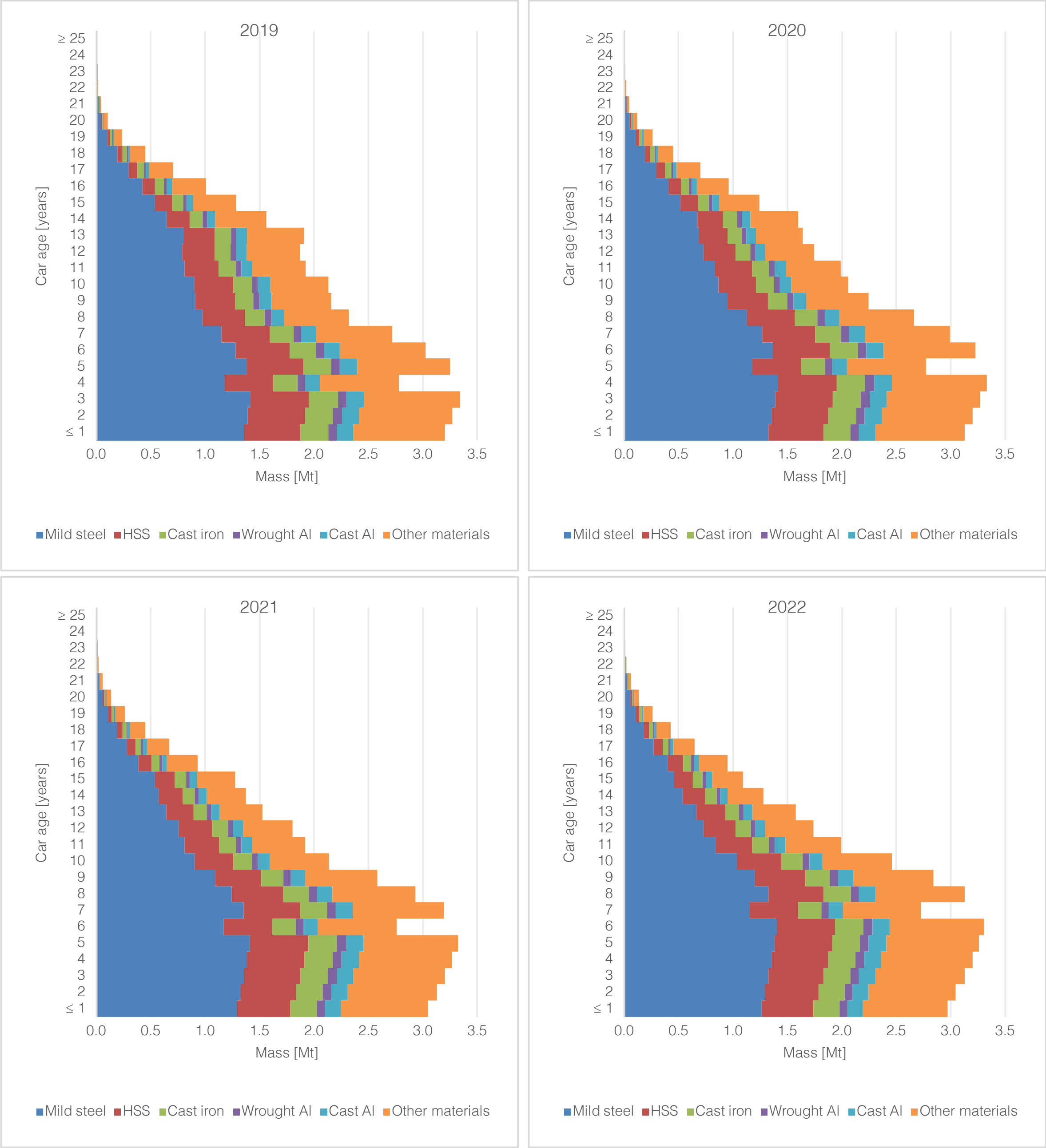
2019
2020
2021
2022
Car age [years]
≥ 25
24
23
22
21
20
19
18
17
16
15
14
13
12
11
10
9
8
7
6
5
4
3
2
≤ 1
0.0
0.5
1.0
1.5
2.0
2.5
3.0
3.5
Mass [Mt]
Mild steel
HSS
Cast iron
Wrought Al
Cast Al
Other materials

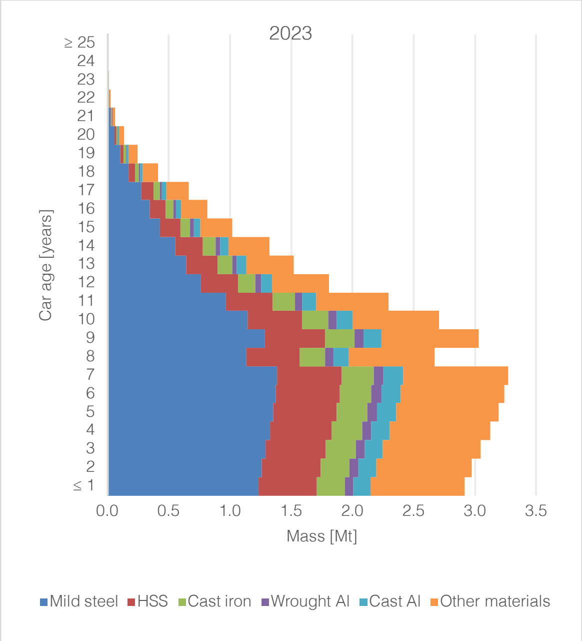
2023
Car age [years]
≥ 25
24
23
22
21
20
19
18
17
16
15
14
13
12
11
10
9
8
7
6
5
4
3
2
≤ 1
0.0
0.5
1.0
1.5
2.0
2.5
3.0
3.5
Mass [Mt]
Mild steel
HSS
Cast iron
Wrought Al
Cast Al
Other materials

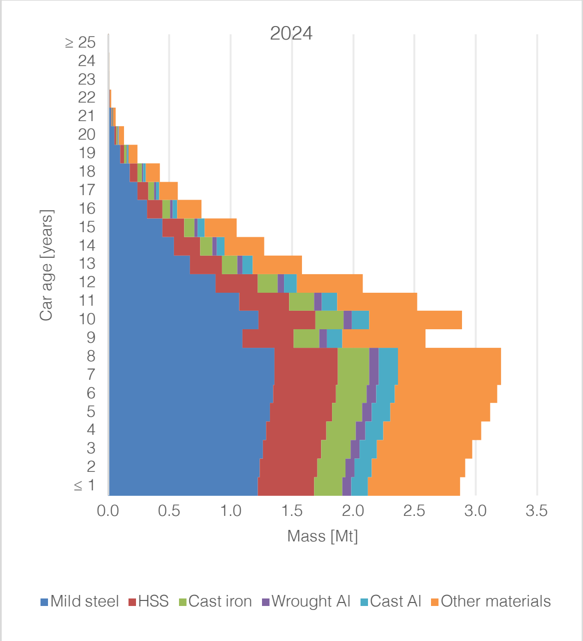
2024
Car age [years]
≥ 25
24
23
22
21
20
19
18
17
16
15
14
13
12
11
10
9
8
7
6
5
4
3
2
≤ 1
0.0
0.5
1.0
1.5
2.0
2.5
3.0
3.5
Mass [Mt]
Mild steel
HSS
Cast iron
Wrought Al
Cast Al
Other materials

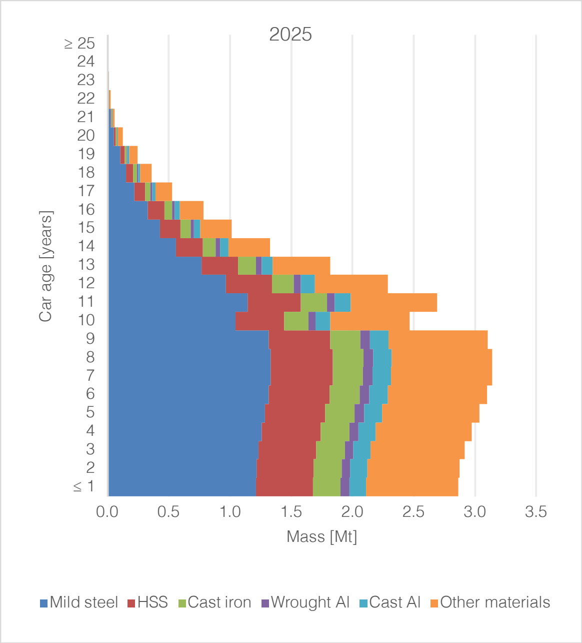
2025
Car age [years]
≥ 25
24
23
22
21
20
19
18
17
16
15
14
13
12
11
10
9
8
7
6
5
4
3
2
≤ 1
0.0
0.5
1.0
1.5
2.0
2.5
3.0
3.5
Mass [Mt]
Mild steel
HSS
Cast iron
Wrought Al
Cast Al
Other materials

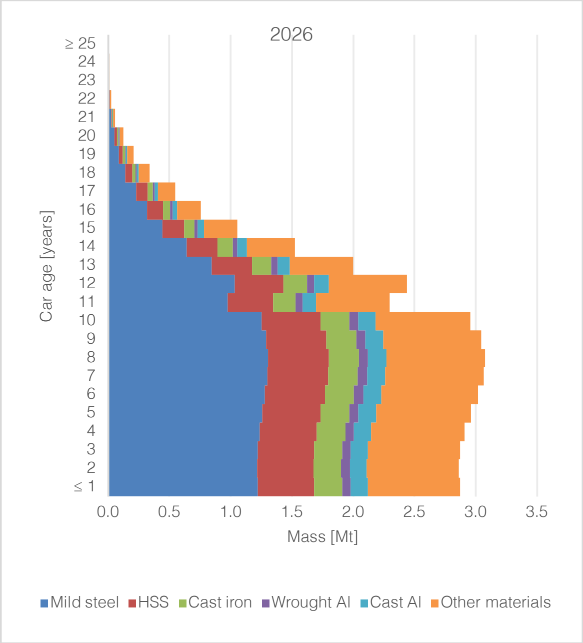
2026
Car age [years]
≥ 25
24
23
22
21
20
19
18
17
16
15
14
13
12
11
10
9
8
7
6
5
4
3
2
≤ 1
0.0
0.5
1.0
1.5
2.0
2.5
3.0
3.5
Mass [Mt]
Mild steel
HSS
Cast iron
Wrought Al
Cast Al
Other materials

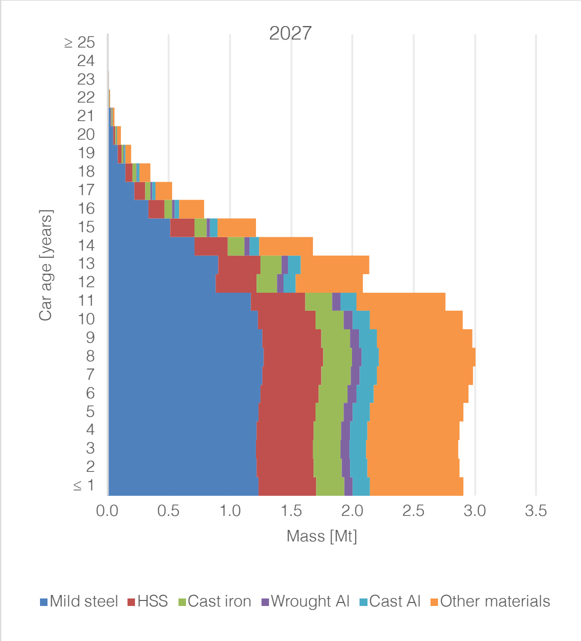
2027
≥ 25
24
23
22
21
20
19
18
17
16
15
14
13
12
11
10
9
8
7
6
5
4
3
2
≤ 1
Car age [years]
0.0 0.5 1.0 1.5 2.0 2.5 3.0 3.5
Mass [Mt]
Mild steel HSS Cast iron Wrought Al Cast Al Other materials

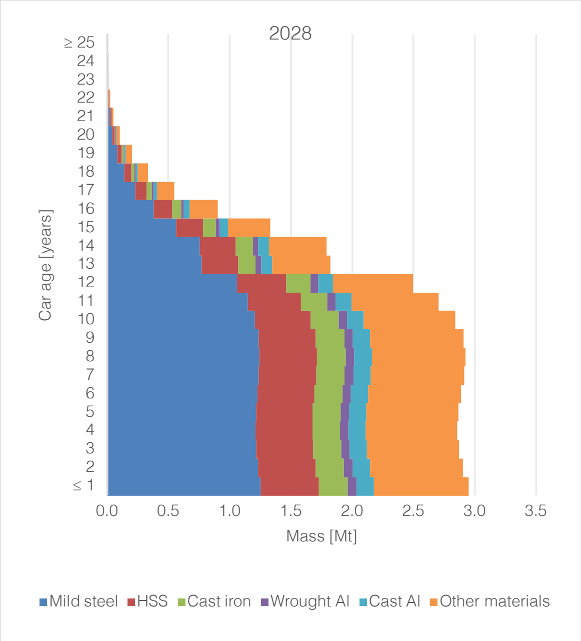
2028
≥ 25
24
23
22
21
20
19
18
17
16
15
14
13
12
11
10
9
8
7
6
5
4
3
2
≤ 1
Car age [years]
0.0 0.5 1.0 1.5 2.0 2.5 3.0 3.5
Mass [Mt]
Mild steel HSS Cast iron Wrought Al Cast Al Other materials

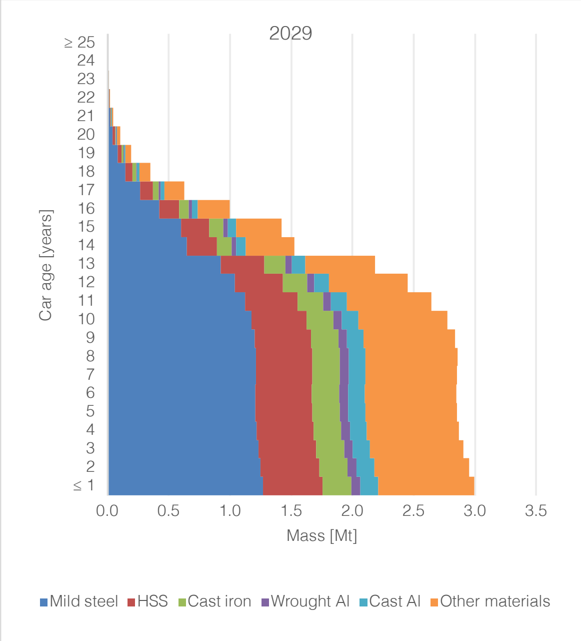
2029
≥ 25
24
23
22
21
20
19
18
17
16
15
14
13
12
11
10
9
8
7
6
5
4
3
2
≤ 1
Car age [years]
0.0 0.5 1.0 1.5 2.0 2.5 3.0 3.5
Mass [Mt]
Mild steel HSS Cast iron Wrought Al Cast Al Other materials

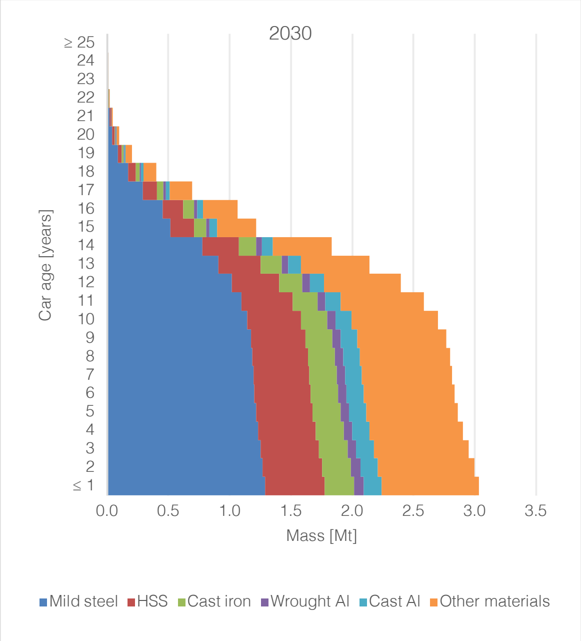
2030
≥ 25
24
23
22
21
20
19
18
17
16
15
14
13
12
11
10
9
8
7
6
5
4
3
2
≤ 1
Car age [years]
0.0 0.5 1.0 1.5 2.0 2.5 3.0 3.5
Mass [Mt]
Mild steel HSS Cast iron Wrought Al Cast Al Other materials

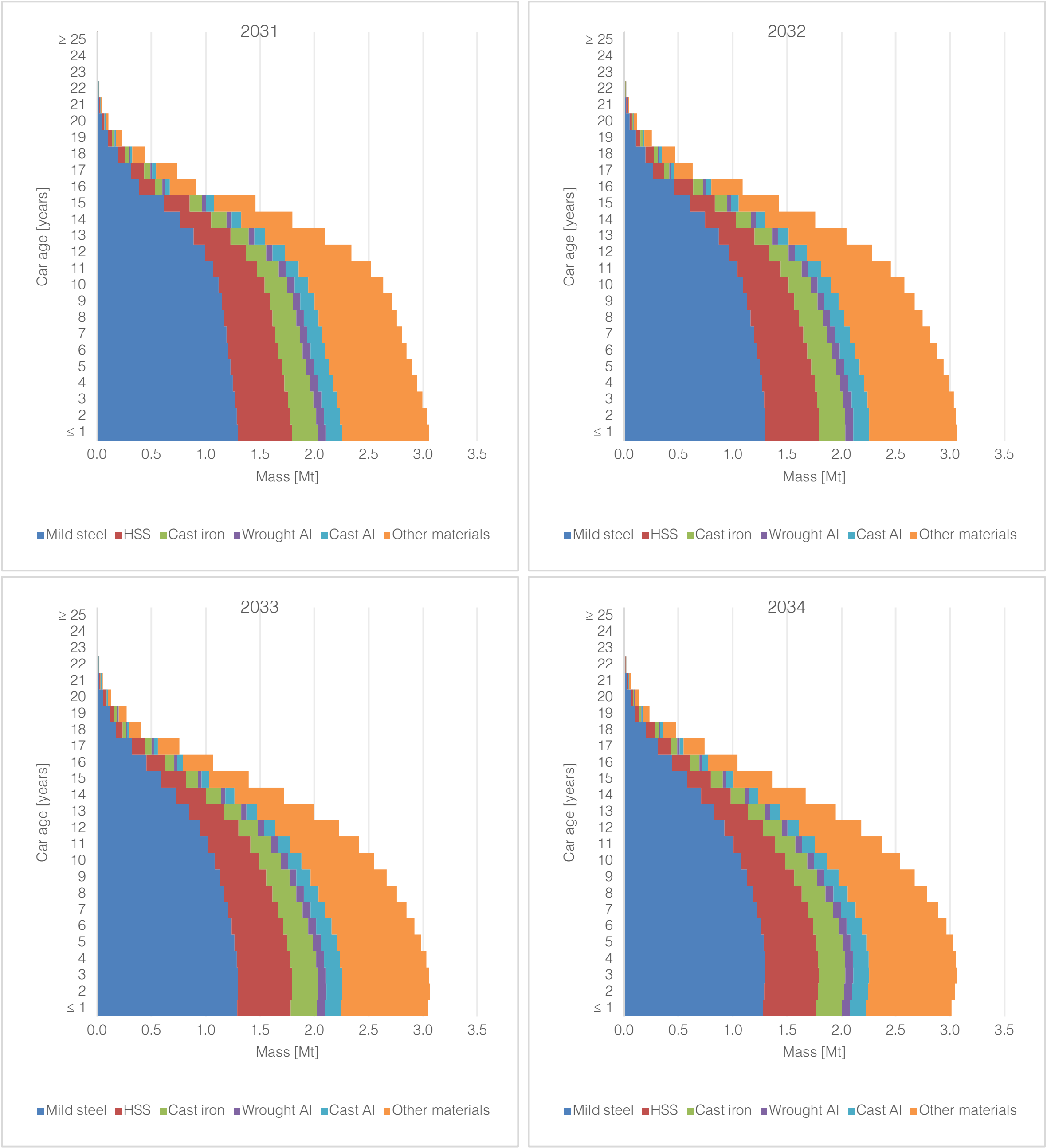
2031
2032
2033
2034
Car age [years]
≥ 25
≤ 1
Mass [Mt]
Mild steel
HSS
Cast iron
Wrought Al
Cast Al
Other materials

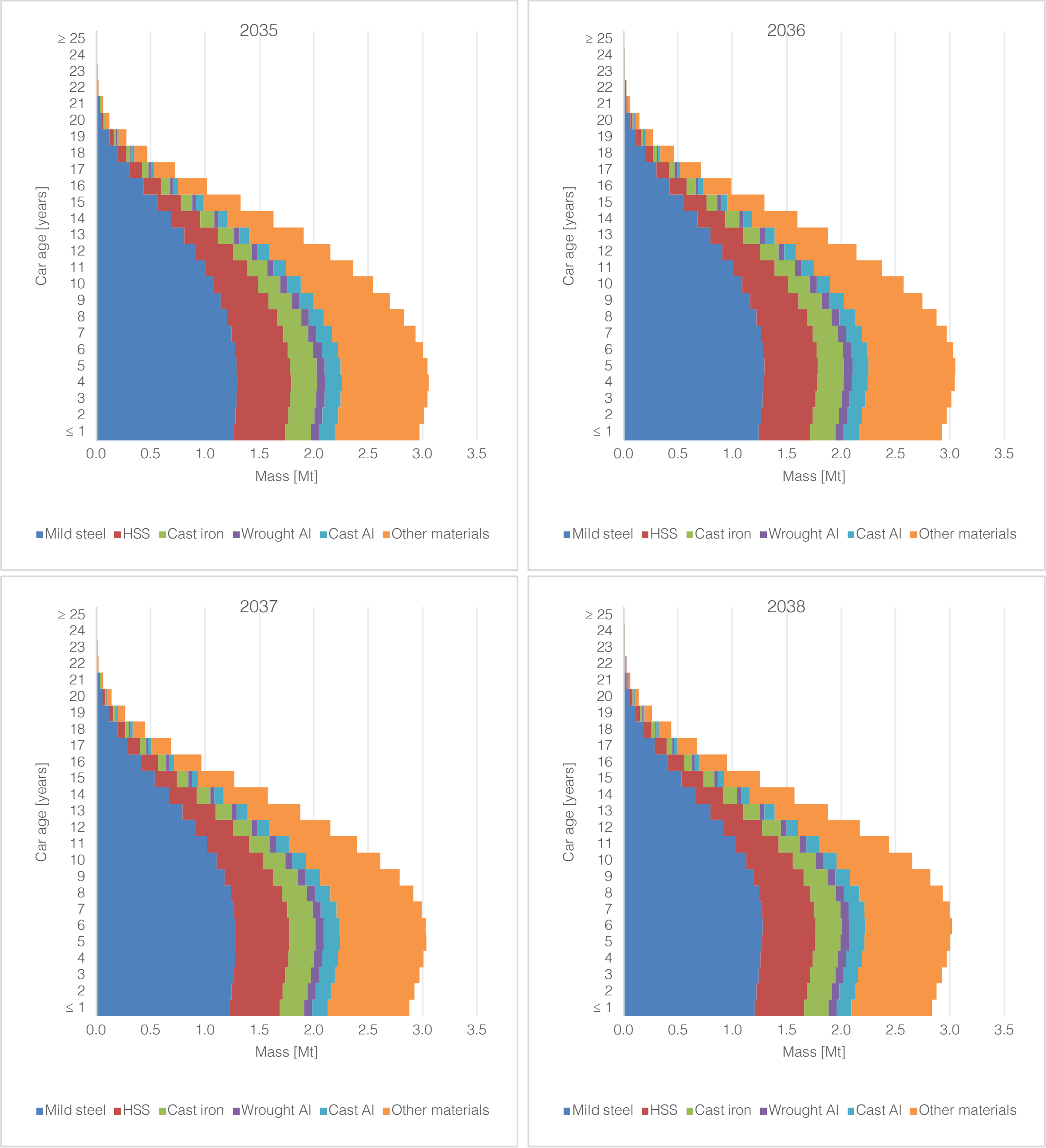
2035
2036
2037
2038
Car age [years]
≥ 25
24
23
22
21
20
19
18
17
16
15
14
13
12
11
10
9
8
7
6
5
4
3
2
≤ 1
0.0
0.5
1.0
1.5
2.0
2.5
3.0
3.5
Mass [Mt]
Mild steel
HSS
Cast iron
Wrought Al
Cast Al
Other materials

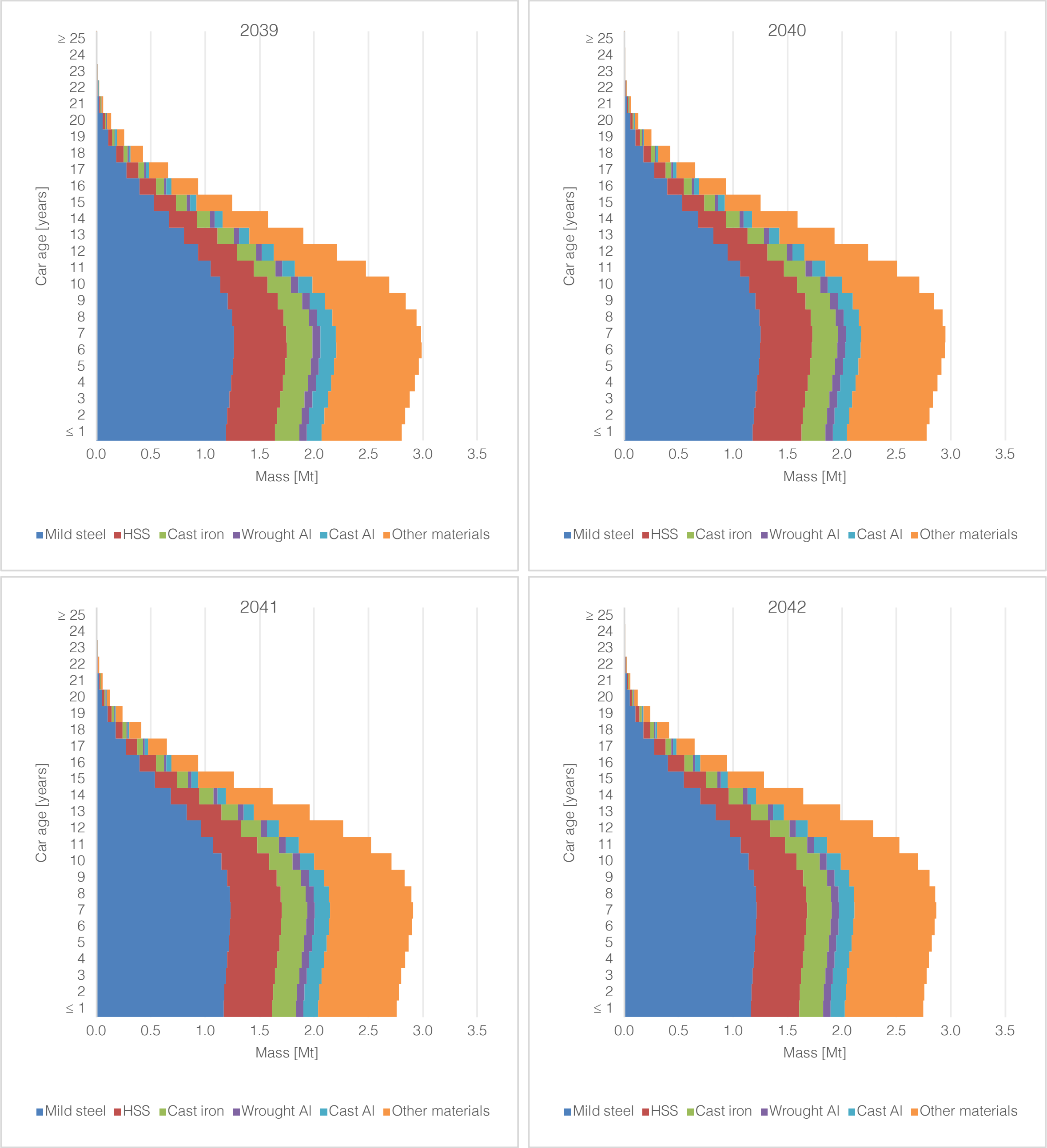
2039
2040
2041
2042
Car age [years]
≥ 25
24
23
22
21
20
19
18
17
16
15
14
13
12
11
10
9
8
7
6
5
4
3
2
≤ 1
0.0
0.5
1.0
1.5
2.0
2.5
3.0
3.5
Mass [Mt]
Mild steel
HSS
Cast iron
Wrought Al
Cast Al
Other materials

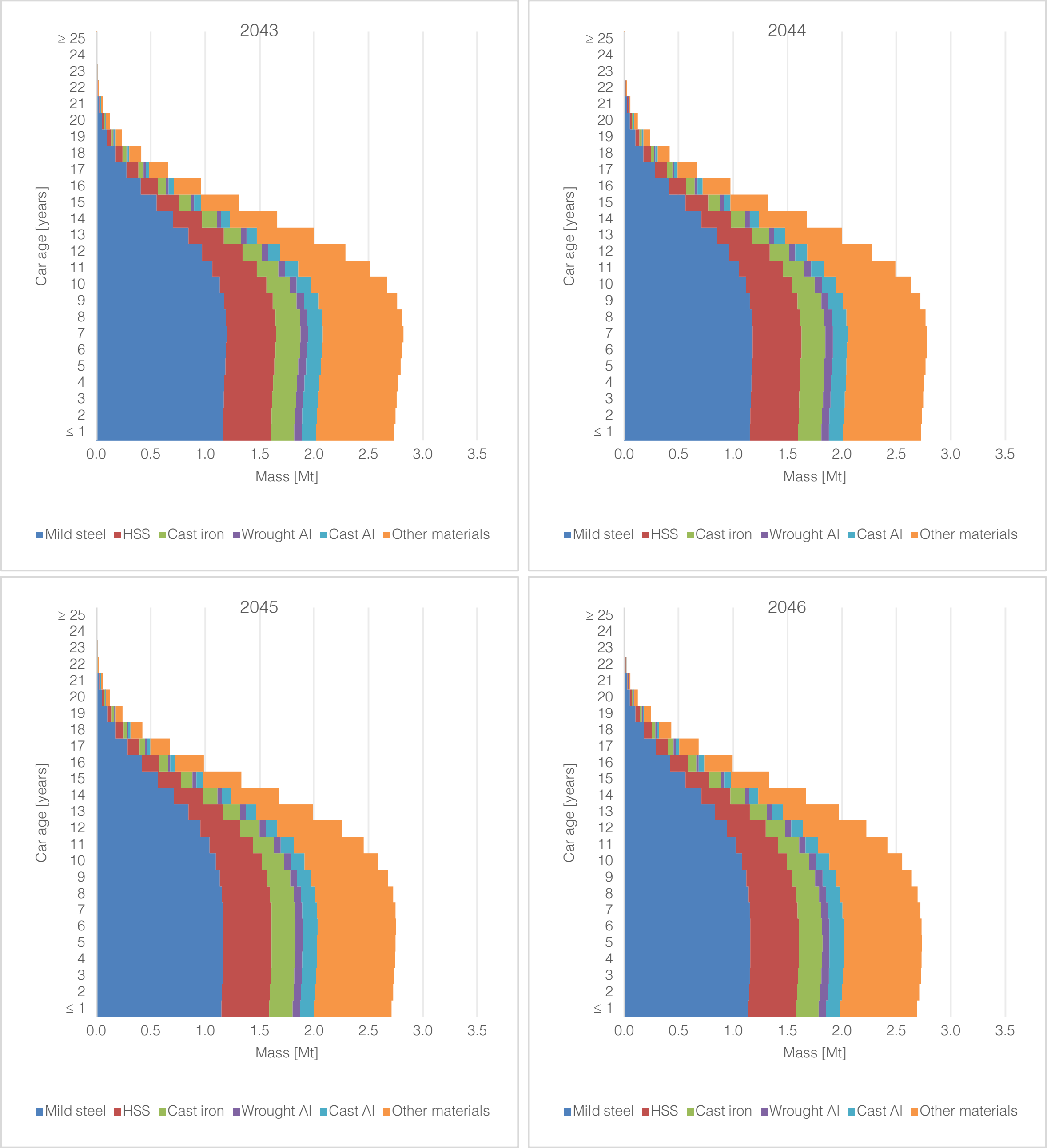
2043
2044
2045
2046
Car age [years]
≥ 25
24
23
22
21
20
19
18
17
16
15
14
13
12
11
10
9
8
7
6
5
4
3
2
≤ 1
0.0
0.5
1.0
1.5
2.0
2.5
3.0
3.5
Mass [Mt]
Mild steel
HSS
Cast iron
Wrought Al
Cast Al
Other materials

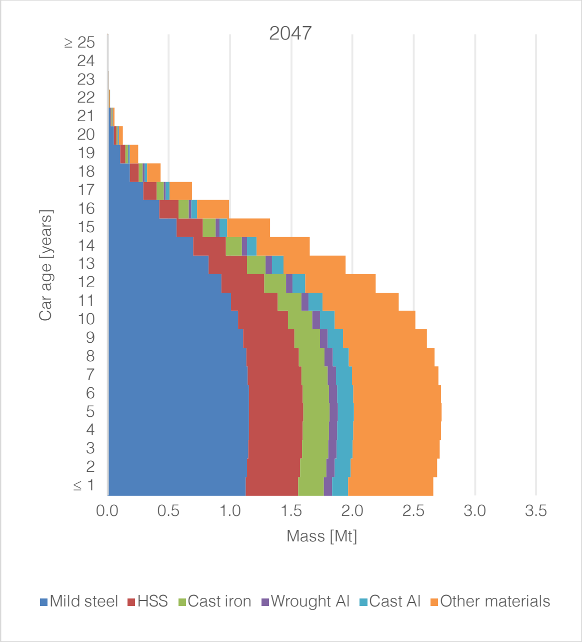
2047
Car age [years]
≥ 25
24
23
22
21
20
19
18
17
16
15
14
13
12
11
10
9
8
7
6
5
4
3
2
≤ 1
0.0
0.5
1.0
1.5
2.0
2.5
3.0
3.5
Mass [Mt]
Mild steel
HSS
Cast iron
Wrought Al
Cast Al
Other materials

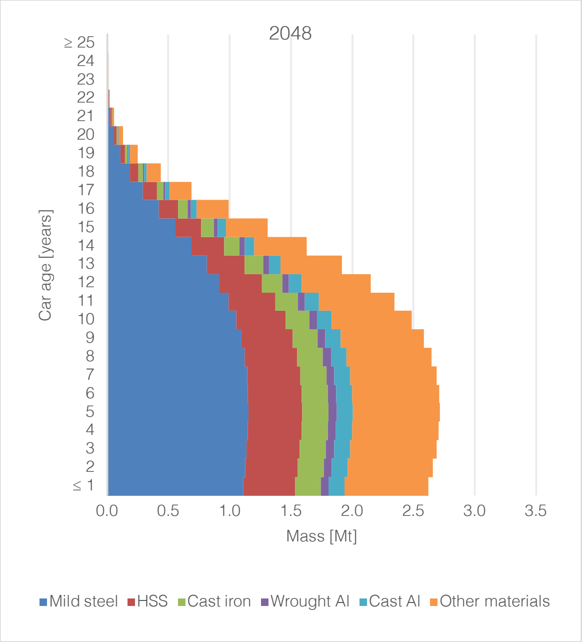
2048
Car age [years]
≥ 25
24
23
22
21
20
19
18
17
16
15
14
13
12
11
10
9
8
7
6
5
4
3
2
≤ 1
0.0
0.5
1.0
1.5
2.0
2.5
3.0
3.5
Mass [Mt]
Mild steel
HSS
Cast iron
Wrought Al
Cast Al
Other materials

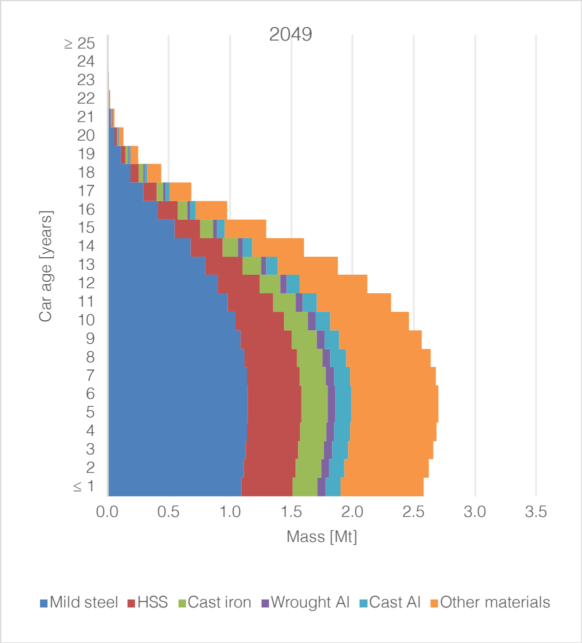
2049
Car age [years]
≥ 25
24
23
22
21
20
19
18
17
16
15
14
13
12
11
10
9
8
7
6
5
4
3
2
≤ 1
0.0
0.5
1.0
1.5
2.0
2.5
3.0
3.5
Mass [Mt]
Mild steel
HSS
Cast iron
Wrought Al
Cast Al
Other materials

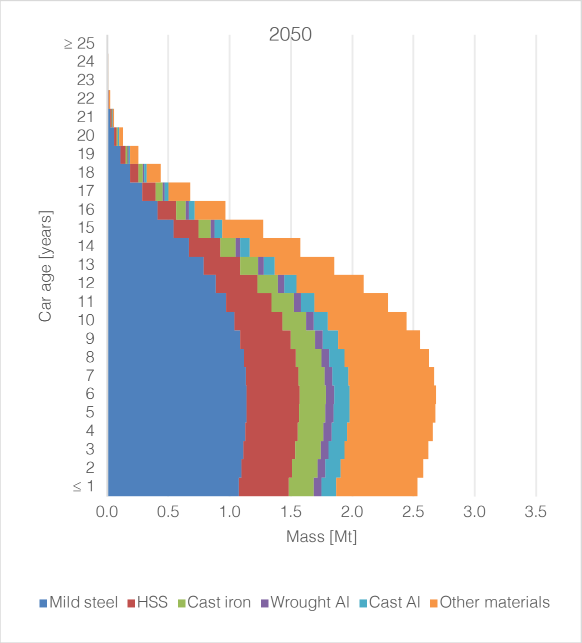
2050
Car age [years]
≥ 25
24
23
22
21
20
19
18
17
16
15
14
13
12
11
10
9
8
7
6
5
4
3
2
≤ 1
0.0
0.5
1.0
1.5
2.0
2.5
3.0
3.5
Mass [Mt]
Mild steel
HSS
Cast iron
Wrought Al
Cast Al
Other materials

8.2. New sales only comprised of EVs and PHEVs with steel-intensive material composition

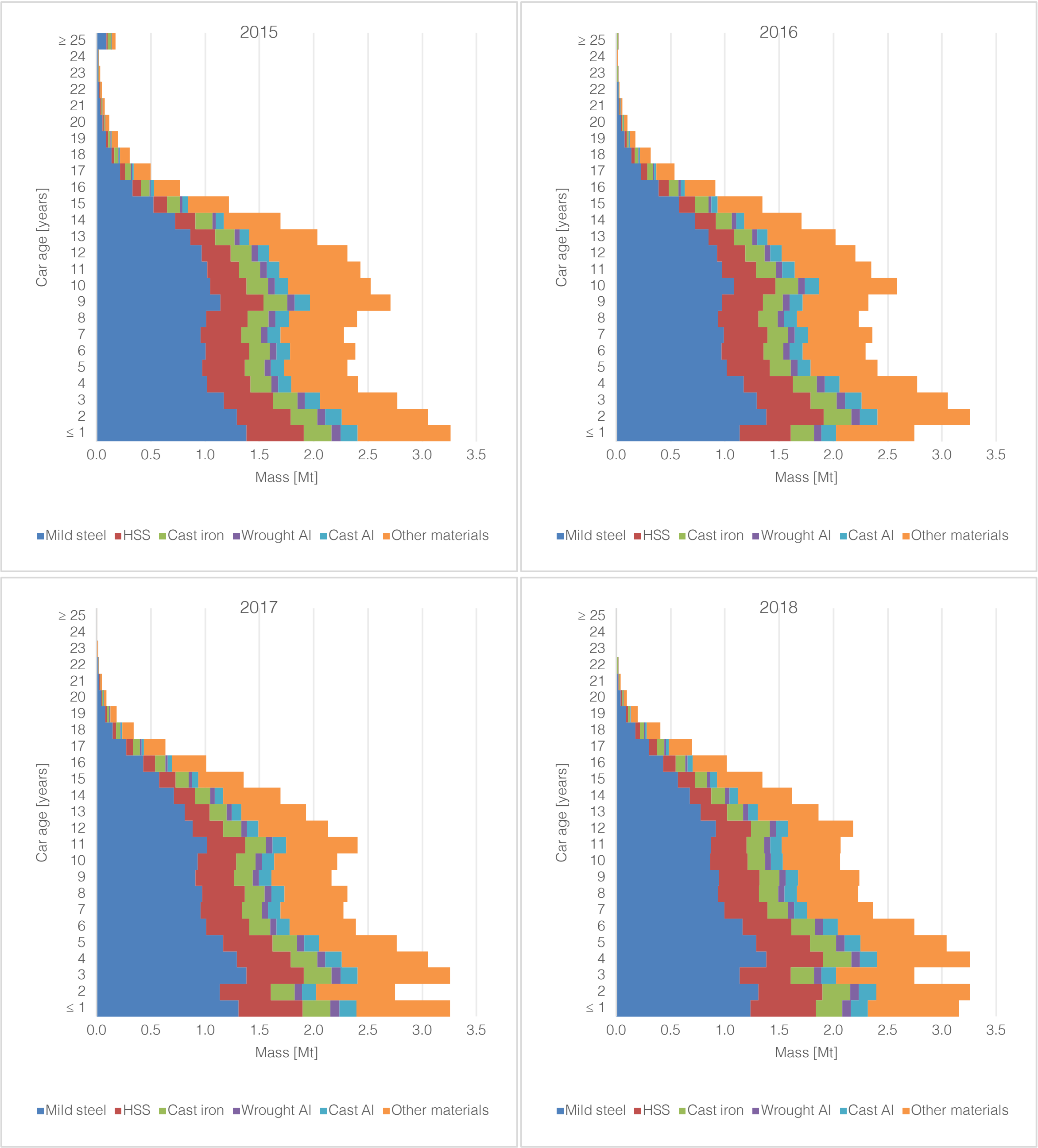

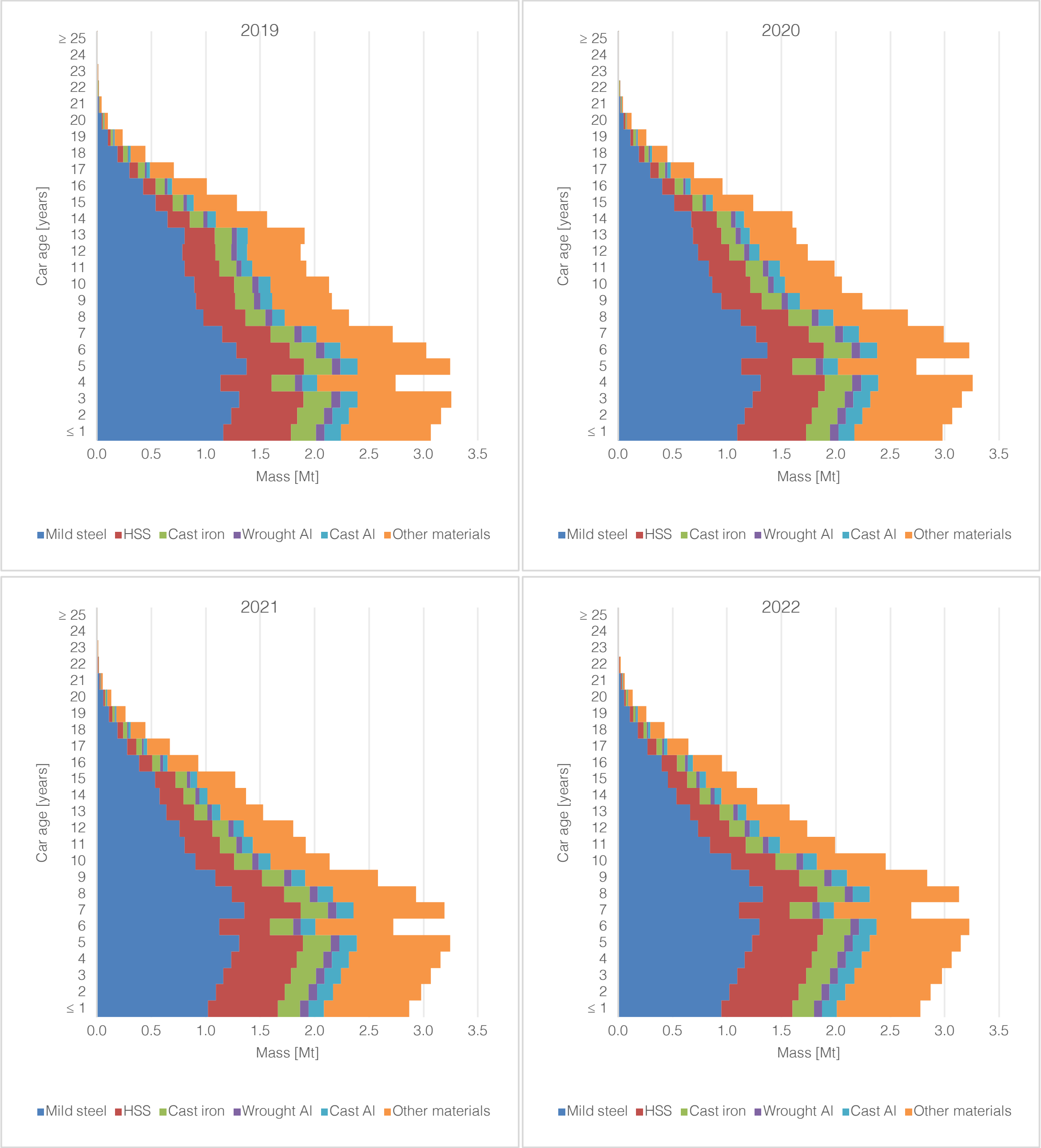
2019
2020
2021
2022
Car age [years]
≥ 25
24
23
22
21
20
19
18
17
16
15
14
13
12
11
10
9
8
7
6
5
4
3
2
≤ 1
0.0 0.5 1.0 1.5 2.0 2.5 3.0 3.5
Mass [Mt]
Mild steel
HSS
Cast iron
Wrought Al
Cast Al
Other materials

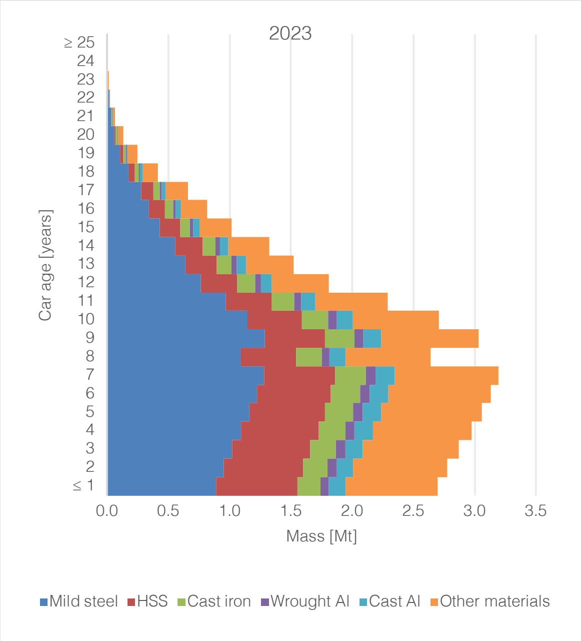
2023
≥ 25
24
23
22
21
20
19
18
17
16
15
14
13
12
11
10
9
8
7
6
5
4
3
2
≤ 1
Car age [years]
0.0 0.5 1.0 1.5 2.0 2.5 3.0 3.5
Mass [Mt]
Mild steel
HSS
Cast iron
Wrought Al
Cast Al
Other materials

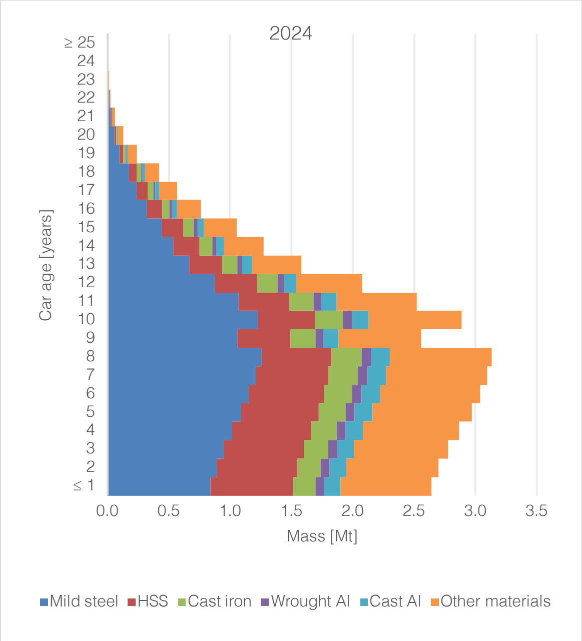
2024
≥ 25
24
23
22
21
20
19
18
17
16
15
14
13
12
11
10
9
8
7
6
5
4
3
2
≤ 1
Car age [years]
0.0 0.5 1.0 1.5 2.0 2.5 3.0 3.5
Mass [Mt]
Mild steel
HSS
Cast iron
Wrought Al
Cast Al
Other materials

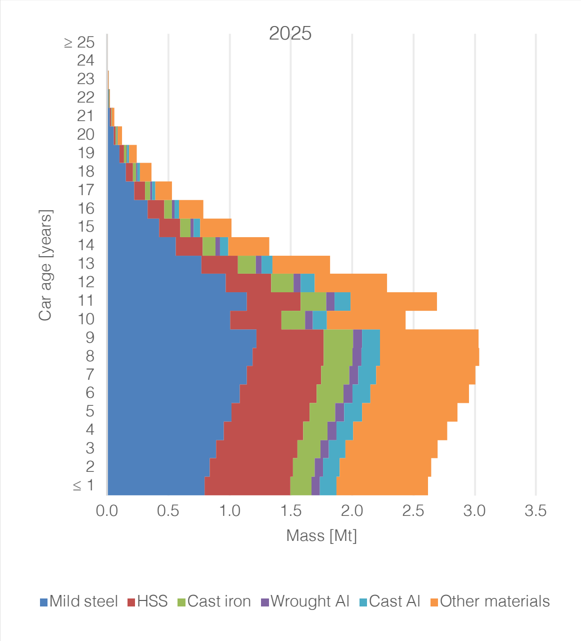
2025
≥ 25
24
23
22
21
20
19
18
17
16
15
14
13
12
11
10
9
8
7
6
5
4
3
2
≤ 1
Car age [years]
0.0 0.5 1.0 1.5 2.0 2.5 3.0 3.5
Mass [Mt]
Mild steel
HSS
Cast iron
Wrought Al
Cast Al
Other materials

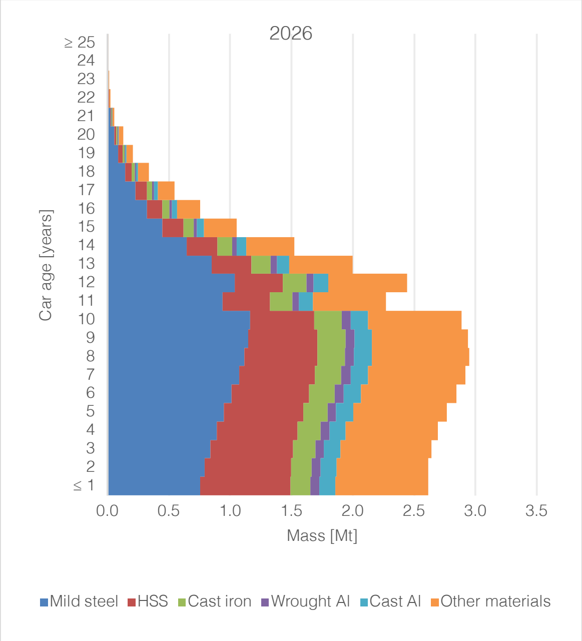
2026
≥ 25
24
23
22
21
20
19
18
17
16
15
14
13
12
11
10
9
8
7
6
5
4
3
2
≤ 1
Car age [years]
0.0 0.5 1.0 1.5 2.0 2.5 3.0 3.5
Mass [Mt]
Mild steel
HSS
Cast iron
Wrought Al
Cast Al
Other materials

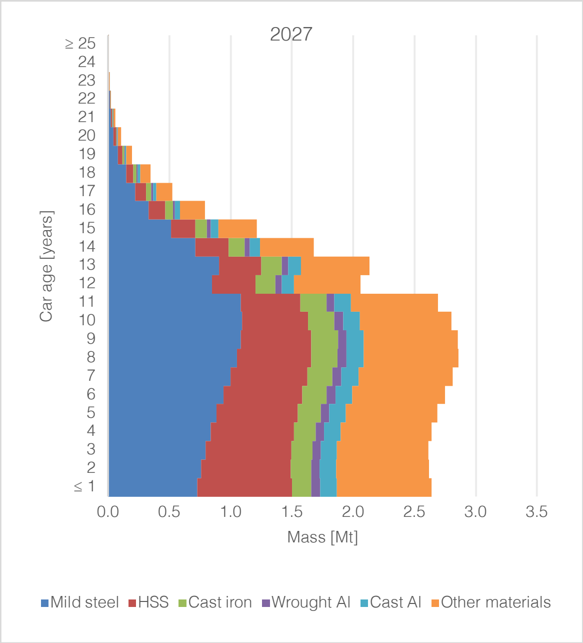
2027
Car age [years]
≥ 25
24
23
22
21
20
19
18
17
16
15
14
13
12
11
10
9
8
7
6
5
4
3
2
≤ 1
0.0 0.5 1.0 1.5 2.0 2.5 3.0 3.5
Mass [Mt]
Mild steel
HSS
Cast iron
Wrought Al
Cast Al
Other materials

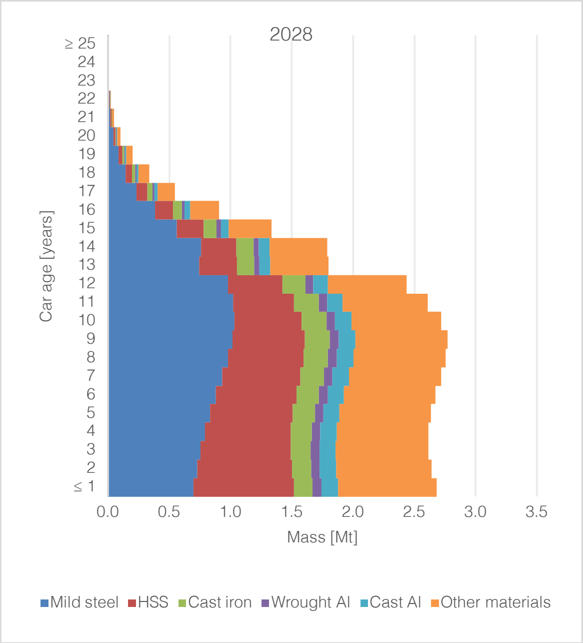
2028
Car age [years]
≥ 25
24
23
22
21
20
19
18
17
16
15
14
13
12
11
10
9
8
7
6
5
4
3
2
≤ 1
0.0 0.5 1.0 1.5 2.0 2.5 3.0 3.5
Mass [Mt]
Mild steel
HSS
Cast iron
Wrought Al
Cast Al
Other materials

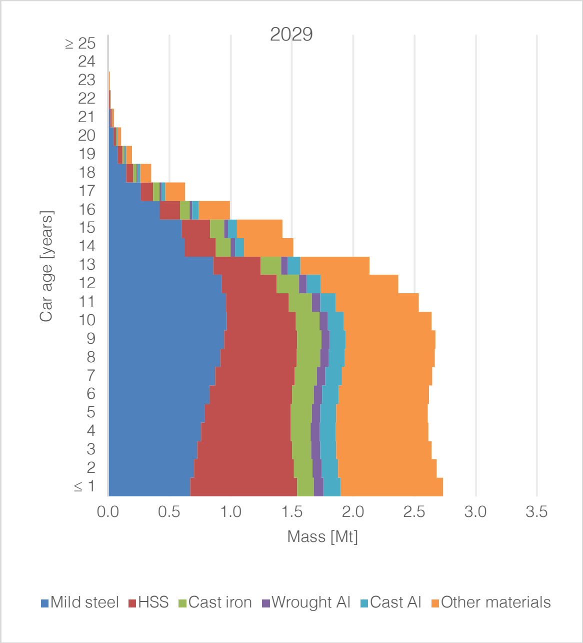
2029
Car age [years]
≥ 25
24
23
22
21
20
19
18
17
16
15
14
13
12
11
10
9
8
7
6
5
4
3
2
≤ 1
0.0 0.5 1.0 1.5 2.0 2.5 3.0 3.5
Mass [Mt]
Mild steel
HSS
Cast iron
Wrought Al
Cast Al
Other materials

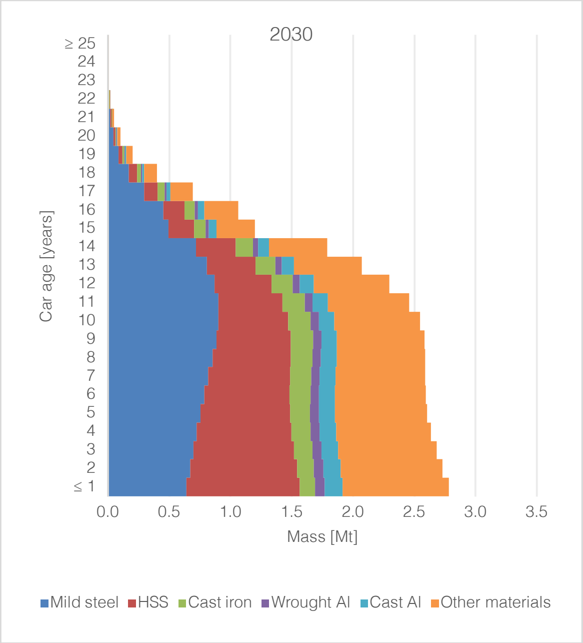
2030
Car age [years]
≥ 25
24
23
22
21
20
19
18
17
16
15
14
13
12
11
10
9
8
7
6
5
4
3
2
≤ 1
0.0 0.5 1.0 1.5 2.0 2.5 3.0 3.5
Mass [Mt]
Mild steel
HSS
Cast iron
Wrought Al
Cast Al
Other materials

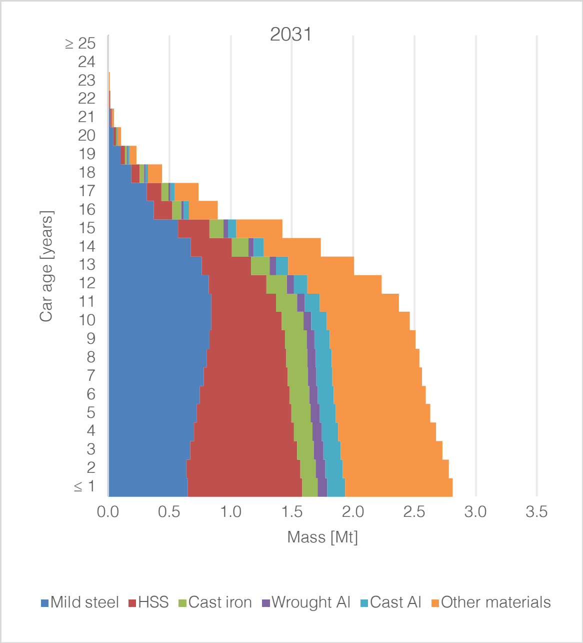
2031
Car age [years]
≥ 25
24
23
22
21
20
19
18
17
16
15
14
13
12
11
10
9
8
7
6
5
4
3
2
≤ 1
0.0 0.5 1.0 1.5 2.0 2.5 3.0 3.5
Mass [Mt]
Mild steel
HSS
Cast iron
Wrought Al
Cast Al
Other materials

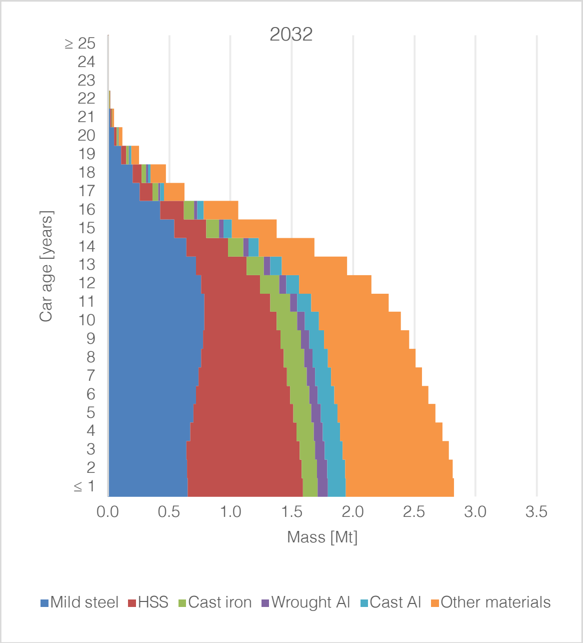
2032
Car age [years]
≥ 25
24
23
22
21
20
19
18
17
16
15
14
13
12
11
10
9
8
7
6
5
4
3
2
≤ 1
0.0 0.5 1.0 1.5 2.0 2.5 3.0 3.5
Mass [Mt]
Mild steel
HSS
Cast iron
Wrought Al
Cast Al
Other materials

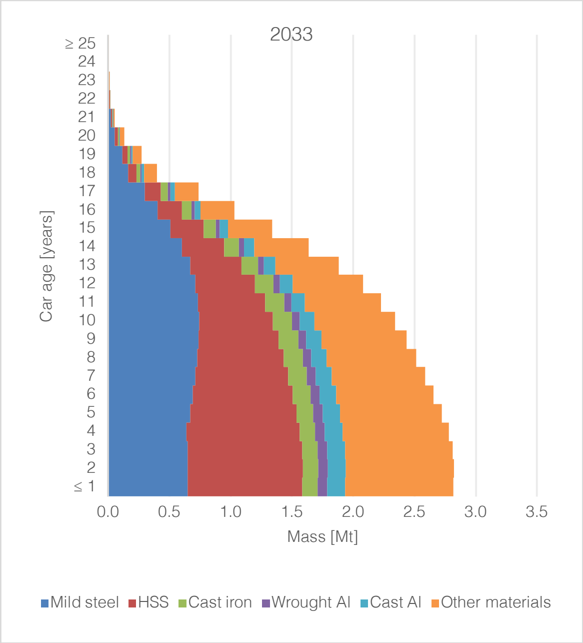
2033
Car age [years]
≥ 25
24
23
22
21
20
19
18
17
16
15
14
13
12
11
10
9
8
7
6
5
4
3
2
≤ 1
0.0 0.5 1.0 1.5 2.0 2.5 3.0 3.5
Mass [Mt]
Mild steel
HSS
Cast iron
Wrought Al
Cast Al
Other materials

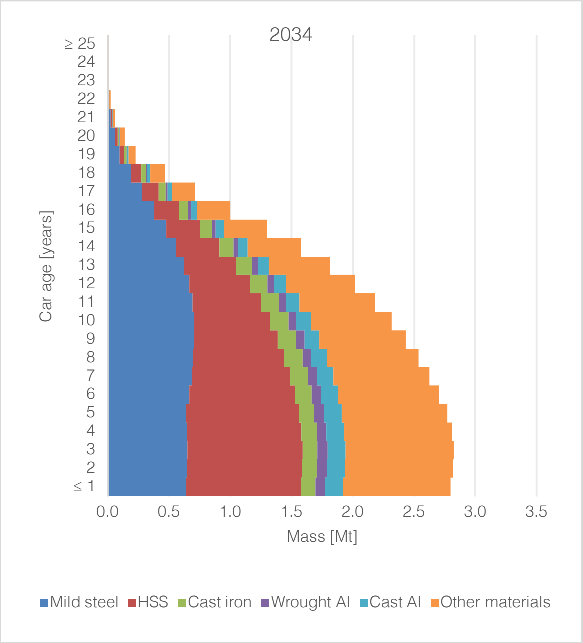
2034
Car age [years]
≥ 25
24
23
22
21
20
19
18
17
16
15
14
13
12
11
10
9
8
7
6
5
4
3
2
≤ 1
0.0 0.5 1.0 1.5 2.0 2.5 3.0 3.5
Mass [Mt]
Mild steel
HSS
Cast iron
Wrought Al
Cast Al
Other materials

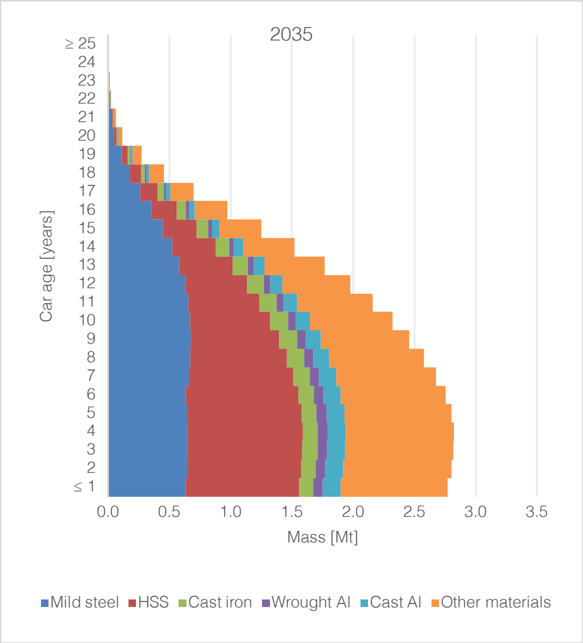
2035
Car age [years]
≥ 25
24
23
22
21
20
19
18
17
16
15
14
13
12
11
10
9
8
7
6
5
4
3
2
≤ 1
0.0 0.5 1.0 1.5 2.0 2.5 3.0 3.5
Mass [Mt]
Mild steel
HSS
Cast iron
Wrought Al
Cast Al
Other materials

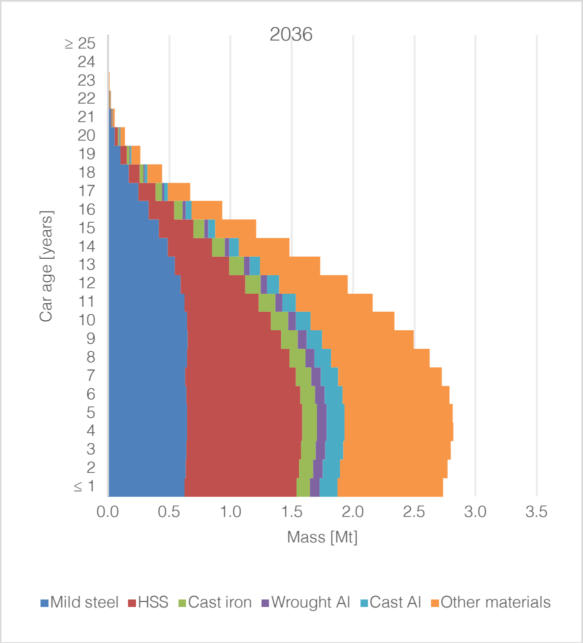
2036
Car age [years]
≥ 25
24
23
22
21
20
19
18
17
16
15
14
13
12
11
10
9
8
7
6
5
4
3
2
≤ 1
0.0 0.5 1.0 1.5 2.0 2.5 3.0 3.5
Mass [Mt]
Mild steel
HSS
Cast iron
Wrought Al
Cast Al
Other materials

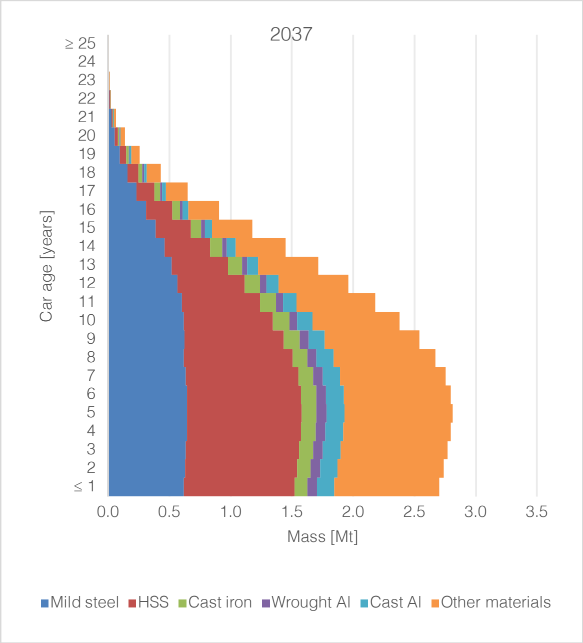
2037
Car age [years]
≥ 25
24
23
22
21
20
19
18
17
16
15
14
13
12
11
10
9
8
7
6
5
4
3
2
≤ 1
0.0 0.5 1.0 1.5 2.0 2.5 3.0 3.5
Mass [Mt]
Mild steel
HSS
Cast iron
Wrought Al
Cast Al
Other materials

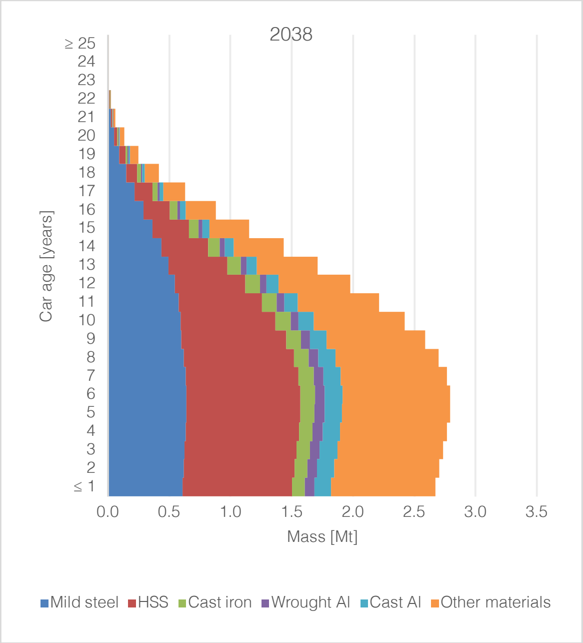
2038
Car age [years]
≥ 25
24
23
22
21
20
19
18
17
16
15
14
13
12
11
10
9
8
7
6
5
4
3
2
≤ 1
0.0 0.5 1.0 1.5 2.0 2.5 3.0 3.5
Mass [Mt]
Mild steel
HSS
Cast iron
Wrought Al
Cast Al
Other materials

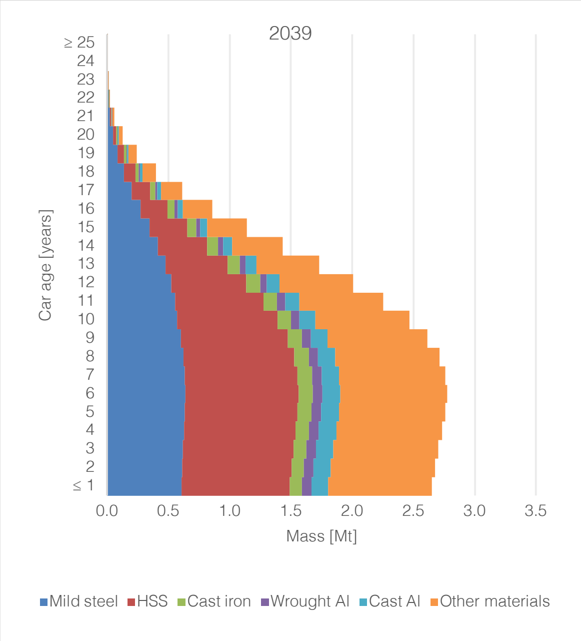
2039
Car age [years]
≥ 25
24
23
22
21
20
19
18
17
16
15
14
13
12
11
10
9
8
7
6
5
4
3
2
≤ 1
0.0
0.5
1.0
1.5
2.0
2.5
3.0
3.5
Mass [Mt]
Mild steel
HSS
Cast iron
Wrought Al
Cast Al
Other materials

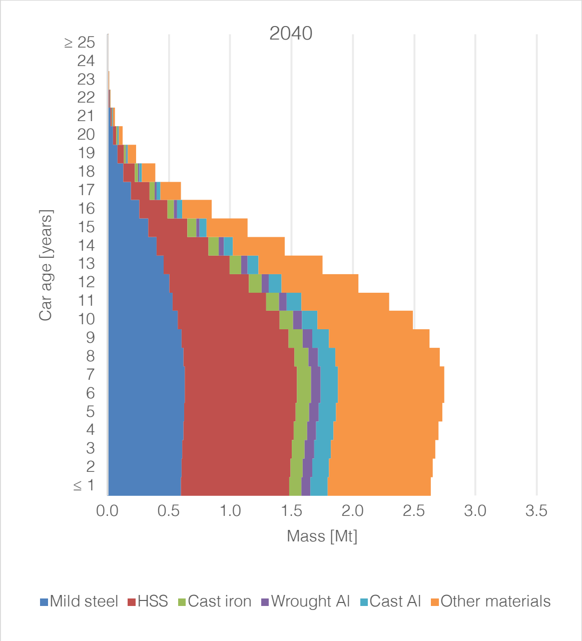
2040
Car age [years]
≥ 25
24
23
22
21
20
19
18
17
16
15
14
13
12
11
10
9
8
7
6
5
4
3
2
≤ 1
0.0
0.5
1.0
1.5
2.0
2.5
3.0
3.5
Mass [Mt]
Mild steel
HSS
Cast iron
Wrought Al
Cast Al
Other materials

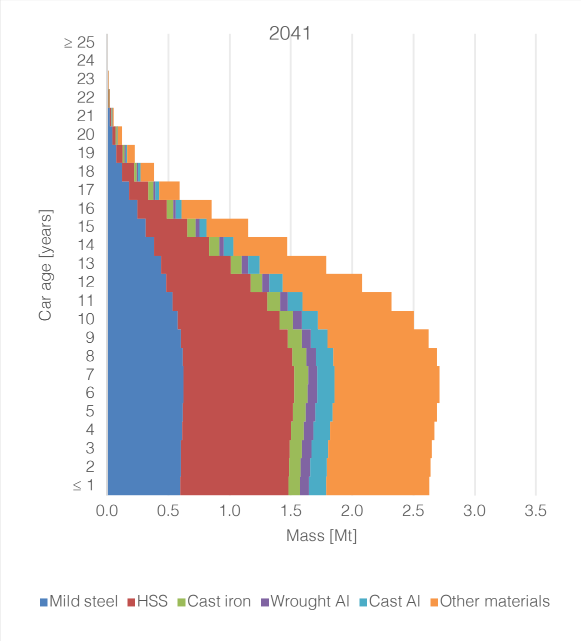
2041
Car age [years]
≥ 25
24
23
22
21
20
19
18
17
16
15
14
13
12
11
10
9
8
7
6
5
4
3
2
≤ 1
0.0
0.5
1.0
1.5
2.0
2.5
3.0
3.5
Mass [Mt]
Mild steel
HSS
Cast iron
Wrought Al
Cast Al
Other materials

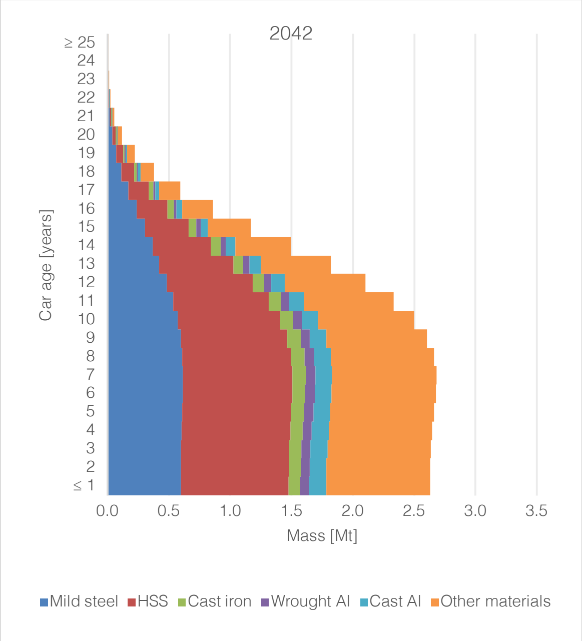
2042
Car age [years]
≥ 25
24
23
22
21
20
19
18
17
16
15
14
13
12
11
10
9
8
7
6
5
4
3
2
≤ 1
0.0
0.5
1.0
1.5
2.0
2.5
3.0
3.5
Mass [Mt]
Mild steel
HSS
Cast iron
Wrought Al
Cast Al
Other materials

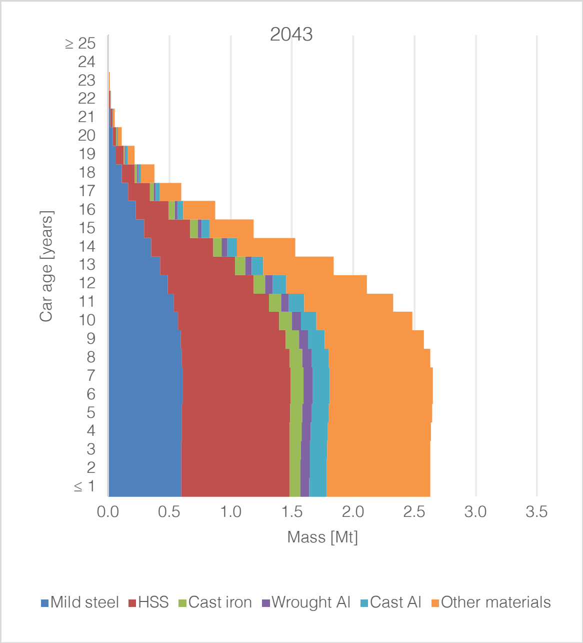
2043
Car age [years]
≥ 25
24
23
22
21
20
19
18
17
16
15
14
13
12
11
10
9
8
7
6
5
4
3
2
≤ 1
0.0
0.5
1.0
1.5
2.0
2.5
3.0
3.5
Mass [Mt]
Mild steel
HSS
Cast iron
Wrought Al
Cast Al
Other materials

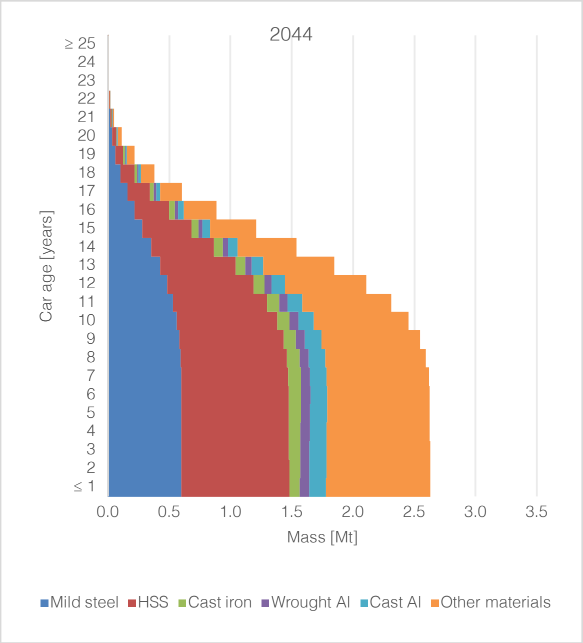
2044
Car age [years]
≥ 25
24
23
22
21
20
19
18
17
16
15
14
13
12
11
10
9
8
7
6
5
4
3
2
≤ 1
0.0
0.5
1.0
1.5
2.0
2.5
3.0
3.5
Mass [Mt]
Mild steel
HSS
Cast iron
Wrought Al
Cast Al
Other materials

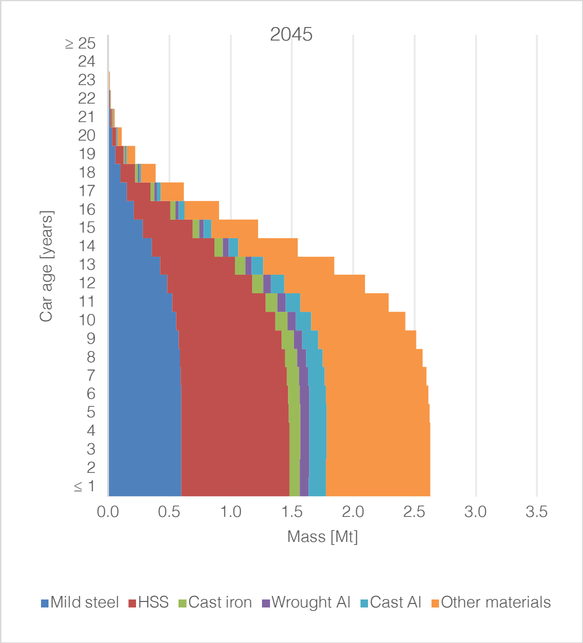
2045
Car age [years]
≥ 25
24
23
22
21
20
19
18
17
16
15
14
13
12
11
10
9
8
7
6
5
4
3
2
≤ 1
0.0
0.5
1.0
1.5
2.0
2.5
3.0
3.5
Mass [Mt]
Mild steel
HSS
Cast iron
Wrought Al
Cast Al
Other materials

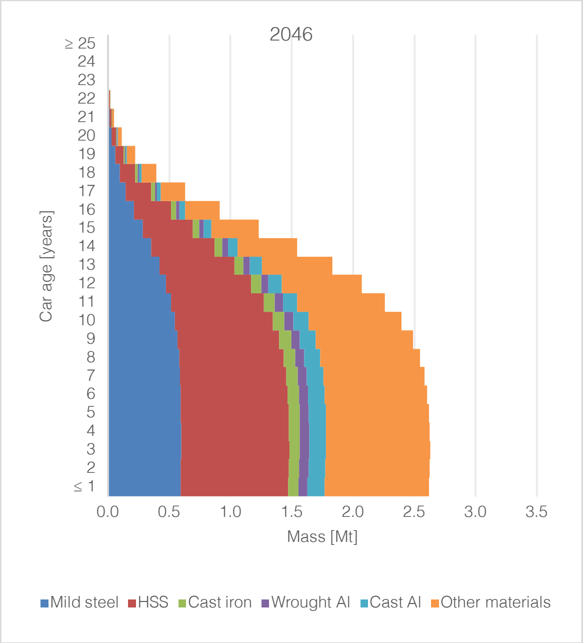
2046
Car age [years]
≥ 25
24
23
22
21
20
19
18
17
16
15
14
13
12
11
10
9
8
7
6
5
4
3
2
≤ 1
0.0
0.5
1.0
1.5
2.0
2.5
3.0
3.5
Mass [Mt]
Mild steel
HSS
Cast iron
Wrought Al
Cast Al
Other materials

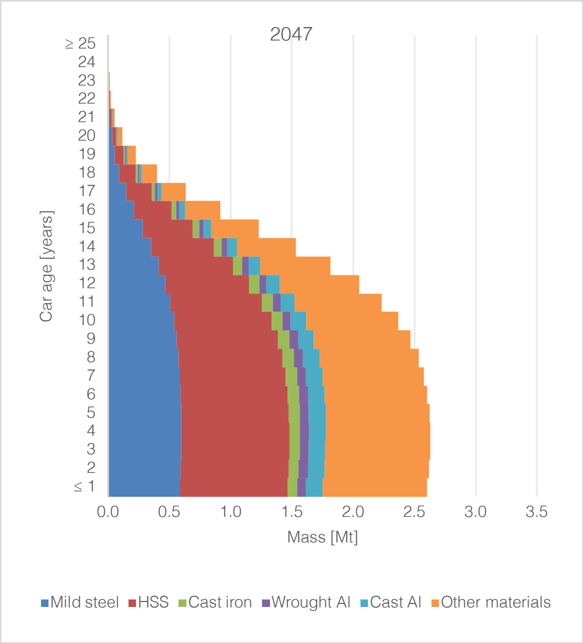
2047
Car age [years]
≥ 25
24
23
22
21
20
19
18
17
16
15
14
13
12
11
10
9
8
7
6
5
4
3
2
≤ 1
0.0
0.5
1.0
1.5
2.0
2.5
3.0
3.5
Mass [Mt]
Mild steel
HSS
Cast iron
Wrought Al
Cast Al
Other materials

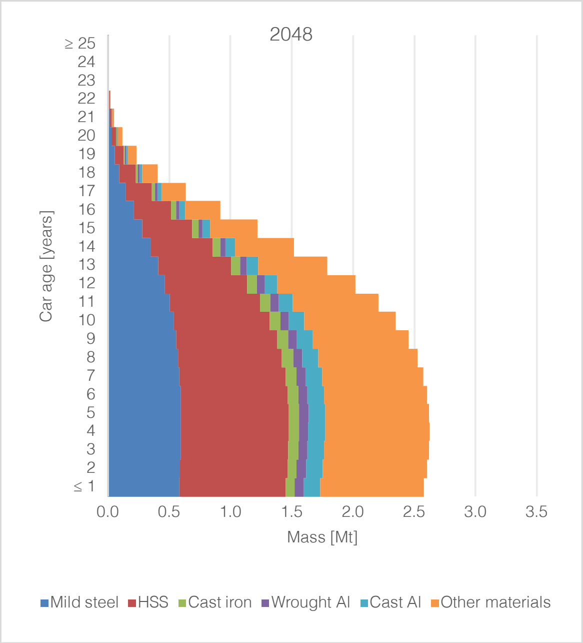
2048
Car age [years]
≥ 25
24
23
22
21
20
19
18
17
16
15
14
13
12
11
10
9
8
7
6
5
4
3
2
≤ 1
0.0
0.5
1.0
1.5
2.0
2.5
3.0
3.5
Mass [Mt]
Mild steel
HSS
Cast iron
Wrought Al
Cast Al
Other materials

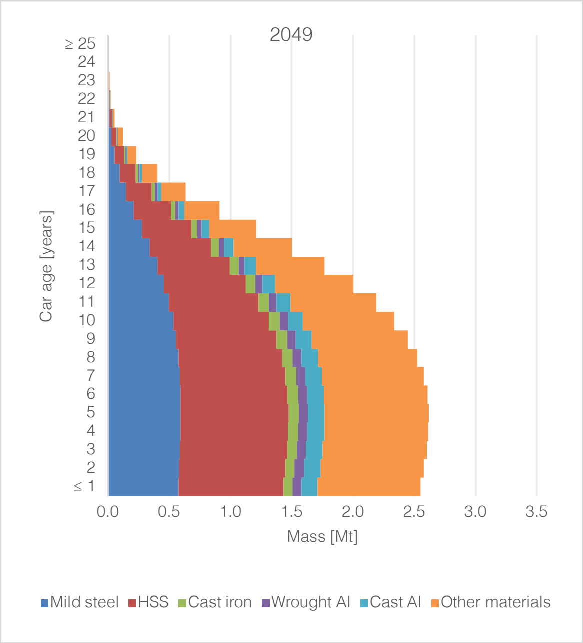
2049
Car age [years]
≥ 25
24
23
22
21
20
19
18
17
16
15
14
13
12
11
10
9
8
7
6
5
4
3
2
≤ 1
0.0
0.5
1.0
1.5
2.0
2.5
3.0
3.5
Mass [Mt]
Mild steel
HSS
Cast iron
Wrought Al
Cast Al
Other materials

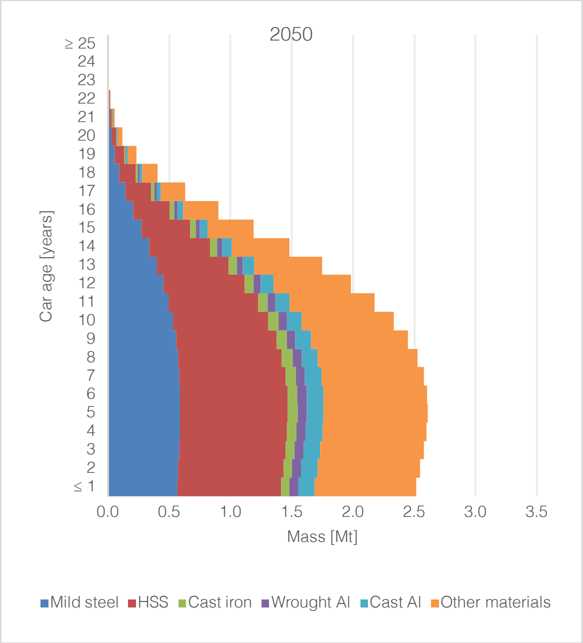
2050
Car age [years]
≥ 25
24
23
22
21
20
19
18
17
16
15
14
13
12
11
10
9
8
7
6
5
4
3
2
≤ 1
0.0
0.5
1.0
1.5
2.0
2.5
3.0
3.5
Mass [Mt]
Mild steel
HSS
Cast iron
Wrought Al
Cast Al
Other materials

8.3. New sales only comprised of EVs and PHEVs with steel-intensive material composition +20% of occupancy and intensity of use

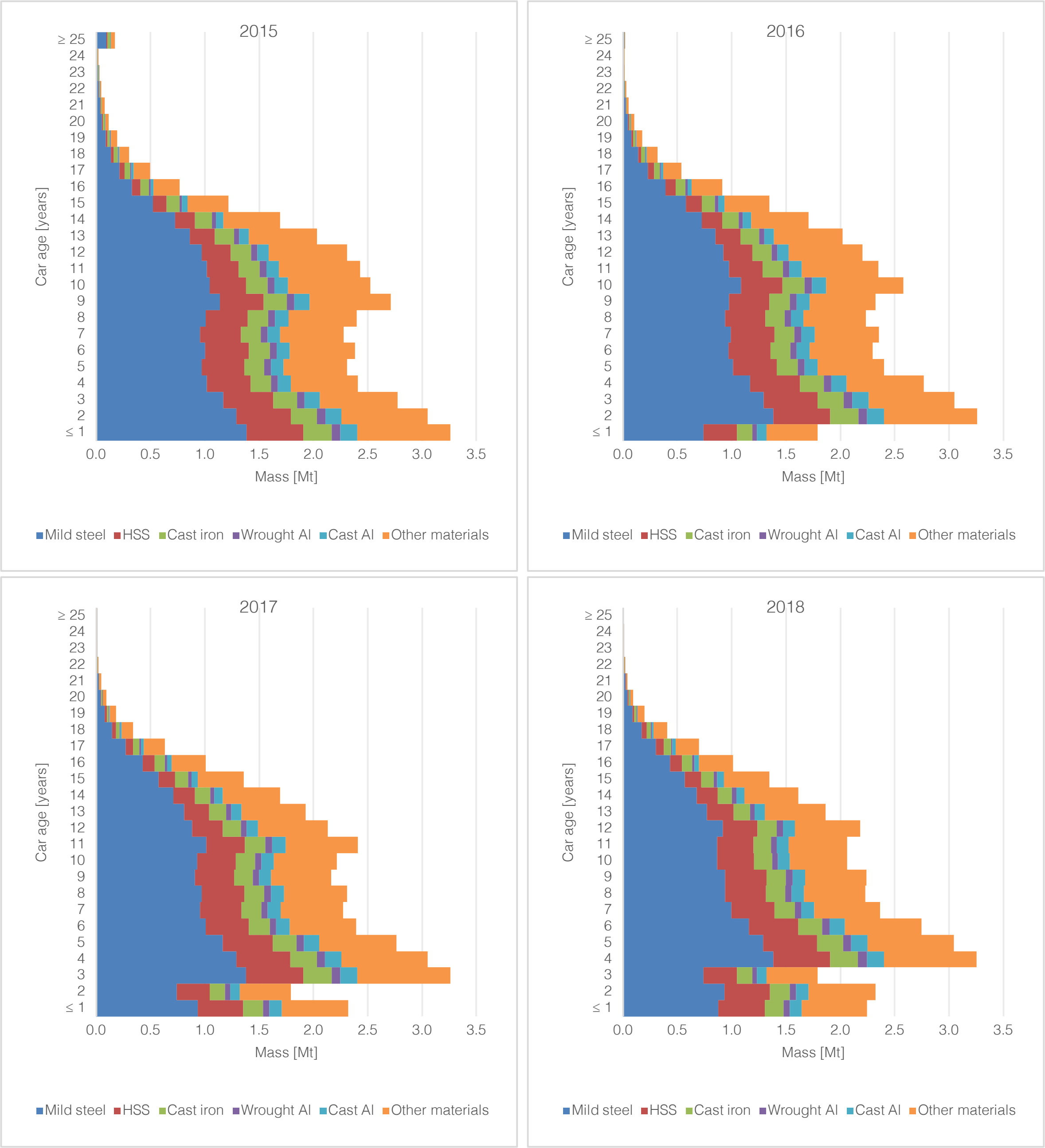

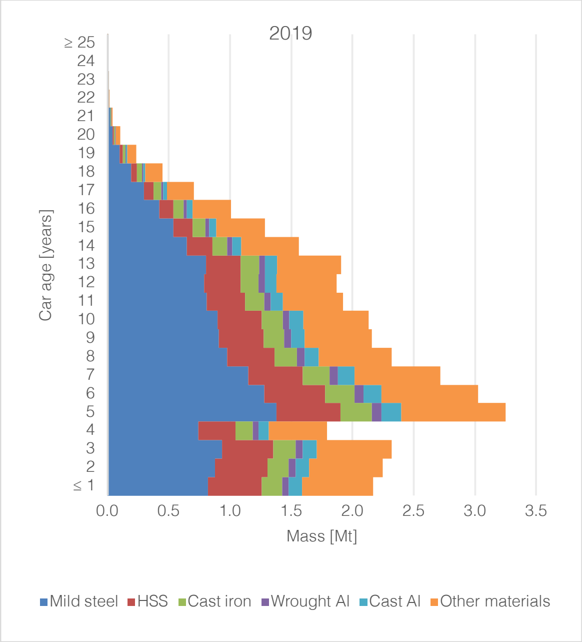
2019
Car age [years]
Mass [Mt]
Mild steel HSS Cast iron Wrought Al Cast Al Other materials

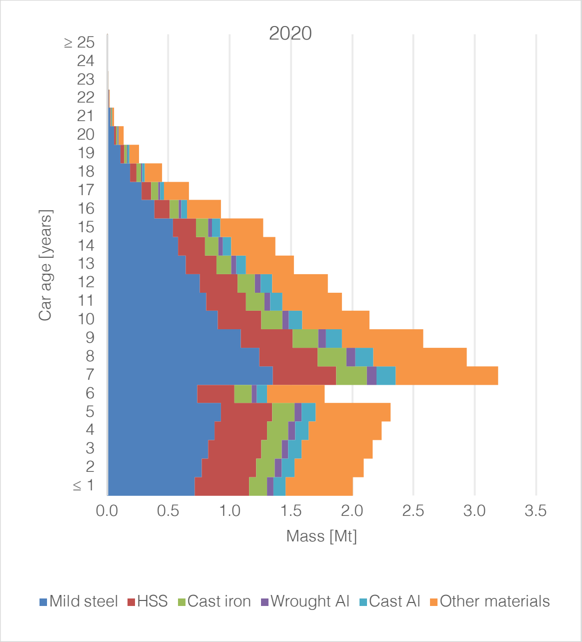
2020
Car age [years]
Mass [Mt]
Mild steel HSS Cast iron Wrought Al Cast Al Other materials

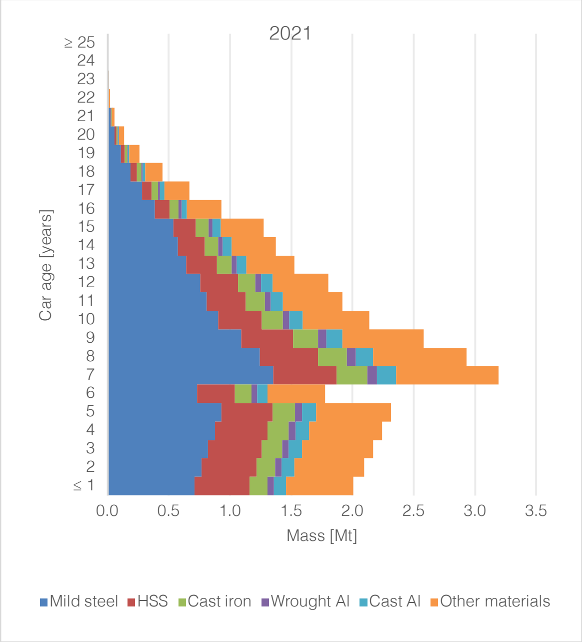
2021
Car age [years]
Mass [Mt]
Mild steel HSS Cast iron Wrought Al Cast Al Other materials

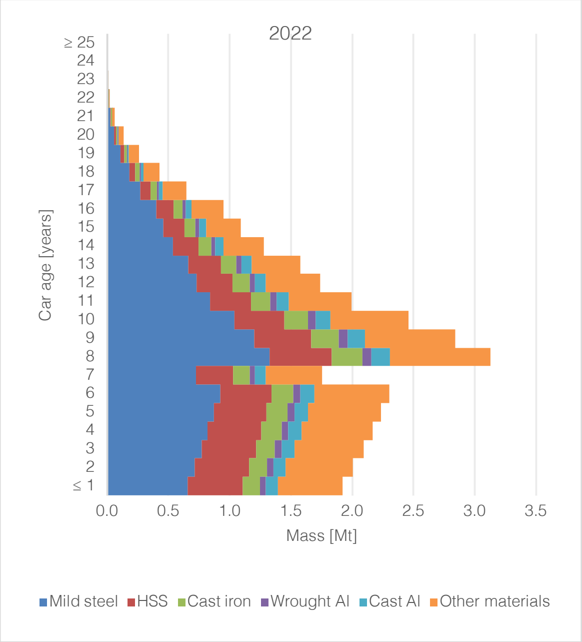
2022
Car age [years]
Mass [Mt]
Mild steel HSS Cast iron Wrought Al Cast Al Other materials

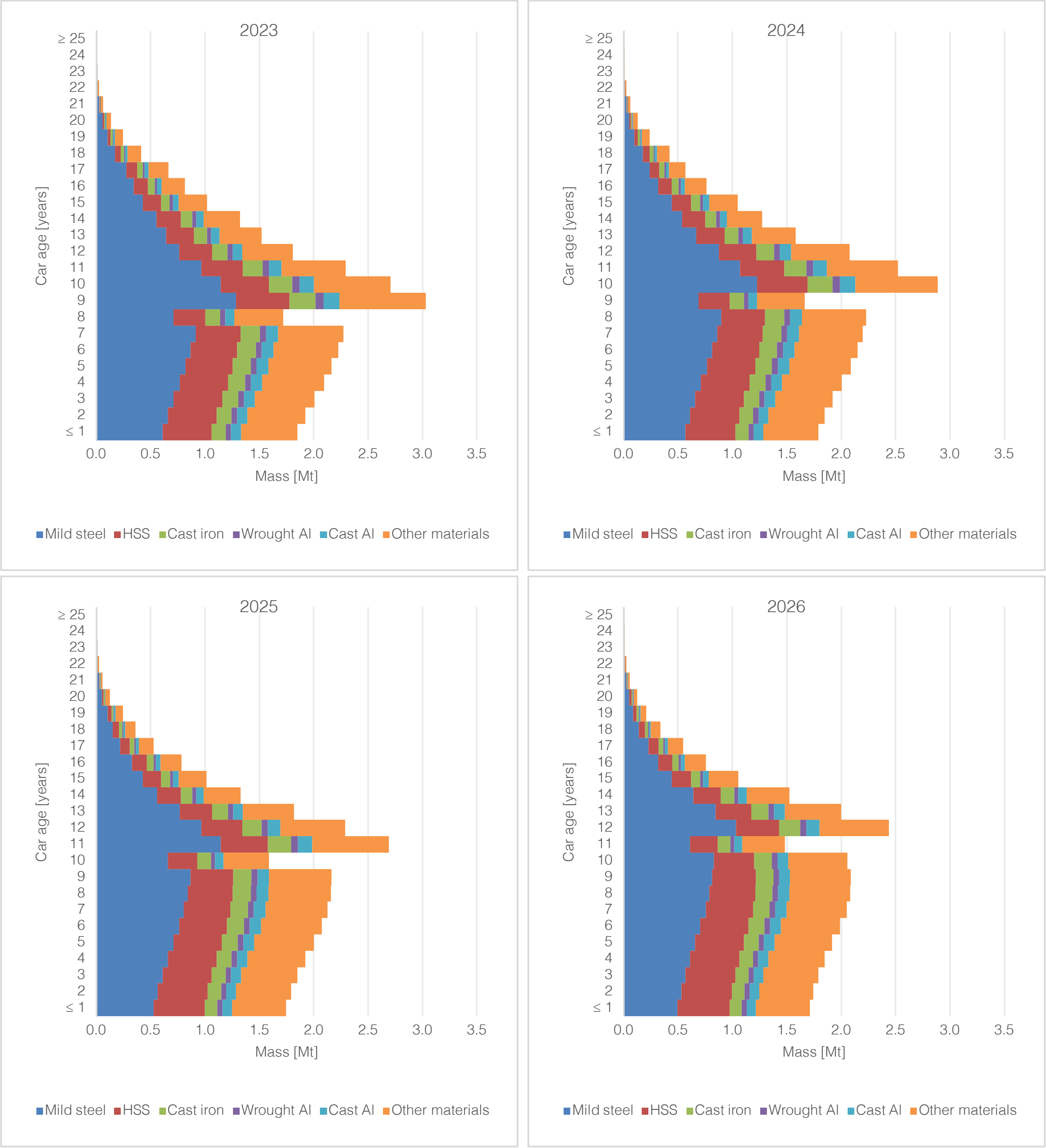
2023
2024
2025
2026
Car age [years]
≥ 25
≤ 1
Mass [Mt]
Mild steel
HSS
Cast iron
Wrought Al
Cast Al
Other materials

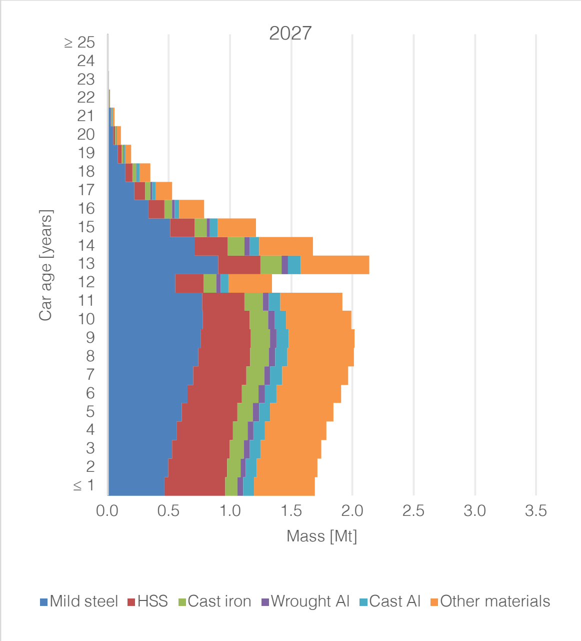
2027
≥ 25
24
23
22
21
20
19
18
17
16
15
14
13
12
11
10
9
8
7
6
5
4
3
2
≤ 1
Car age [years]
0.0
0.5
1.0
1.5
2.0
2.5
3.0
3.5
Mass [Mt]
Mild steel
HSS
Cast iron
Wrought Al
Cast Al
Other materials

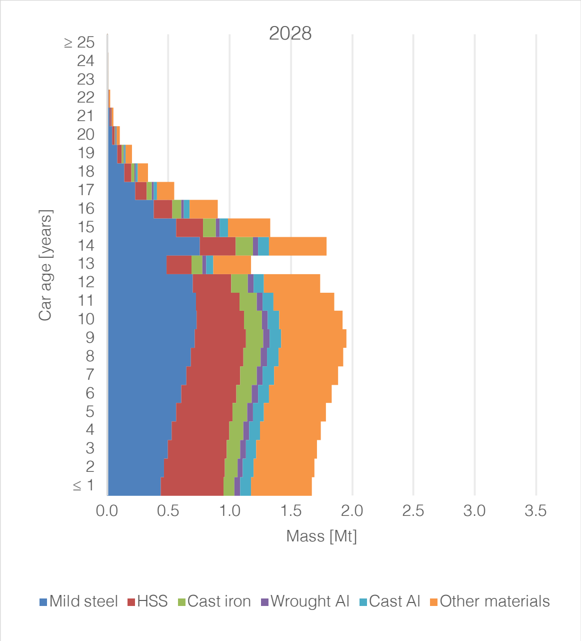
2028
≥ 25
24
23
22
21
20
19
18
17
16
15
14
13
12
11
10
9
8
7
6
5
4
3
2
≤ 1
Car age [years]
0.0
0.5
1.0
1.5
2.0
2.5
3.0
3.5
Mass [Mt]
Mild steel
HSS
Cast iron
Wrought Al
Cast Al
Other materials

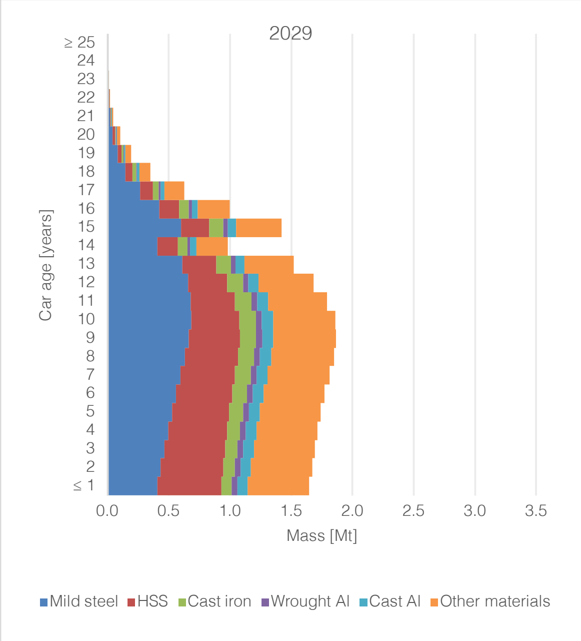
2029
≥ 25
24
23
22
21
20
19
18
17
16
15
14
13
12
11
10
9
8
7
6
5
4
3
2
≤ 1
Car age [years]
0.0
0.5
1.0
1.5
2.0
2.5
3.0
3.5
Mass [Mt]
Mild steel
HSS
Cast iron
Wrought Al
Cast Al
Other materials

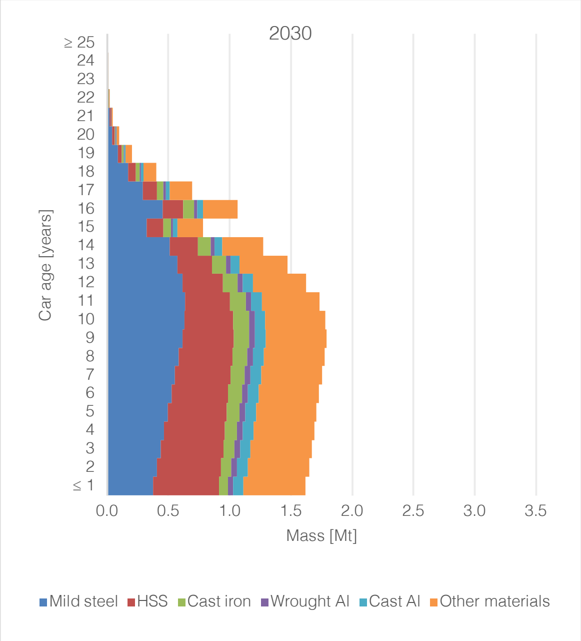
2030
≥ 25
24
23
22
21
20
19
18
17
16
15
14
13
12
11
10
9
8
7
6
5
4
3
2
≤ 1
Car age [years]
0.0
0.5
1.0
1.5
2.0
2.5
3.0
3.5
Mass [Mt]
Mild steel
HSS
Cast iron
Wrought Al
Cast Al
Other materials

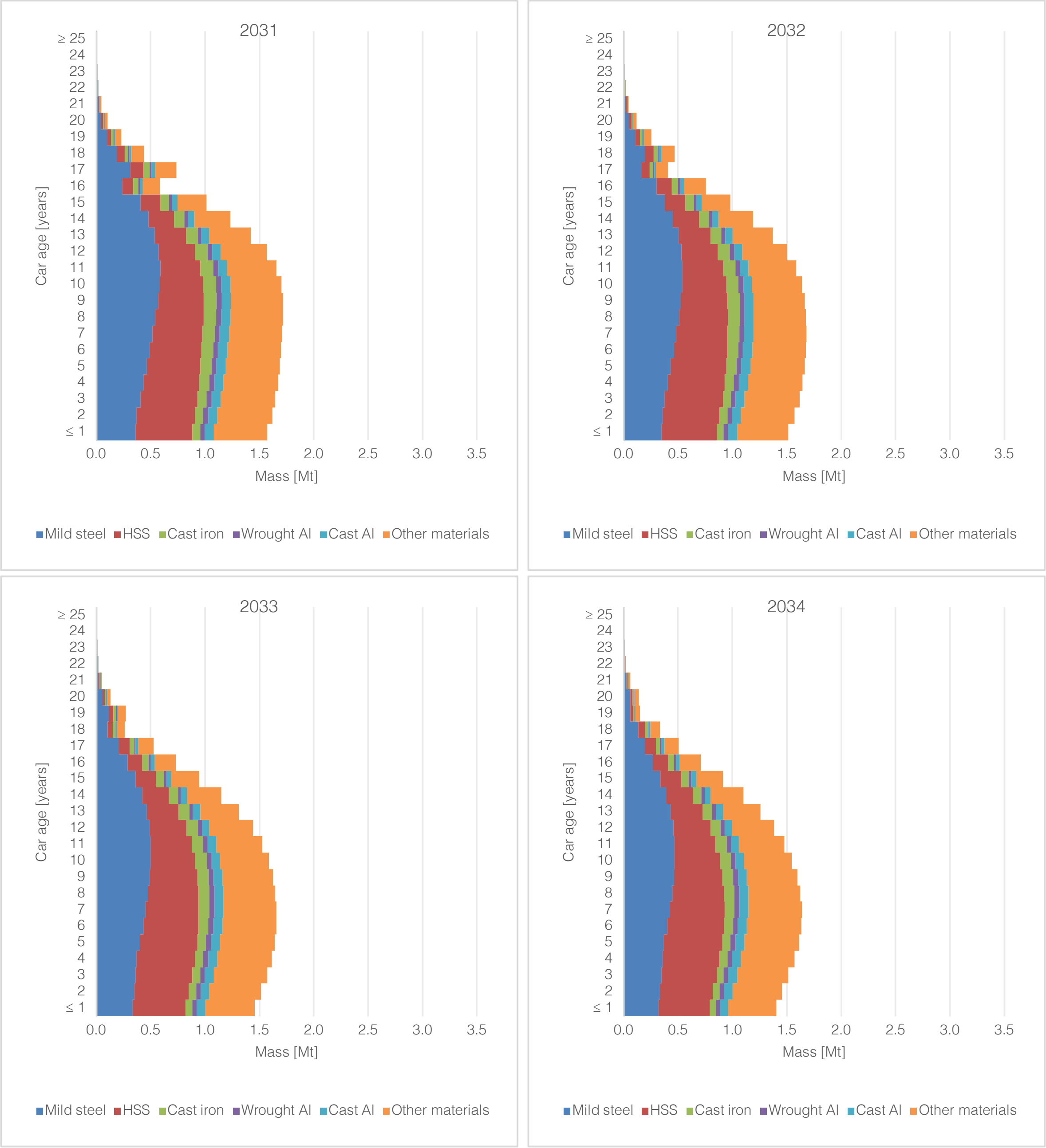
2031
2032
2033
2034
Car age [years]
≥ 25
≤ 1
Mass [Mt]
Mild steel
HSS
Cast iron
Wrought Al
Cast Al
Other materials

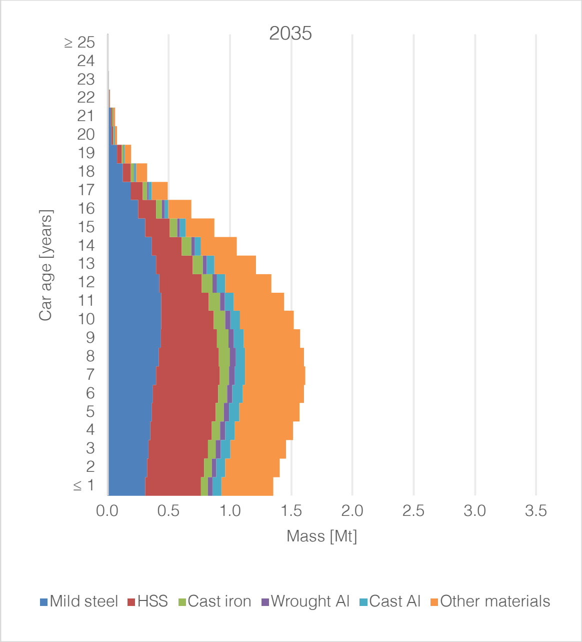
2035
Car age [years]
≥ 25
24
23
22
21
20
19
18
17
16
15
14
13
12
11
10
9
8
7
6
5
4
3
2
≤ 1
0.0
0.5
1.0
1.5
2.0
2.5
3.0
3.5
Mass [Mt]
Mild steel
HSS
Cast iron
Wrought Al
Cast Al
Other materials

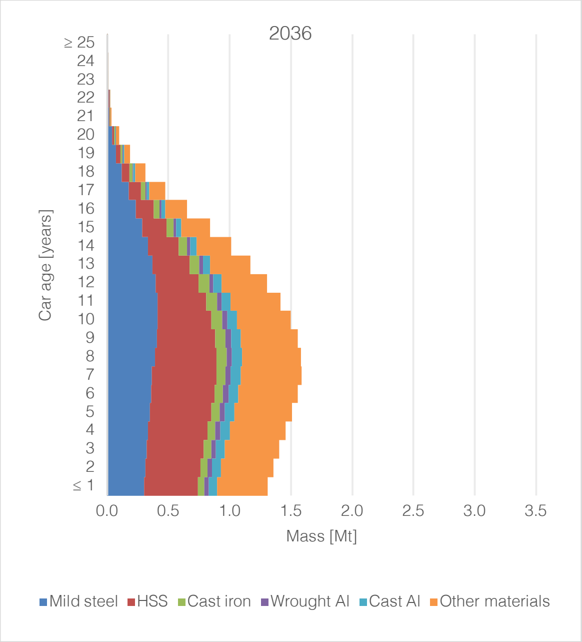
2036
Car age [years]
≥ 25
24
23
22
21
20
19
18
17
16
15
14
13
12
11
10
9
8
7
6
5
4
3
2
≤ 1
0.0
0.5
1.0
1.5
2.0
2.5
3.0
3.5
Mass [Mt]
Mild steel
HSS
Cast iron
Wrought Al
Cast Al
Other materials

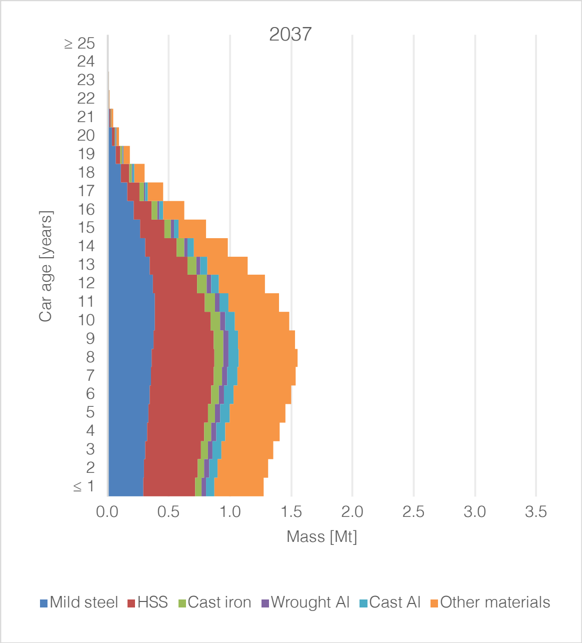
2037
Car age [years]
≥ 25
24
23
22
21
20
19
18
17
16
15
14
13
12
11
10
9
8
7
6
5
4
3
2
≤ 1
0.0
0.5
1.0
1.5
2.0
2.5
3.0
3.5
Mass [Mt]
Mild steel
HSS
Cast iron
Wrought Al
Cast Al
Other materials

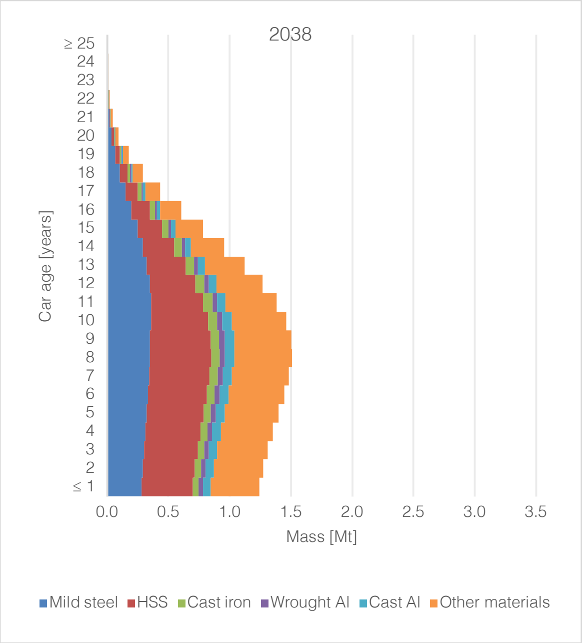
2038
Car age [years]
≥ 25
24
23
22
21
20
19
18
17
16
15
14
13
12
11
10
9
8
7
6
5
4
3
2
≤ 1
0.0
0.5
1.0
1.5
2.0
2.5
3.0
3.5
Mass [Mt]
Mild steel
HSS
Cast iron
Wrought Al
Cast Al
Other materials

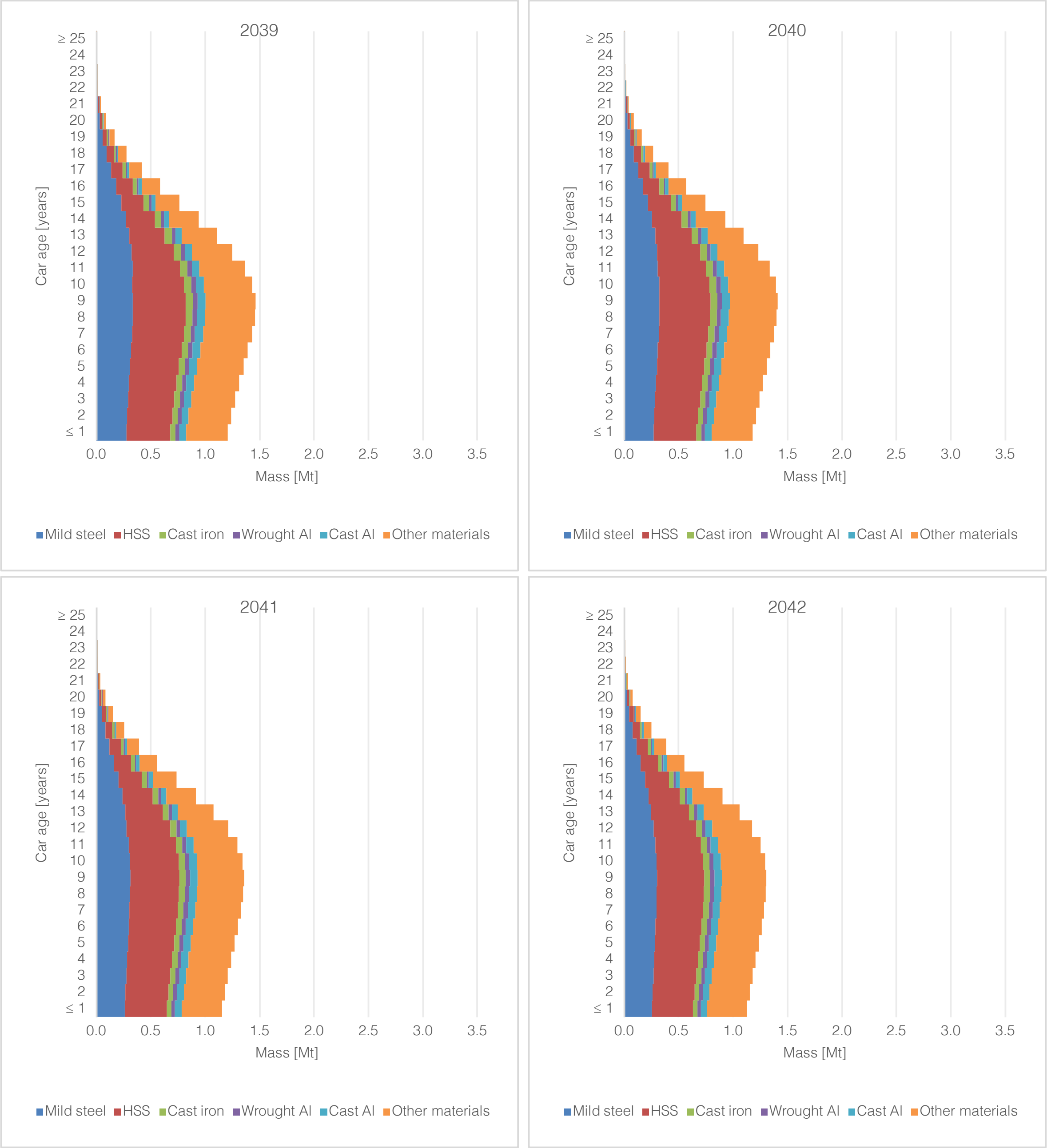
2039
2040
2041
2042
Car age [years]
≥ 25
24
23
22
21
20
19
18
17
16
15
14
13
12
11
10
9
8
7
6
5
4
3
2
≤ 1
0.0
0.5
1.0
1.5
2.0
2.5
3.0
3.5
Mass [Mt]
Mild steel
HSS
Cast iron
Wrought Al
Cast Al
Other materials

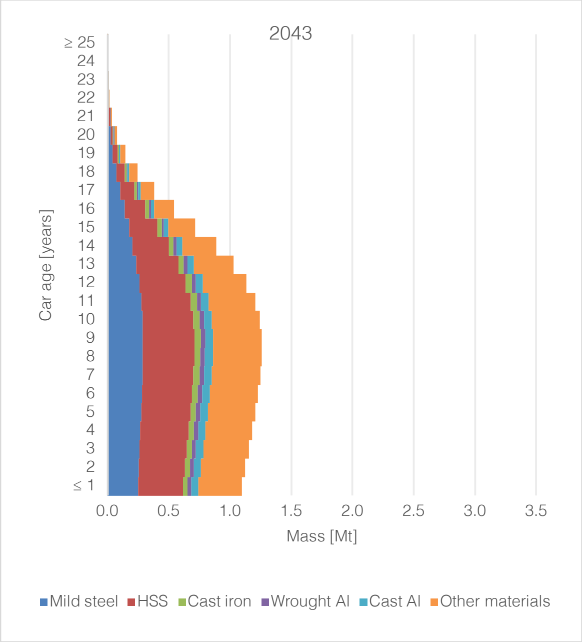
2043
Car age [years]
≥ 25
24
23
22
21
20
19
18
17
16
15
14
13
12
11
10
9
8
7
6
5
4
3
2
≤ 1
0.0 0.5 1.0 1.5 2.0 2.5 3.0 3.5
Mass [Mt]
Mild steel HSS Cast iron Wrought Al Cast Al Other materials

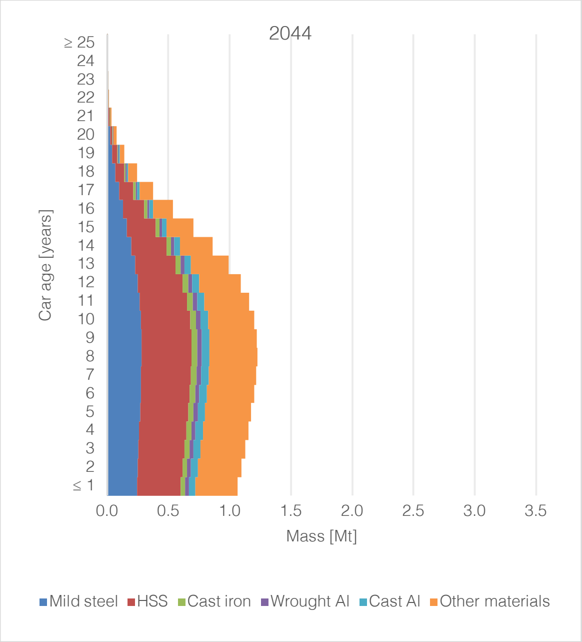
2044
Car age [years]
≥ 25
24
23
22
21
20
19
18
17
16
15
14
13
12
11
10
9
8
7
6
5
4
3
2
≤ 1
0.0 0.5 1.0 1.5 2.0 2.5 3.0 3.5
Mass [Mt]
Mild steel HSS Cast iron Wrought Al Cast Al Other materials

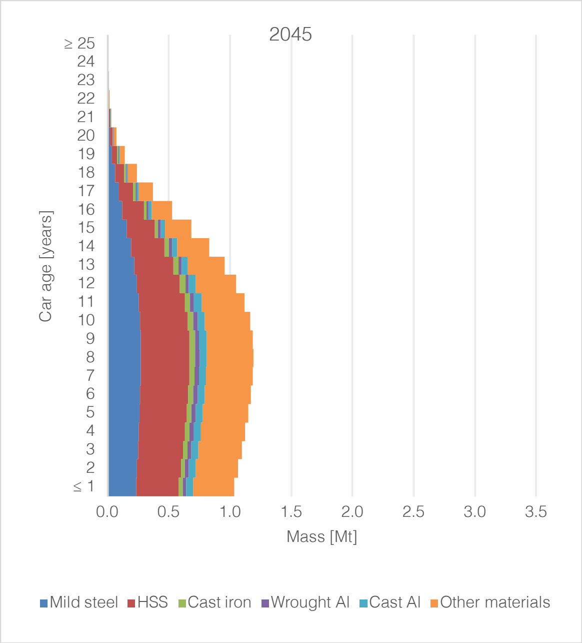
2045
Car age [years]
≥ 25
24
23
22
21
20
19
18
17
16
15
14
13
12
11
10
9
8
7
6
5
4
3
2
≤ 1
0.0 0.5 1.0 1.5 2.0 2.5 3.0 3.5
Mass [Mt]
Mild steel HSS Cast iron Wrought Al Cast Al Other materials

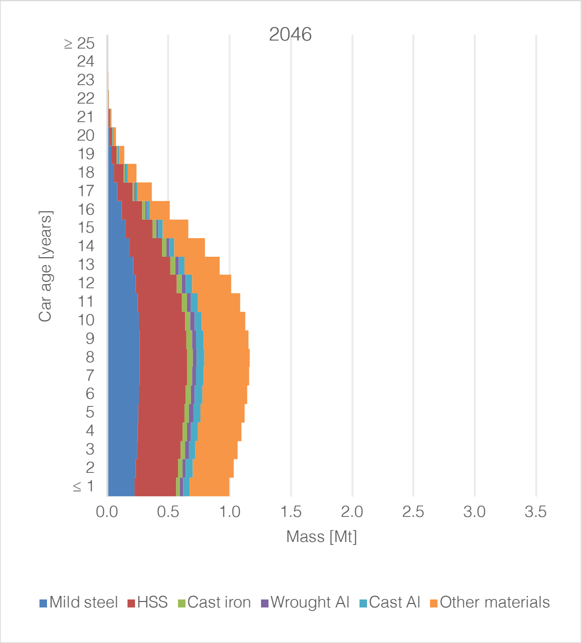
2046
Car age [years]
≥ 25
24
23
22
21
20
19
18
17
16
15
14
13
12
11
10
9
8
7
6
5
4
3
2
≤ 1
0.0 0.5 1.0 1.5 2.0 2.5 3.0 3.5
Mass [Mt]
Mild steel HSS Cast iron Wrought Al Cast Al Other materials

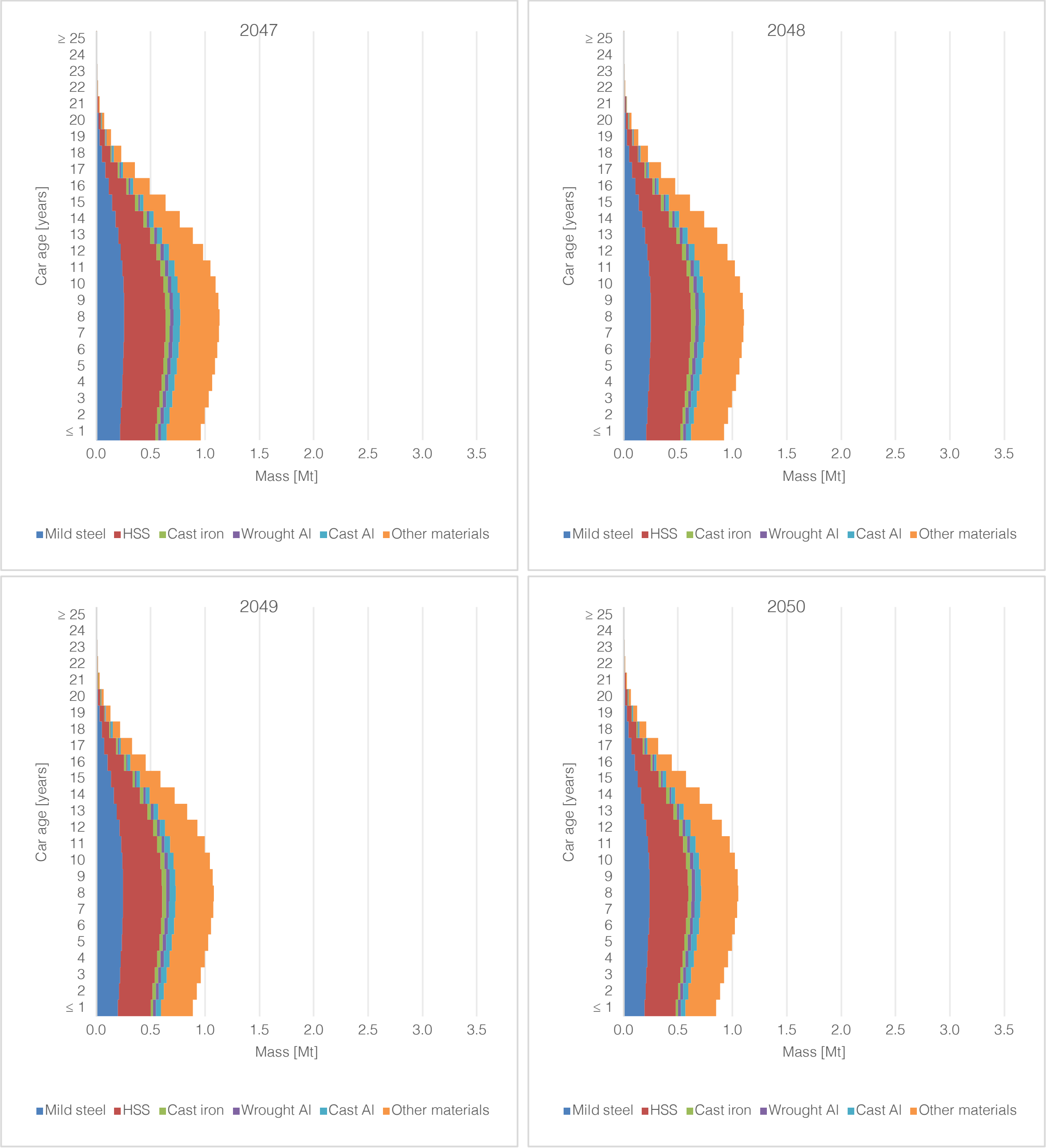
2047
2048
2049
2050
Car age [years]
≥ 25
24
23
22
21
20
19
18
17
16
15
14
13
12
11
10
9
8
7
6
5
4
3
2
≤ 1
0.0
0.5
1.0
1.5
2.0
2.5
3.0
3.5
Mass [Mt]
Mild steel
HSS
Cast iron
Wrought Al
Cast Al
Other materials

REFERENCES

ANL. *The Greenhouse Gases, Regulated Emissions, and Energy Use in Transportation (GREET) Model*. Chicago, IL, USA: Argonne National Laboratory, 2015.

CCC. *Sectoral scenarios for the fifth carbon budget - Technical report*. London, UK, 2015.

CHEAH, L.W. *Factor of Two: Halving the Fuel Consumption of New U.S. Automobiles by 2035*. MSc thesis in Mechanical Engineering. Cambridge, MA, USA: Massachusetts Institute of Technology, 2008.

CHEAH, L.W. *Cars on a Diet: The Material and Energy Impacts of Passenger Vehicle Weight Reduction in the U.S.* PhD thesis in Engineering Systems. Cambridge, MA, USA: Massachusetts Institute of Technology, 2010.

DECC. *Energy Consumption in the UK. Table 2.08: Fuel consumption for cars and lorries 1978 to 2011*. London, UK: Department of Energy and Climate Change,, 2015.

DEPARTMENT FOR TRANSPORT. *ENV0103 Average new car fuel consumption: Great Britain, 1997-2013*. London, UK: Department of Transport,, 2015.

DEPARTMENT FOR TRANSPORT. *National Travel Survey, 2002-2014 [computer file]*. Colchester, Essex, UK: UK Data Archive, 2016a.

DEPARTMENT FOR TRANSPORT. *Transport Statistics Great Britain*. London, UK, 2016b.

EUROPEAN COMMISSION, European Commission, [online] Reducing CO_2 emissions from passenger cars. 2015 [cited 4th August 2015]. Available from Internet: http://ec.europa.eu/clima/policies/transport/vehicles/cars/index_en.htm.

HM GOVERNMENT, DEFRA, Department for Environment Food & Rural Affairs [online] Greenhouse Gas Conversion Factor Repository. 2015. Available from Internet: http://www.ukconversionfactorscarbonsmart.co.uk/.

HYUNDAI, Hyundai [online] ix35 Fuel Cell. 2016 [cited 11/10/2016]. Available from Internet: http://worldwide.hyundai.com/WW/Showroom/Eco/ix35-Fuel-Cell/PIP/index.html.

ICCT, International Council on Clean Transportation [online] European Vehicle Market Statistics. 2016 [cited 22/06/2016]. Available from Internet: http://eupocketbook.theicct.org/.

IEA. *Energy Technology Perspectives 2010. Scenarios and Strategies to 2050.* Paris, France, 2010.

IEA. *CO2 Emissions from Fuel Combustion*. Paris, France: International Energy Agency, 2015a.

IEA. *World Energy Outlook 2015*. Paris, France: International Energy Agency, 2015b.

LEWIS, K. *Personal email with Statistical Officer of the Vehicle Statistics division in the Department for Transport*. 2016.

MOCK, P., GERMAN, J., BANDIVADEKAR, A., RIEMERSMA, I. *Discrepancies between type-approval and "real-world" fuel-consumption and CO_2 values. Assessment for 2001-2011 European passenger cars*. Washington, USA, 2012.

MOCK, P., TIETGE, U., FRANCO, V., GERMAN, J., BANDIVADEKAR, A., LIGTERINK, N., LAMBRECHT, U., KUHLWEIN, J., RIEMERSMA, I. *From Laboratory to Road. A 2014 Update of Official and "Real-World" Fuel Consumption and CO_2 Values for Passenger Cars in Europe*. Berlin, Germany, 2014.

MODARESI, R., PAULIUK, S., LØVIK, A.N., MÜLLER, D.B. *Global Carbon Benefits of Material Substitution in Passenger Cars until 2050 and the Impact on the Steel and Aluminum Industries*. Environmental Science & Technology, 2014, **48** (18): pp. 10776-10784.

OGUCHI, M., FUSE, M. *Regional and Longitudinal Estimation of Product Lifespan Distribution: A Case Study for Automobiles and a Simplified Estimation Method*. Environmental Science & Technology, 2015, **49** (3): pp. 1738-1743.

SCHÄFER, A., HEYWOOD, J.B., JACOBY, H.D., WAITZ, I.A. *Transportation in a Climate-Constrained World*. Cambridge, MA, USA: MIT Press, 2009. ISBN 978-0-262-01267-6.

SCHÄFER, A., VICTOR, D.G. *The future mobility of the world population*. Transportation Research Part A: Policy and Practice, 2000, **34** (3): pp. 171-205.

SCHIPPER, L., FIGUEROA, M.J., PRICE, L., ESPEY, M. *Mind the gap The vicious circle of measuring automobile fuel use*. Energy Policy, 1993, **21** (12): pp. 1173-1190.

SERRENHO, A.C., ALLWOOD, J.M. *Material Stock Demographics: Cars in Great Britain*. Environmental Science & Technology, 2016, **50** (6): pp. 3002-3009.

SPATH, P., MANN, M. *Life Cycle Assessment of Hydrogen Production via Natural Gas Steam Reforming*. Golden, Colorado, USA: National Renewable Energy Laboratory, 2001.

WOHLECKER, R., JOHANNABER, M., ESPIG, M. *Determination of weight elasticity of fuel economy for ICE, hybrid and fuel cell vehicles*. 2007.

ZACHARIADIS, T. *On the baseline evolution of automobile fuel economy in Europe*. Energy Policy, 2006, **34**: pp. 1773-1785.
